# Data-Independent Acquisition Proteomics Unravels the Effects of Iron Ions on Coronatine Synthesis in *Pseudomonas syringae* pv. *tomato* DC3000

**DOI:** 10.3389/fmicb.2020.01362

**Published:** 2020-07-21

**Authors:** Yan He, Sha Yu, Shaojin Liu, Hao Tian, Chunxin Yu, Weiming Tan, Jie Zhang, Zhaohu Li, Feng Jiang, Liusheng Duan

**Affiliations:** ^1^Engineering Research Center of Plant Growth Regulator, Ministry of Education & College of Agronomy and Biotechnology, China Agricultural University, Beijing, China; ^2^State Key Laboratory for Biology of Plant Diseases and Insect Pests, Institute of Plant Protection, Chinese Academy of Agricultural Sciences, Beijing, China; ^3^Engineering Research Center of Plant Growth Regulator, Ministry of Education & College of Horticulture, China Agricultural University, Beijing, China

**Keywords:** proteomics, iron ions, coronatine, *Pseudomonas syringae*, secondary metabolites

## Abstract

Coronatine (COR) is a new type of plant growth regulator that is produced by *Pseudomonas syringae* pathovars and plays an important role in modulating plant growth, development, and tolerance to multiple stresses. However, the factors affecting COR production are not very clear. In this study, the effects of FeCl_3_ on COR production were researched. The data-independent acquisition (DIA) approach, which is a proteomic quantitative analysis method, was applied to quantitatively trace COR production and proteomic changes in *P. syringae* pv. *tomato* DC3000 under different FeCl_3_ culture conditions. The results showed that COR production increased with the addition of FeCl_3_ and that there was significant upregulation in the expression of proteins related to COR synthesis and regulation. In addition, FeCl_3_ also affected the expression of related proteins involved in various metabolic pathways such as glycolysis and the tricarboxylic acid cycle. Moreover, various precursors such as isoleucine and succinate semialdehyde, as well as other related proteins involved in the COR synthesis pathway, were significantly differentially expressed. Our findings revealed the dynamic regulation of COR production in response to FeCl_3_ at the protein level and showed the potential of using the DIA method to track the dynamic changes of the *P. syringae* pv. *tomato* DC3000 proteome during COR production, providing an important reference for future research on the regulatory mechanism of COR biosynthesis and theoretical support for COR fermentation production.

## Introduction

Coronatine (COR) was isolated from the culture medium of *Pseudomonas syringae* pv. *atropurpurea* by Ichihara in 1977 (Ichihara et al., 1977). Coronatine is a small molecular secondary metabolite that has a cyclopentane structure and is a functional analog of jasmonoyl–isoleucine (JA–Ile) (Uppalapati et al., 2005). Coronatine has various physiological functions, and its activity is 10,000 times higher than that of JA–Ile (Katsir et al., 2008; Yan et al., 2016). Due to its ability to modulate plant growth, development, and responses to stress and to control plant diseases, COR and its derivatives serve as promising target compounds for agrochemical applications, including the development of novel herbicides and new types of plant growth regulators (Dayan and Duke, 2014; Zhou et al., 2015, 2018; Xu et al., 2020). Coronatine is produced by several members of the *P. syringae* group of pathovars, including the pathovar tomato DC3000 (Geng et al., 2014; Bignell et al., 2018). *P. syringae* pv. *tomato* DC3000 is a Gram-negative bacterium that exists widely in a variety of ecosystems and is a plant pathogen that infects both tomato and *Arabidopsis thaliana* (Xin and He, 2013). Because the genome of *P. syringae* pv. *tomato* DC3000 has been sequenced and reported, it is often studied as a model strain, and COR is one of the major virulence factors produced by *P. syringae* pv. *tomato* DC3000, causing chlorosis in plants (Bender et al., 1999; Buell et al., 2003; Thilmony et al., 2006).

Microorganisms produce a variety of bioactive compounds as secondary metabolites that can be used to develop new plant growth regulators and biomedicine (Gouda et al., 2016; Oliveira et al., 2016). Determining the signals that trigger the biosynthesis of secondary metabolites allows us to understand the ecological role of secondary metabolites within microbial communities and ultimately to increase the industrial production of these metabolites. Previous studies have identified several regulatory proteins related to COR synthesis. Coronatine is a secondary metabolite, so it is also regulated by specific trace elements, inorganic phosphorus, temperature, and pH (Wang et al., 2002; Sreedharan et al., 2006). The concentration of iron is very important for the biosynthesis of secondary metabolites, and the iron concentration required usually exceeds that required for cell growth (Rahme et al., 1992). The mechanism underlying the effects on COR production of chemical and physical factors used as environmental factors is not clear, and iron plays an important role under many related ecological conditions (Saha et al., 2015). Therefore, understanding the effects of iron on *P. syringae* pv. *tomato* DC3000 will be useful for research on the physiology of this organism in its natural environment. In addition, COR is obtained mainly from microbial fermentation, and studying the effects of environmental factors on the yield of COR is highly important for the application of COR in fermentation production.

Iron is an essential element required for the growth of nearly all living microorganisms because it acts as a coenzyme factor of many redox enzymes involved in respiration, DNA biosynthesis, gene regulation, the tricarboxylic acid (TCA) cycle, and other metabolic processes (Aguado-Santacruz et al., 2012; Porcheron et al., 2013; Porcheron and Dozois, 2015). Most bacteria need a micromolar concentration of iron to grow optimally (Andrews et al., 2003). Bacteria have evolved a series of mechanisms that enable them to obtain iron from the environment, including the ability to reduce iron to ferrous ions and to absorb iron bound to siderophores (microbial iron chelates) (Pattus and Abdallah, 2000). Iron uptake mediated by siderophores is probably the most common form of iron uptake in bacteria, the process of which is important for bacterial survival and growth (Ringel and Brüser, 2018). Studies have shown that iron restriction affects the growth of *Escherichia coli* cells in culture and that iron can alter the physiology of other *Pseudomonas* bacteria in both batch culture and thermostat culture (Kim et al., 2005; Ongena et al., 2008). Kim et al. (2009) studied the effects of iron concentration on the growth rate of *P. syringae* and the expression of virulence factors induced by *hrp* for hypersensitive response and pathogenicity (Kim et al., 2009). In addition, Bronstein et al. (2008) examined the global transcriptional responses of *P. syringae pv. tomato* DC3000 to changes in iron. Iron has been shown to regulate the expression of 21 secondary metabolite biosynthesis gene clusters in *Streptomyces* (Lee et al., 2020), and two studies have shown that the production of the phytotoxins syringomycin, and syringotoxin, which are secondary metabolites from *P. syringae*, significantly increased in media supplemented with iron ions (Gross, 1985; Morgan and Chatterjee, 1988). These studies show that iron not only promotes the growth of *P. syringae* but also participates in the regulation of secondary metabolite biosynthesis. However, the potential molecular mechanism underlying iron regulation is very complex.

Recently, a new mass spectrometry (MS) analysis approach called data-independent acquisition (DIA) was proposed for analyzing proteins; DIA is considered a promising approach for high-throughput quantitative proteome profiling (Gillet et al., 2012). It has been demonstrated that DIA can be used to identify and quantify thousands of proteins without the need for fractionation, and only a few micrograms of a protein sample is needed (Fan et al., 2019; Lou et al., 2020; Wang et al., 2020). The main purpose of the present study was to gain insight into the proteomics of COR biosynthesis in response to iron ion addition in fermentation. Using liquid chromatography-tandem mass spectrometry (LC-MS/MS) in DIA mode, we quantitatively analyzed the expression of proteins under different FeCl_3_ treatments and evaluated the effects of FeCl_3_ on COR biosynthesis. We investigated the possibility that FeCl_3_ could regulate the expression of proteins related to COR synthesis and regulation and could affect metabolic pathways such as glycolysis and the TCA cycle. In the end, we revealed the regulatory effects of iron ions on COR production at the protein level.

## Materials and Methods

### Strain Culture and Sampling

*P. syringae* pv. *tomato* DC3000 was used to determine the effects of FeCl_3_ on COR production. The strain was cultured in MG liquid media at 28°C under shaking at 200 rpm until the seed liquid reached an OD_600_ of 1.2–1.5, after which the seed liquid was inoculated into the fermentation culture in GC liquid media. The inoculum was 2% (v/v) and was cultured at 18°C at 200 rpm. Samples were taken at 10 days after inoculation to measure COR production when the yield was stable. The MG medium component contained the following nutrients per liter: 10 g of mannitol, 0.5 g of KH_2_PO_4_, 2 g of L-monosodium glutamate, 0.2 g of NaCl, and 0.2 g of MgSO_4_⋅7H_2_O (pH 7.0). Eighteen grams of agar was added to the solid media. The GC medium component contained the following nutrients per liter: 1.0 g of NH_4_Cl, 3.6 g of K_2_HPO_4_, 4.1 g of KH_2_PO_4_, 0.2 g of MgSO_4_⋅7H_2_O, 0.3 g of KNO_3_, and 20 g of glycerol (pH 6.8). Sterilized FeCl_3_ solution was added before inoculation. The concentration of FeCl_3_ in the fermentation medium of the control group was 0 μmol/L (0Fe); one treatment group involved 20 μmol/L FeCl_3_ (DC), and the other treatment group involved 400 μmol/L FeCl_3_ (HFe). Samples were collected at 4, 8, and 10 days after inoculation, and the protein groups were sequenced for three biological repeats in each group.

### Extraction and Detection of Coronatine

A total of 1.5 mL of fermentation liquid was added to a 2-mL centrifuge tube, after which the fermentation broth pH was brought to 2.5–2.9. The liquid was then centrifuged at 8000 rpm for 10 min. A 1-mL sample of the supernatant was removed to a new centrifuge tube, and 1 mL of petroleum ether was added. The extraction was shaken for 10 s and was then left to stand for 2 min. Afterward, the upper organic phase was removed, and 1 mL of ethyl acetate was added. The extraction was subsequently shaken for 10 s and then allowed to sit for 2 min, after which the ethyl acetate phase was transferred to a new centrifuge tube. The extractions were repeated three times, and each extract was subsequently dissolved in 1 mL of methanol after airdrying naturally or being dried under a stream of nitrogen. These final samples were ultimately tested and used for the determination of COR. The content of COR was measured via high-performance liquid chromatography (HPLC). The mobile phase was composed of methanol and water (containing 0.5% acetic acid) at a 6.5:3.5 volumetric ratio, the detection wavelength was 220 nm, the flow rate was 1 mL/min, the injection volume was 20 μL, and the sample retention time was 30 min. The content of COR in the extract was determined on the basis of the retention time of COR standard, and the production of COR was determined by comparisons with the peak area of the COR standard.

### Protein Extraction and Digestion

Lysis buffer [1% SDS, 8 M urea, 1x Protease Inhibitor Cocktail (Roche Ltd. Basel, Switzerland)] was added to the 27 samples, which were subsequently vibrated and milled for 400 s three times. The samples were then lysed on ice for 30 min and centrifuged at 15,000 rpm for 15 min at 4°C. The supernatant was collected and transferred to a new Eppendorf tube.

Then, 100 μg of protein per condition was transferred to a new tube and the final volume was adjusted to 100 μL with 8 M urea. Next, 2 μL of 0.5 M Tris (2-carboxyethyl) phosphine (TCEP) was added, and the sample was incubated at 37°C for 1 h. Afterward, 4 μL of 1 M iodoacetamide was added to the sample, which was then incubated for 40 min in darkness at room temperature. Afterward, five volumes of −20°C pre-chilled acetone were added to precipitate the proteins overnight at −20°C. The precipitates were washed twice with 1 mL of pre-chilled 90% acetone aqueous solution and then redissolved in 100 μL 100 mM tetraethylammonium bromide (TEAB). Sequence-grade modified trypsin (Promega, Madison, WI) was added at a ratio of 1:50 (enzyme: protein, weight: weight) to digest the proteins at 37°C overnight. The peptide mixture was desalted by C18 ZipTip, quantified by Pierce^TM^ Quantitative Colorimetric Peptide Assay (23275), and then lyophilized by Thermo Fisher Scientific Savant SpeedVac. Nine group samples were selected, and each group was represented by three biological replicates. For library generation via the data-dependent acquisition (DDA) approach, all 27 samples were pooled as a mixture and fractionated by high-pH separation into six fractions. All 27 samples were processed by DIA individually to assess the proteomic differences. MS1 and MS2 data were acquired, and sample data were acquired in random order. An iRT kit (Ki3002, Biognosys AG, Switzerland) was used to calibrate the retention time of the peaks of the extracted peptides from all of the samples.

### Liquid Chromatography-Mass Spectrometry Analysis

The peptides were redissolved in 5% acetonitrile (ACN) aqueous solution containing 0.5% formic acid and analyzed by on-line nanospray LC-MS/MS with a Q Exactive^TM^ HF-X spectrometer coupled to the EASY-nLC 1200 system (Thermo Fisher Scientific, MA, United States). A 3-μL peptide sample was loaded (trap column, Thermo Fisher Scientific Acclaim PepMap C18, 100 μm × 2 cm; analytical column, Acclaim PepMap C18, 75 μm × 25 cm) and separated with a 60 min-gradient, from 6 to 60% B (B: 0.1% formic acid, 80% ACN). The column flow rate was maintained at 250 nL/min. An electrospray voltage of 2 kV (at the inlet of the mass spectrometer) was used.

The mass spectrometer was run in DDA mode and automatically switched between MS and MS/MS modes. The parameters were as follows: (1) MS: scan range (*m*/*z*) = 375–1500, resolution = 60,000, AGC target = 3e6, maximum injection time = 20 ms, include charge states = 2–6, and dynamic exclusion = 30 s; (2) HCD-MS/MS: resolution = 15,000, isolation window = 1.2, AGC target = 1e5, maximum injection time = 30 ms, and collision energy = 27.

The mass spectrometer was then run in DIA mode and automatically switched between MS and MS/MS modes. The parameters were as follows: (1) MS: scan range (*m*/*z*) = 350–1250, resolution = 120,000, AGC target = 3e6, and maximum injection time = 20 ms; (2) HCD-MS/MS: resolution = 30,000, AGC target = 1e6, and collision energy = 25.5, 27, 30; (3) DIA was performed with a variable isolation window, each window overlapped by 1 m/z, there were 60 windows in total, and the total cycle time was 3 s.

### Data Analysis for LC-MS

The raw DDA data were processed and analyzed by Spectronaut 13 (Biognosys AG, Switzerland) with the default settings to generate an initial target list that contained 34,838 peptides and 3,918 proteins. Spectronaut 13 was set up to search the database of *P. syringae* pv. *tomato* DC3000 (strain ATCC BAA-871) from UniProt (ver 201909, 5431 entries) assuming trypsin as the digestion enzyme. A Q value (false discovery rate, FDR) cutoff of 1% was applied at the precursor and protein levels. The raw DIA data were processed and analyzed by Spectronaut 13 (Biognosys AG, Switzerland) with the default settings, and the retention time prediction type was set to dynamic iRT. Data extraction was determined by Spectronaut 13 on the basis of extensive mass calibration. Spectronaut determines the ideal extraction window dynamically on the basis of iRT calibration and gradient stability. A Q value (FDR) cutoff of 1% was applied at the precursor and protein levels. Decoy generation was set to mutated, which is similar to scrambled but only applied a random number of AA position swamps (min = 2, max = length/2). All selected precursors passing the filters were used for quantification. MS2 interference removed all interfering fragment ions except for the three least interfering ones. The average top 3 filtered peptides that passed the 1% Q value cutoff were used to calculate the major group quantities.

### Analysis of Quantitative Data

Only proteins detected and quantified in all runs (three biological replicates) were included in the data set. The Student’s *t*-test was performed to test for significance. After Welch’s ANOVA test, proteins were considered differentially expressed if their *p*-value was <0.05 and fold change was >2 or <0.5; those that met these criteria were defined as being “significantly” regulated.

## Results and Discussion

### Influence of FeCl_3_ Addition on the Growth of *P. syringae* pv. *tomato* DC3000 and Its Coronatine Synthesis

**FIGURE 1:**
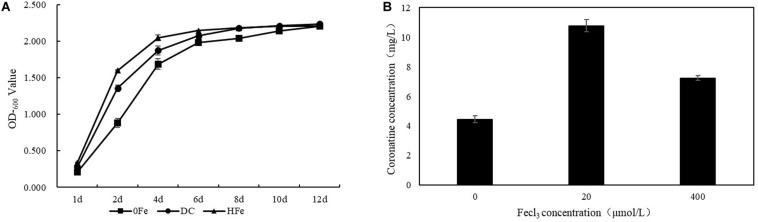
The OD_600_ value and the coronatine production of DC3000 under different FeCl_3_ concentration fermentation conditions. **(A)** The OD_600_ values of the three groups researched in the study were detected by ultraviolet spectrophotometer at OD_600_ after inoculation in fermentation medium. **(B)** The coronatine production of the three groups was measured at 10 days after inoculation in fermentation when the yield of the fermentation culture was stable. The mean was taken from three biologically independent replicates.

To evaluate the cellular response to FeCl_3_ addition in terms of COR biosynthesis, *P. syringae* pv. *tomato* DC3000 was cultured. Bacterial growth and COR synthesis were monitored during culture, and the growth curve of strain *P. syringae* pv. *tomato* DC3000 and the final steady production of COR at 10 days after inoculation in the three groups of fermentation cultures were measured (Figure 1). The FeCl_3_ concentrations tested involved different gradients that ranged from 0 to 1,000 μmol/L in our previous experiments (data not provided). In the present study, we chose the most suitable concentration for fermentation, 20 μmol/L, and COR yields were not significantly different when the concentration was higher than 400 μmol/L. Compared with that in the 0Fe group, the OD_600_ value in the treatment groups adding FeCl_3_ changed more rapidly at the early stage of fermentation. The OD_600_ values were 1.356 ± 0.05 and 1.600 ± 0.01 in the two treatment groups with FeCl_3_ added and only 0.882 ± 0.06 in the 0Fe group, and the OD_600_ values were similar at the end of fermentation, reaching approximately 2.200. Therefore, we can infer that the growth of strain *P. syringae* pv. *tomato* DC3000 in the 0Fe group was slower than that in the treatment group with FeCl_3_ added. Moreover, compared to that in the 0Fe group, the production of COR in the group with FeCl_3_ was higher. In the DC group, the production of COR was 10.78 ± 0.4 mg/L, which was more than twice that in the 0Fe group (4.44 ± 0.2 mg/L). The production of COR in the HFe group was also higher than that in the 0Fe group, reaching approximately 7.24 ± 0.2 mg/L, but it was lower than that in the DC group. These results suggest that a high concentration of FeCl_3_ is not suitable for COR fermentation. On the basis of the results, we can conclude that the addition of FeCl_3_ in the fermentation medium affects the growth of *P. syringae* pv. *tomato* DC3000 and increases the biosynthesis of COR and that COR production is associated with the growth of *P. syringae* pv. *tomato* DC3000 cells under the FeCl_3_ fermentation conditions applied.

### DIA Proteomic Identification of *P. syringae* pv. *tomato* DC3000

**FIGURE 2:**
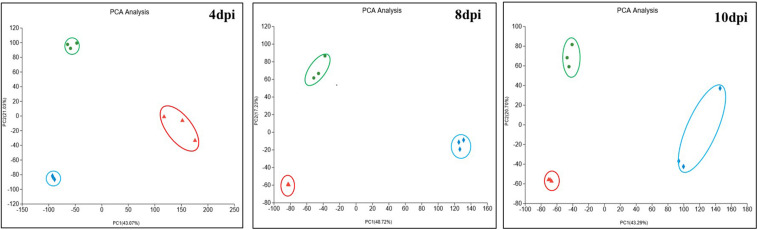
Principal component analysis (PCA) of the samples from the three groups at 4, 8, and 10 dpi used ropls (R, Version 1.6.2) in the stats package with default parameters. Blue diamonds represent samples without added FeCl_3_ (0Fe), green circles represent the treatment group with 20 μmol/L FeCl_3_ added (DC), and red triangles represent the treatment group with 400 μmol/L FeCl_3_ added (HFe).

To understand the whole process of COR synthesis in response to iron ion addition, the proteomic profiles were examined during different COR accumulation stages. These sampling points corresponded to the early phase of COR synthesis at 4 days post-inoculation (dpi), the late phase at 8 dpi, and the late stationary phase at 10 dpi in the fermentation culture. It was expected that not only COR synthesis and accumulation but also protein expression patterns would most likely be affected during fermentation by the addition of different concentrations of FeCl_3_. Principal component analysis (PCA) was performed on the control and FeCl_3_ treatment groups in fermentation culture at the three aforementioned time points (Figure 2). The first component (PC1) and second component (PC2) explained 43.07 and 21.03%, 48.72 and 17.23%, 43.29 and 20.70% of the total variation in proteins expressions at the three time points, respectively. From the diagram, we can see that replicate samples at each time point are highly similar in all cases and that protein expression patterns in response to different concentrations of FeCl_3_ are easily distinguishable from each other, indicating that there are obvious differences between the samples.

**FIGURE 3:**
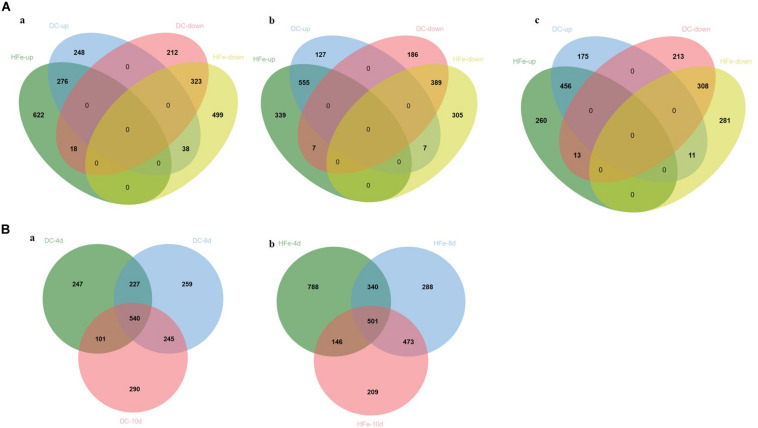
Number of common and specific significantly differentially expressed proteins in DC3000 with the addition of 20 μmol/L FeCl_3_ (DC) and 400 μmol/L FeCl_3_ (HFe) compared to the control group with no addition of FeCl_3_ (0Fe). **(A)** Venn diagrams showing upregulated and downregulated proteins between three time points, respectively, which are **(a)** 4 days after inoculation, **(b)** 8 days after inoculation, and **(c)** 10 days after inoculation. **(B)** Venn diagrams showing unique and shared upregulated and downregulated proteins between three time points, which are **(a)** 20 μmol/L FeCl_3_ (DC) compared to the control group **(b)** 400 μmol/L FeCl_3_ (HFe) compared to the control group, quantified in all three biological replicates with fold change >2 or <0.5 in quantity (*p* < 0.05). Venn analysis used the VennDiagram package (R, Version 1.6.20).

To identify the proteins whose expression significantly changed between the additions of different concentrations of FeCl_3_ to the fermentation culture, quantitative proteomic analysis was carried out via the DIA technique. A total of 3907 proteins were identified in the three groups of samples at the three time points. According to Student’s *t*-test, when the protein fold change was >2 or <0.5 and the *p*-value <0.05, as shown in Figure 3, 1115, 1271, and 1176 differentially expressed proteins were detected in the DC group compared with the 0Fe group at 4, 8, and 10 dpi, respectively, including 562, 689, and 642 upregulated proteins, respectively and 553,582 and 534 downregulated proteins, respectively. In the HFe group, 1776, 1602, and 1329 differently expressed proteins were detected at 4, 8, and 10 dpi, respectively, including 916, 901, and 729 upregulated proteins, respectively, and 860, 701, and 600 downregulated proteins, respectively. In addition, there were 747 upregulated proteins in common and 657 down-regulated proteins in common in the two groups with FeCl_3_ added, most of which occurred at 8 dpi and 10 dpi.

### Functional Classification of Proteins in *P. syringae* pv. *tomato* DC3000

#### Function Categories of Proteins Significantly Up- or Downregulated

**FIGURE 4:**
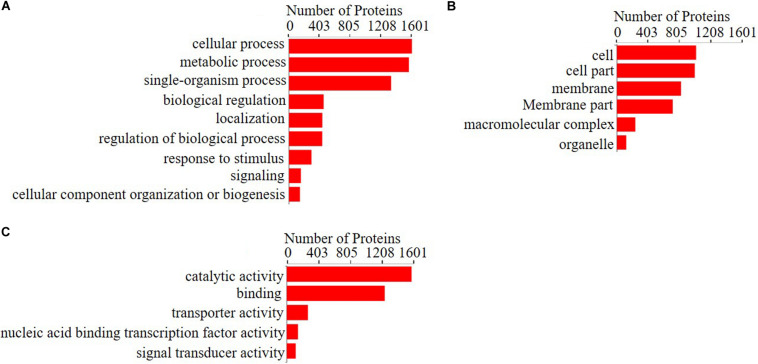
Gene ontology (GO) analysis for the proteins that changed significantly between the treatment group and the control group. Gene ontology enrichment analysis of **(A)** biological process, **(B)** cellular components, and **(C)** molecular functions for regulated proteins with fold change >2 or <0.5 with *p*-value <0.05. Blast2GO version 5 was used for functional annotation, and GOATOOLS was used to perform GO enrichment analysis.

To investigate the functional annotations of significantly changed proteins between the treatment group and the 0Fe group, Gene Ontology (GO) analysis was applied, and the regulated proteins whose fold change was >2 or <0.5 with a *p*-value <0.05 were categorized according to their function (Figure 4). The functions of the differentially expressed proteins were divided into three categories, biological processes, cellular components, and molecular functions, and included 20 subclasses: nine biological processes, six molecular functions, and five cellular components. The biological process category of the differentially expressed proteins involved mainly cellular processes, metabolic processes, biological process regulation, and signal transduction. The molecular function category involved mainly nucleic acid binding and DNA binding, catalytic reduction reactions, and transport activities. The cellular component category included mainly intracellular cell membranes, cell composition, macromolecular complexes, organic matter, etc. The specific functions of these differentially expressed proteins are associated with “energy generation and conversion,” “amino acid transport and metabolism,” “carbon transport and metabolism,” “synthesis transport and metabolism of secondary metabolites,” and “inorganic ion transport and metabolism,” as well as many other aspects. These results show that FeCl_3_ regulates the expression of proteins that actively participate in a series of physiological metabolic processes in *P. syringae* pv. *tomato* DC3000.

#### FeCl_3_ Upregulates the Expression of Proteins Related to the Key Mechanisms of Coronatine Synthesis and Regulation

**FIGURE 5:**
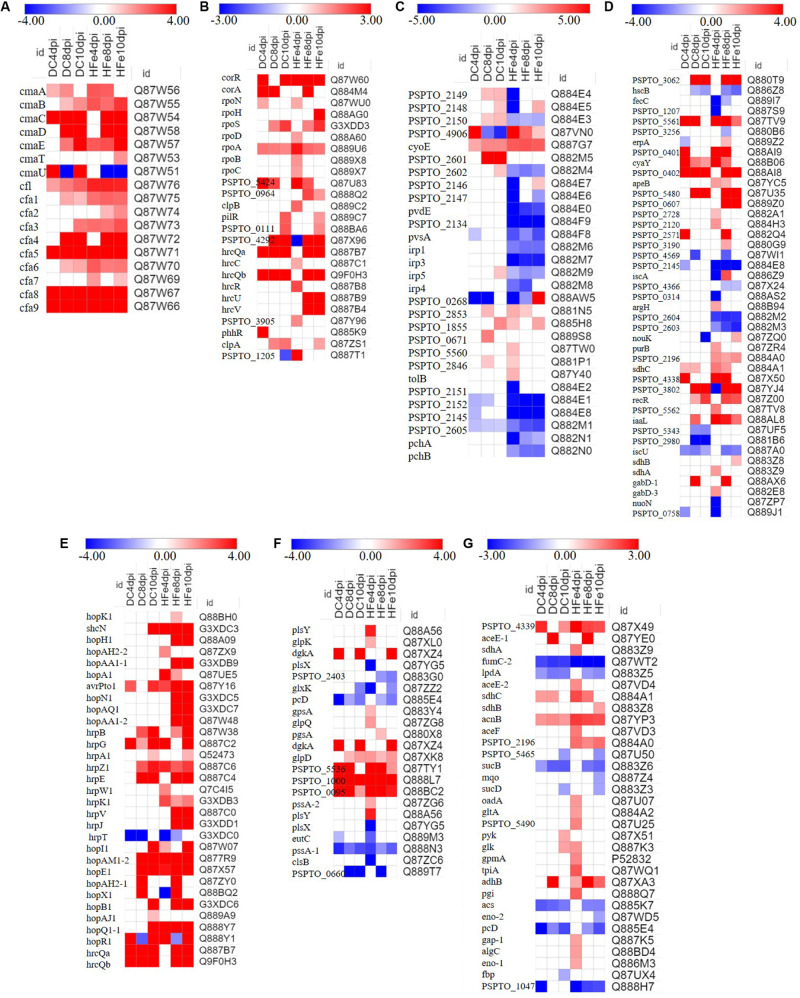
Expression pattern of major differential expression proteins in samples at three time points. **(A–G)** shows the proteins that changed significantly in the treatment group with the addition of 20 μmol/L FeCl_3_ (DC) and 400 μmol/L FeCl_3_ (HFe) compared to the control group with no addition of FeCl_3_ (0Fe), and the color bars show log2-fold change values between them, *p*-value < 0.05. Detailed data are presented in Supplementary Table S1. Heatmap analysis used Scipy (Python, Version 1.0.0).

Coronatine biosynthesis proteins, including seven proteins involved in CMA biosynthesis (cmaD, cmaE, cmaA, cmaB, cmaC, cmaT, and cmaU) and 10 proteins involved in CFA biosynthesis (cfl and cfa1-9), were significantly differentially expressed (Figure 5A). These proteins have been reported to be involved in the biosynthesis of COR, which is essential in the accumulation of COR (Gemperlein et al., 2017). In this study, the abundance of COR biosynthesis proteins increased in the FeCl_3_ treatment group, especially the proteins cmaC, cmaD, cmaE, cfa4, cfa5, cfa8, and cfa9, and their protein fold change was much greater than two. Some proteins, such as cmaB, cmaD, cmaT, cfa2, cfa4, and cfa7 were not identified as being differentially expressed at 4 dpi, suggesting that their expression quantity or fold change was too low to be detected by our data analysis methods. The obtained data proved that FeCl_3_ may upregulate the expression of COR biosynthesis proteins, suggesting that FeCl_3_ directly affects the production of COR in *P. syringae* pv. *tomato* DC3000.

The regulation of COR biosynthesis involves a complex network that is regulated by many environmental and nutrient-related factors and genes and proteins, including corRSP regulators, σ factors, and the outer membrane type III secretion protein hrcC (Palmer and Bender, 1993; Palmer et al., 1997; Fouts et al., 2002; Smirnova et al., 2002; Buell et al., 2003). In the present study, 25 proteins related to the regulation of COR were significantly differentially expressed (Figure 5B). corR is a DNA-binding response regulator that has a positive regulatory effect on the expression of CFA and CMA genes (Peñalozavázquez and Bender, 1998; Wang et al., 1999). Our results showed that the expression of corR was significantly upregulated in the treatment groups with FeCl3. Furthermore, RpoN, rpoH, rpoS, rpoD, and other proteins were RNA polymerase σ factors; in *P. syringae*, *rpoN* knockout strains were shown to be unable to produce COR (Alarcón-Chaidez et al., 2003). An increased abundance of rpoN and other related proteins (rpoH, rpoS, rpoD, rpoA, rpoB, and rpoC) were detected at 4, 8, or 10 dpi. In addition, previous work revealed that COR production of DC3000-hrcC mutants increased after 24 h, indicating that hrcC may regulate the production of COR (Peñaloza-Vázquez et al., 2000). The expression of HrcC and other regulatory proteins significantly changed to varying degrees in the present study. Therefore, on the basis of these obtained data, we speculated that FeCl_3_ may promote the expression of regulatory proteins involved in COR synthesis, which in turn affects the accumulation of COR.

#### Proteins Related to Iron Ion Absorption and Utilization

To gain more insight into the role of FeCl_3_ in the biosynthesis of COR, the expression changes of proteins related to iron ion absorption and utilization were analyzed. Two extracellular siderophores, yersiniabactin and pyoverdine, along with multiple putative TonB-dependent siderophore receptors and proteins involved in iron uptake or storage have been reported in *P. syringae* (Buell et al., 2003; Bultreys et al., 2006; Taguchi et al., 2009; Perry and Fetherston, 2011; Meneely and Lamb, 2012). In this study, the expression of these proteins significantly changed to different degrees (Figures 5C,D). An increased abundance of Irp5, PSPTO_2602, PSPTO_2149, PSPTO_2148, PSPTO_2150, PSPTO_2853, PSPTO_1855, PSPTO_0671, PSPTO_5560, and PSPTO_2846 was observed in the DC group, but, with the exceptions of PSPTO_2853 and PSPTO_1855, the expression of these proteins was downregulated in the HFe group. In addition, the abundance of Irp1, Irp3, Irp4, PSPTO_2146, PSPTO_2147, pvdE, PSPTO_2134, pvsA, PSPTO_2151, PSPTO_2152, PSPTO_2145, and PSPTO_2605 decreased only in the HFe group. The obtained results suggested that the expression of these proteins was repressed because of the high concentration of iron ions. Previous studies have shown that the expression of genes related to pyoverdine and siderophores was affected by iron concentration in the growth media (Bronstein et al., 2008; Markel et al., 2013). Furthermore, the expression of some proteins induced by iron or others whose products use iron as a co-factor also significantly changed in this study. Compared with that in the 0Fe group, the abundance of gabD-1, gabD-3, purB, and PSPTO_2196 increased; these proteins are related to the production of succinic semialdehyde, which is the precursor of the COR synthesis pathway (Rangaswamy et al., 1998).

Moreover, the expression of proteins related to the type III secretion system (TTSS) was upregulated in the treatment groups to which FeCl_3_ was added (Figure 5E). The genes encoding proteins of the TTSS were demonstrated to respond to iron ions, and their expression was induced after the addition of iron citrate (Bronstein et al., 2008). In addition, the TTSS and COR are inextricably linked; for example, both are regulated by the virulence regulator hrpRSL, and both involve jasmonate signaling pathways upon transmission to host plants. Moreover, some of the important type III conservative effector proteins are closely related to COR synthesis genes (Xiao et al., 1994; Ullrich et al., 1995; Alfano et al., 2000; Anderson et al., 2004; Tang et al., 2006; Shindo et al., 2016; Xin et al., 2016). The expressions of hopH1, hopAA1-1, hopN1, hopAQ1, hopAA1-2, hrpB, hrpA1, hrpZ1, hrpE, hrpW1, hrpK1, hrpV, and hrpJ were increased significantly, mainly at 8 dpi and 10 dpi, which corresponded to the stationary phases of the process. These results suggest that the TTSS may be associated with the synthesis and accumulation of COR in fermentation culture.

#### FeCl_3_ Regulates Proteins Related to Carbon and Energy Metabolism

To gain additional insight into COR accumulation in response to iron ions in culture, the changes in the abundance of proteins related to carbon and energy metabolism were analyzed (Figure 5F). Glycerol was used as the only carbon source to support the growth of bacterial cells in this study. Through oxidation and reduction routes, glycerol can be metabolized to pyruvate and 1, 3-propanediol. The results confirmed that the expression of glycerol kinase (glpK), glycerol-3-phosphate dehydrogenase NAD(P)^+^ (gpsA), glycerol-3-phosphate dehydrogenase (glpD), and other proteins involved in glycerol metabolism was significantly induced in response to iron ions. Furthermore, the abundance of these proteins was greatest at 4 dpi, indicating that they could improve the growth of bacterial cells at the beginning of the process and subsequently affect intracellular COR accumulation.

Moreover, the abundance of a large number of proteins involved in the glycolytic pathway and TCA cycle changed significantly (Figure 5G). The expression of one of the key enzymes involved in the glycolytic pathway enolase eno-1, which is essential for the degradation of carbohydrates via glycolysis, was obviously induced in the groups treated with FeCl_3_. In addition, the expressions of phosphomannomutase (algC), glucose-6-phosphate isomerase (pgi), and pyruvate kinase (pyk) were upregulated, suggesting an increased production of pyruvate. Previous studies have shown that pyruvate is not only the precursor of CFA but is also the raw material of L-isoleucine, the precursor of CMA (Parry et al., 1994; Rohde et al., 1998; Worley et al., 2013). These studies suggested that the increase in pyruvate would accelerate the biosynthesis of COR. In addition, the abundance of succinyl-CoA synthetase (sucD) decreased, while the abundance of pyruvate dehydrogenase (aceE1 and aceE2), citrate synthase (gltA), aconitate hydratase (acnB), and succinate dehydrogenase (sdhB and sdhC), all of which are involved in the TCA cycle, increased in the study. These obtained data showed that iron ions affect carbon and energy metabolism in fermentation culture, which consequently affects the biosynthesis of COR.

#### FeCl_3_ Regulates Proteins Related to ABC Transporters

The expression of a large number of ABC transporter proteins was differentially regulated during COR synthesis under iron ion fermentation culture (Supplementary Table S1). ABC transporters are a family of membrane proteins that mediate various ATP-driven transport processes and are among the most extensive transmembrane transport proteins in bacteria (Tanaka et al., 2018). Furthermore, ABC transporters play an important role not only in the uptake of nutrients but also in the maintenance of cell structure, the response to environmental stresses, and microcin and bacteriocin export (Beis and Rebuffat, 2019; Garcia et al., 2019). *P. syringae* pv. *tomato* DC3000 contains 15 families of ABC transporters involved in importing amino acids, sugars, and other compounds (Buell et al., 2003). The expression of ABC transporters responsible for amino acid uptake (PSPTO_4887, PSPTO_4173, PSPTO_4919, PSPTO_1258, PSPTO_1256, and PSPTO_1255), glucose ABC transporter (PSPTO_1294, PSPTO_1293, and gltK), ribose ABC transporters (rbsA-1), iron ABC transporters (PSPTO_5562 and PSPTO_5561), peptide transporters (PSPTO_4562, PSPTO_4559, dppC, PSPTO_4558, and PSPTO_4564), a mannitol ABC transporter (PSPTO_2705), dipeptide ABC transporters (PSPTO_4562, PSPTO_4559, dpc, and PSPTO_4564), a D-xylose ABC transporter (xylH), and several other proteins were upregulated in the study. The presence of these ABC transporters showed that the physiological and biochemical processes during amino acid biosynthesis, carbon metabolism and COR synthesis increased under iron ion fermentation culture conditions. Significant upregulation of the expression of the ATP-binding protein hisP, PSPTO_5273, PSPTO_5561, PSPTO_1258, PSPTO_4916, PSPTO_4551, PSPTO_3740, and PSPTO_3739 was detected during the fermentation process. The ATP-binding protein syrD of the ABC transporter was reported to play an important role in the biosynthesis and secretion of syringomycin, which is a secondary metabolite produced by *P. syringae* pv. *syringae* (Quigley and Gross, 1994). These obtained data suggested that the ABC transporter system may affect not only the uptake of nutrients for growth but also the biosynthesis of secondary metabolites, which would affect the production of COR.

### Analysis of Significantly Enriched Kyoto Encyclopedia of Genes and Genomes Pathways of Differentially Expressed Proteins

**FIGURE 6:**
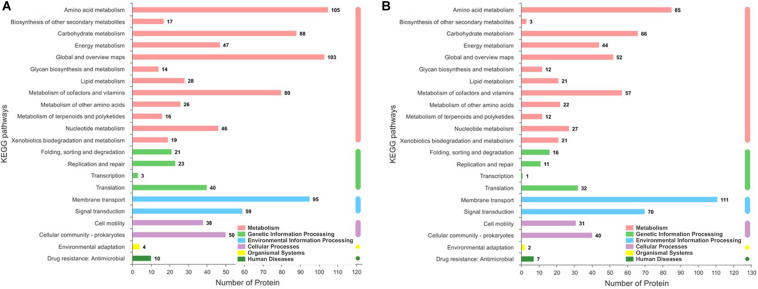
KEGG pathways showing upregulated and downregulated protein enrichment pathways between the treatment groups and the control group. **(A)** Upregulated protein enrichment pathways, **(B)** downregulated proteins enrichment pathways. Only proteins with a fold change greater than 2.0 or less than 0.5 (*p* < 0.05) were analyzed.

The significantly differentially expressed proteins were screened by the Kyoto Encyclopedia of Genes and Genomes (KEGG) database to determine the biochemical and metabolic pathways that are active in response to iron ions. A total of 115 pathways were enriched on the basis of the KEGG *P. syringae* pv. *tomato* DC3000 metabolic pathways (Figure 6). By using KEGG pathway enrichment analysis, we found that these proteins were related mainly to metabolism, genetic information processing, and environmental information processing, which included amino acid metabolism, biosynthesis of secondary metabolites, replication, transcription, translation, and signal transduction. Amino acid metabolism plays an especially important role in COR biosynthesis, as amino acids may not only provide nutrients and energy for fermentation processes but also serve as precursors of COR synthesis (i.e., isoleucine) (Gemperlein et al., 2017). In the present study, more than 200 significantly differentially expressed proteins were found to be involved in amino acid metabolism, including the biosynthesis and degradation of lysine, glycine, serine, threonine, valine, leucine, isoleucine, etc. Moreover, the analysis of the identified proteins revealed a clear enrichment of proteins involved in replication, transcription, and translation, and nearly 150 proteins involved in these biological processes were significantly changed under iron ions. Therefore, we inferred that iron ions may affect the bacterial growth and metabolism and are especially necessary for the accumulation of COR.

### Analysis and Protein–Protein Interaction Network Construction of Differentially Expressed Proteins

**FIGURE 7:**
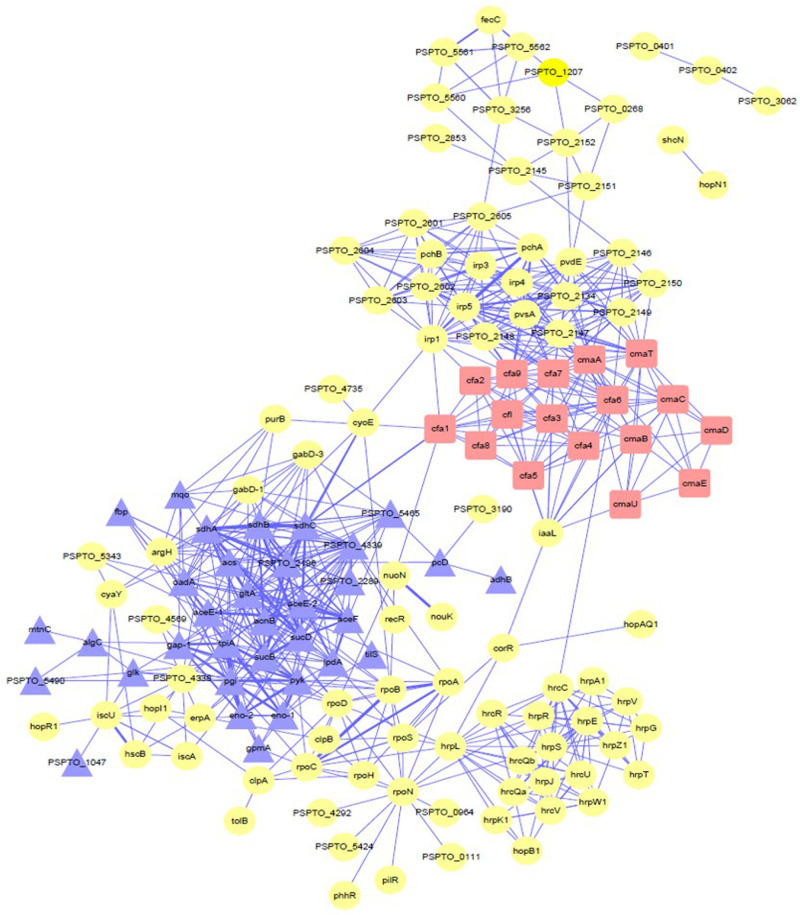
PPI network analysis showing the relationships between the differentially significant proteins. The red squares represent coronatine synthesis-related proteins. Yellow circles represent proteins associated with iron ions. Purple triangles represent proteins of the tricarboxylic acid cycle and glycolysis. Detailed data are presented in Supplementary Table S2. PPI network analysis used STRING and Cytoscape (Version 3.7.2).

To research the relationship between the significantly differentially expressed proteins and COR synthesis under the regulation of iron ions, a protein–protein interaction (PPI) network was constructed to connect these proteins (Figure 7). By using this network, we can predict the direct interactions and indirect functional relationships between known proteins. We found that many kinds of proteins in the PPI network interact with each other and may be involved in regulating different kinds of activities. Some of these proteins, including gltA, PSPTO_4339, cfa7, cfa6, cfa9, sdhA, irp5, cmaT, tpiA, irp1, pgi, irp4, sdhB, cmaA, sucD, pyk, oadA, pvdE, and cfl, were considered key node proteins because they were involved in multiple networks at the same time. Moreover, we can also speculate that, among the COR synthesis proteins, iron ion absorption and utilization proteins, glycolysis, and TCA cycle proteins, these proteins may be related to each other.

## Conclusion

**FIGURE 8:**
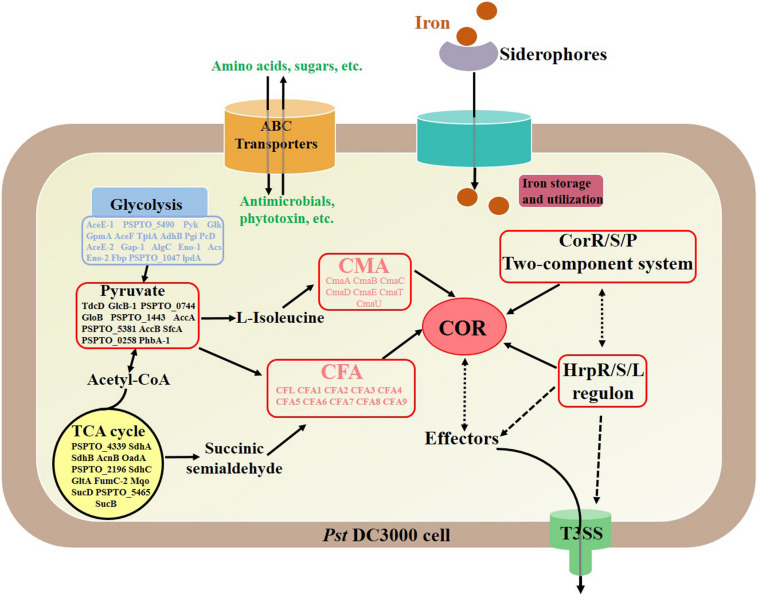
Summary scheme showing the main differentially expressed proteins during coronatine synthesis under an iron fermentation environment. Solid line, direct relationship; dotted line, unknown indirect relationship.

This study demonstrated the cellular and COR production of *P. syringae* pv. *tomato* DC3000 in response to iron ion addition at the proteomic level. The obtained proteomic profiles revealed changes in the abundance of many proteins during different fermentation phases, and COR synthesis was detected (Figure 8). The results showed that COR production increased when FeCl_3_ was added to the fermentation medium and that the expression of COR synthesis proteins was upregulated during fermentation. In addition, the expression of the majority of proteins involved in carbon and energy metabolism was also upregulated when COR was synthesized, suggesting that most of the metabolic activities were active in the bacterial cells under iron ion fermentation conditions. Moreover, there was an increase in the abundance of proteins that encode ABC transporters, which are essential for the uptake of nutrients and for responses to environmental stresses. It was also verified that iron ion addition triggered the expression of proteins related to DNA replication, transcription, and translation and influenced the biosynthesis and degradation of amino acids, which was essential for synthesizing COR precursors. Moreover, a PPI network showed the possible relationships among significantly differentially expressed proteins. The present data allowed an analysis of DIA proteomic data concerning COR synthesis in response to iron ion addition. This study expands the knowledge of the regulatory network underlying COR biosynthesis and provides a theoretical basis for fermentation production of COR.

## Data Availability Statement

The mass spectrometry proteomics data have been deposited to the ProteomeXchange Consortium (http://proteomecentral.proteomexchange.org) with the dataset identifier PXD018139.

## Author Contributions

All authors listed have made a substantial, direct and intellectual contribution to the work, and approved it for publication.

## Conflict of Interest

The authors declare that the research was conducted in the absence of any commercial or financial relationships that could be construed as a potential conflict of interest.
