# Supplementary material for: Data-Independent Acquisition Proteomics Unravels the Effects of Iron Ions on Coronatine Synthesis in Pseudomonas syringae pv. tomato DC3000
Source: Front Microbiol. 2020 Jul 21;11:1362. doi: 10.3389/fmicb.2020.01362 (PMC7385143; doi:10.3389/fmicb.2020.01362)
Supplement: Supplementary file 1 [file Table_1.DOCX]

Table S1. Identification of differentially expressed proteins at 4dpi, 8dpi and 10dpi of the Pseudomonas syringae pv. tomato DC3000 fermentation during coronatine synthesis

| Accession | Description | Gene name | Log2FC（DC/0Fe） | |  | Log2FC（HFe/0Fe） | |  | Coverage | Peptides | Unique Peptides | MW  [kDa] | calc.pl |
| --- | --- | --- | --- | --- | --- | --- | --- | --- | --- | --- | --- | --- | --- |
|  |  |  | 4dpi | 8dpi | 10dpi | 4dpi | 8dpi | 10dpi |  |  |  |  |  |
| Proteins Related to the key Mechanisms of Coronatine Synthesis and Regulation | | | | | | | |  |  |  |  |  |  |
| Q87W56 | Coronamic acid synthetase CmaA | cmaA | 1.322 | 1.918 | nd | 2.699 | 2.598 | nd | 67 | 10 | 10 | 18 | 5.39 |
| Q87W55 | Coronamic acid synthetase CmaB | cmaB | nd | 1.179 | 1.879 | 2.749 | 2.385 | 3.215 | 40 | 8 | 5 | 36.1 | 6.61 |
| Q87W54 | Coronamic acid synthetase CmaC | cmaC | 6.145 | 5.891 | 6.070 | nd | 6.372 | 6.807 | 33 | 7 | 7 | 25.7 | 9.04 |
| Q87W58 | Coronamic acid synthetase CmaD | cmaD | nd | 5.891 | 6.070 | nd | 6.372 | 6.807 | 39 | 11 | 10 | 32.2 | 8.97 |
| Q87W57 | Coronamic acid synthetase CmaE | cmaE | nd | 2.246 | 6.070 | 2.671 | 2.839 | 6.807 | 47 | 8 | 4 | 25.9 | 4.77 |
| Q87W53 | Coronamic acid synthetase, thioesterase component | cmaT | nd | nd | nd | nd | nd | 1.794 | 61 | 5 | 2 | 14.8 | 6.8 |
| Q87W51 | CmaU protein | cmaU | 6.145 | -16.610 | 6.070 | nd | -16.610 | -16.610 | 17 | 10 | 3 | 83 | 7.31 |
| Q87W76 | Coronafacic acid synthetase, ligase component | cfl | 1.625 | 2.154 | 2.612 | 3.507 | 3.543 | 3.463 | 70 | 7 | 3 | 17.5 | 5.06 |
| Q87W75 | Coronafacic acid synthetase, acyl carrier protein component | cfa1 | nd | 1.455 | 1.200 | 2.353 | 2.547 | 2.430 | 58 | 8 | 5 | 19.1 | 6.42 |
| Q87W74 | Coronafacic acid synthetase, dehydratase component | cfa2 | nd | nd | nd | nd | 1.268 | 1.919 | 51 | 9 | 5 | 20.2 | 5.07 |
| Q87W73 | Coronafacic acid beta-ketoacyl synthetase component | cfa3 | nd | nd | 2.217 | 2.384 | 2.298 | 2.823 | 70 | 7 | 1 | 15 | 4.6 |
| Q87W72 | Coronafacic acid synthetase component | cfa4 | nd | 5.891 | 6.070 | nd | 6.372 | 6.807 | 31 | 7 | 1 | 30.2 | 4.58 |
| Q87W71 | Coronafacic acid synthetase, ligase component | cfa5 | 6.145 | 5.891 | 6.070 | 7.797 | 6.372 | 6.807 | 42 | 5 | 5 | 7.8 | 9.32 |
| Q87W70 | Coronafacic acid polyketide synthase I | cfa6 | nd | 1.262 | 1.480 | 2.652 | 2.149 | 2.215 | 61 | 5 | 1 | 20.2 | 4.86 |
| Q87W69 | Coronafacic acid polyketide synthetase II | cfa7 | nd | nd | nd | 1.715 | nd | 1.145 | 66 | 7 | 1 | 16.4 | 7.2 |
| Q87W67 | Crotonyl-CoA reductase | cfa8 | 6.145 | 5.891 | 6.070 | 7.797 | 6.372 | 6.807 | 59 | 5 | 2 | 13.6 | 5.58 |
| Q87W66 | CFA synthetase, thioesterase component | cfa9 | 6.145 | 5.891 | 6.070 | 7.797 | 6.372 | 6.807 | 27 | 7 | 1 | 37.8 | 6.39 |
| Q87W60 | DNA-binding response regulator CorR | corR | 6.145 | nd | 6.070 | 7.797 | 6.372 | 6.807 | 67 | 6 | 2 | 10.6 | 9.13 |
| Q884M4 | Magnesium and cobalt transport protein, putative | corA | 6.145 | 5.891 | nd | nd | 6.372 | nd | 45 | 5 | 2 | 14.4 | 6.54 |
| Q87WU0 | RNA polymerase sigma-54 factor | rpoN | 1.037 | nd | nd | 1.017 | nd | nd | 30 | 10 | 1 | 45.1 | 5.16 |
| Q88AG0 | RNA polymerase sigma factor RpoH | rpoH | nd | nd | nd | nd | nd | 6.807 | 44 | 22 | 3 | 63.7 | 9.39 |
| G3XDD3 | RNA polymerase sigma factor RpoS | rpoS | nd | 1.486 | 2.335 | nd | 1.499 | 2.461 | 60 | 27 | 8 | 58.5 | 6.35 |
| Q88A60 | RNA polymerase sigma factor RpoD | rpoD | nd | nd | nd | 1.132 | nd | nd | 45 | 25 | 3 | 57 | 4.88 |
| Q889U6 | DNA-directed RNA polymerase subunit alpha | rpoA | 1.606 | 1.544 | 1.418 | 2.793 | 1.782 | 1.639 | 48 | 13 | 0 | 39.4 | 5.03 |
| Q889X8 | DNA-directed RNA polymerase subunit beta | rpoB | nd | nd | nd | 1.259 | nd | nd | 27 | 7 | 1 | 37.8 | 6.39 |
| Q889X7 | DNA-directed RNA polymerase subunit beta' | rpoC | nd | nd | nd | 1.138 | nd | nd | 27 | 11 | 3 | 45.1 | 5.24 |
| Q87U83 | Sigma-54 dependent transcriptional regulator | PSPTO_5424 | 6.145 | 2.212 | nd | 7.797 | 2.011 | nd | 74 | 14 | 8 | 26.9 | 5.91 |
| Q888Q2 | Sigma-54 dependent transcriptional regulator/response regulator | PSPTO_0964 | nd | nd | nd | nd | 2.188 | 1.214 | 33 | 14 | 10 | 63.7 | 5.99 |
| Q889C2 | Chaperone protein ClpB | clpB | nd | nd | nd | 1.808 | nd | nd | 74 | 9 | 3 | 13.6 | 6.06 |
| Q889C7 | Type 4 fimbriae expression regulatory protein pilR | pilR | nd | nd | 1.949 | nd | nd | 1.213 | 16 | 11 | 4 | 77.9 | 5.71 |
| Q88BA6 | Sigma-54 dependent transcriptional regulator | PSPTO_0111 | nd | nd | 1.995 | nd | nd | 1.349 | 65 | 13 | 4 | 29.9 | 5.19 |
| Q87X96 | Sigma-54 dependent transcriptional regulator/response regulator | PSPTO_4292 | nd | 5.891 | 6.070 | -16.610 | 6.372 | 6.807 | 79 | 6 | 0 | 12.7 | 4.97 |
| Q887B7 | Type III secretion protein hrcQa | hrcQa | 6.145 | 5.891 | 6.070 | nd | 6.372 | 6.807 | 47 | 12 | 2 | 41.4 | 5.08 |
| Q887C1 | Outer-membrane type III secretion protein HrcC | hrcC | nd | nd | nd | 1.017 | nd | nd | 51 | 12 | 12 | 47.4 | 6 |
| Q9F0H3 | Type III secretion protein HrcQb | hrcQb | 6.145 | 5.891 | 6.070 | nd | 6.372 | 6.807 | 50 | 8 | 1 | 12.3 | 8.4 |
| Q887B8 | Type III secretion protein HrcR | hrcR | nd | nd | nd | 2.160 | nd | nd | 44 | 13 | 13 | 33.4 | 5.63 |
| Q887B9 | Type III secretion protein HrcU | hrcU | nd | nd | nd | nd | 6.372 | 6.807 | 49 | 16 | 0 | 39 | 5.21 |
| Q887B4 | Type III secretion protein HrcV | hrcV | nd | nd | nd | nd | 6.372 | 6.807 | 47 | 16 | 8 | 43.6 | 8.72 |
| Q87Y96 | ATPase, AAA family | PSPTO_3905 | nd | nd | nd | 1.399 | nd | nd | 26 | 7 | 2 | 38.2 | 6.62 |
| Q885K9 | Phenylalanine hydroxylase transcriptional activator PhhR | phhR | 6.145 | nd | nd | nd | nd | nd | 29 | 13 | 2 | 44.5 | 6.58 |
| Q87ZS1 | ATP-dependent clp protease, ATP-binding subunit ClpA | clpA | nd | 1.455 | 1.659 | nd | nd | 1.074 | 24 | 7 | 7 | 32.8 | 5.49 |
| Q887T1 | Ribosomal subunit interface protein, putative | PSPTO_1205 | nd | nd | -2.114 | 2.957 | nd | nd | 49 | 14 | 6 | 42 | 5.44 |
| Proteins Related to Iron Ion Absorption and Utilization | | | | |  |  |  |  |  |  |  |  |  |
| Q884E4 | Pyoverdine sidechain peptide synthetase III, L-Thr-L-Ser component | PSPTO_2149 | nd | 1.296 | 1.419 | -16.610 | nd | nd | 71 | 12 | 9 | 24.2 | 4.93 |
| Q884E5 | Pyoverdine sidechain peptide synthetase II, D-Asp-L-Thr component | PSPTO_2148 | nd | nd | 1.161 | -16.610 | nd | 1.591 | 41 | 15 | 15 | 57.9 | 8.09 |
| Q884E3 | Pyoverdine sidechain peptide synthetase IV, D-Asp-L-Ser component | PSPTO_2150 | nd | 1.062 | 1.347 | -1.753 | -1.450 | -1.623 | 53 | 10 | 2 | 23.2 | 5.07 |
| Q87VN0 | Bacterioferritin, putative | PSPTO_4906 | 6.145 | -2.793 | -4.269 | 7.797 | 2.936 | 1.097 | 58 | 9 | 1 | 21.2 | 5.1 |
| Q887G7 | Protoheme IX farnesyltransferase | cyoE | 1.683 | 2.353 | 2.336 | 3.255 | 3.376 | 3.206 | 22 | 23 | 21 | 176.9 | 5.95 |
| Q882M5 | Membrane protein, putative | PSPTO_2601 | nd | 5.891 | 6.070 | nd | nd | nd | 60 | 22 | 11 | 57.8 | 4.69 |
| Q882M4 | Yersiniabactin non-ribosomal peptide synthetase | PSPTO_2602 | nd | nd | 1.134 | -2.180 | -2.628 | -2.202 | 68 | 15 | 1 | 28.6 | 5.03 |
| Q884E7 | Pyoverdine biosynthesis regulatory gene, putative | PSPTO_2146 | nd | nd | nd | -16.610 | nd | 1.132 | 31 | 17 | 3 | 84.4 | 5.02 |
| Q884E6 | Pyoverdine sidechain peptide synthetase I, epsilon-Lys module | PSPTO_2147 | nd | nd | nd | -16.610 | nd | -2.002 | 24 | 11 | 8 | 67.3 | 5.6 |
| Q884E0 | Pyoverdine ABC transporter, ATP-binding/permease protein | pvdE | nd | nd | nd | -16.610 | -3.934 | -3.569 | 36 | 9 | 6 | 37.1 | 5.44 |
| Q884F9 | Pyoverdine synthetase, thioesterase component | PSPTO_2134 | nd | nd | nd | -16.610 | -16.610 | -16.610 | 21 | 7 | 4 | 47.5 | 5.69 |
| Q884F8 | Pyoverdine chromophore synthetase | pvsA | -1.165 | nd | nd | -3.408 | -2.259 | -3.571 | 51 | 12 | 4 | 31.8 | 5.16 |
| Q882M6 | Yersiniabactin polyketide/non-ribosomal peptide synthetase | irp1 | nd | nd | nd | -2.570 | -2.865 | -2.857 | 66 | 16 | 3 | 36.1 | 6.8 |
| Q882M7 | Yersiniabactin synthetase, thiazolinyl reductase component | irp3 | nd | nd | nd | -5.711 | -4.379 | -4.444 | 74 | 17 | 16 | 34.3 | 4.83 |
| Q882M9 | Yersiniabactin synthetase, salicylate ligase component | irp5 | nd | nd | 1.158 | -1.421 | -2.166 | -1.903 | 55 | 17 | 15 | 55.2 | 5.07 |
| Q882M8 | Yersiniabactin synthetase, thioesterase component | irp4 | nd | nd | nd | -2.806 | -3.519 | nd | 60 | 20 | 7 | 39.1 | 5.15 |
| Q88AW5 | TonB system transport protein, putative | PSPTO_0268 | -16.610 | -16.610 | nd | -16.610 | -2.097 | 6.807 | 70 | 12 | 8 | 29.7 | 5.39 |
| Q881N5 | TonB-dependent receptor, putative | PSPTO_2853 | nd | 1.392 | nd | 1.607 | 1.041 | nd | 38 | 5 | 3 | 17.2 | 5.39 |
| Q885H8 | TonB-dependent receptor, putative | PSPTO_1855 | nd | nd | 2.331 | 1.215 | nd | 1.756 | 70 | 7 | 4 | 17.3 | 5.06 |
| Q889S8 | TonB-dependent receptor, putative | PSPTO_0671 | nd | 2.557 | nd | nd | nd | nd | 31 | 6 | 6 | 28.7 | 6.52 |
| Q87TW0 | TonB-dependent receptor | PSPTO_5560 | nd | nd | nd | 1.321 | nd | nd | 59 | 21 | 11 | 49.9 | 6.46 |
| Q881P1 | TonB-dependent siderophore receptor, putative | PSPTO_2846 | nd | 1.086 | nd | 1.014 | nd | nd | 19 | 4 | 1 | 31.4 | 4.69 |
| Q87Y40 | Tol-Pal system protein TolB | tolB | nd | nd | nd | 1.482 | nd | nd | 52 | 4 | 2 | 15.4 | 5.6 |
| Q884E2 | TonB-dependent siderophore receptor, putative | PSPTO_2151 | nd | nd | nd | -16.610 | nd | nd | 62 | 8 | 7 | 25.7 | 7.97 |
| Q884E1 | TonB-dependent siderophore receptor, putative | PSPTO_2152 | -1.520 | -1.234 | nd | -4.724 | -5.597 | -5.590 | 44 | 11 | 0 | 28.5 | 5.77 |
| Q884E8 | Iron-regulated membrane protein, putative | PSPTO_2145 | -1.436 | nd | nd | -16.610 | -16.610 | -5.264 | 40 | 9 | 1 | 25.9 | 5.44 |
| Q882M1 | TonB-dependent siderophore receptor, putative | PSPTO_2605 | -1.282 | -1.051 | -1.008 | -2.575 | -3.470 | -3.726 | 66 | 20 | 0 | 35.9 | 5.15 |
| Q882N1 | Isochorismate synthase | pchA | nd | nd | nd | -16.610 | -1.993 | -1.551 | 67 | 14 | 1 | 36.4 | 5 |
| Q882N0 | Isochorismate pyruvate-lyase | pchB | nd | nd | nd | -2.024 | -2.834 | -2.875 | 65 | 20 | 7 | 39.2 | 5.2 |
| Q880T9 | Iron-sulfur cluster-binding protein, rieske family | PSPTO_3062 | nd | 5.891 | 6.070 | nd | 6.372 | 6.807 | 75 | 5 | 2 | 8.6 | 6.68 |
| Q886Z8 | Co-chaperone protein HscB homolog | hscB | nd | -1.227 | -1.607 | nd | -1.874 | -2.409 | 67 | 15 | 5 | 25.3 | 5.25 |
| Q889I7 | Iron(III) dicitrate transport system, permease protein FecC | fecC | nd | nd | nd | -16.610 | -1.176 | nd | 39 | 13 | 9 | 41.7 | 4.93 |
| Q887S9 | Iron(III) dicitrate transport protein fecA | PSPTO_1207 | nd | nd | nd | -16.610 | nd | nd | 53 | 10 | 10 | 30.6 | 4.59 |
| Q87TV9 | Iron-compound ABC transporter, ATP-binding protein, putative | PSPTO_5561 | 6.145 | 5.891 | nd | 7.797 | 6.372 | 2.257 | 47 | 13 | 1 | 42.1 | 5.39 |
| Q880B6 | Iron ABC transporter, periplasmic iron-binding protein, putative | PSPTO_3256 | nd | nd | nd | nd | -1.350 | nd | 82 | 4 | 2 | 9.2 | 7.21 |
| Q889Z2 | Iron-sulfur cluster insertion protein ErpA | erpA | -1.183 | nd | nd | nd | 1.102 | nd | 21 | 9 | 5 | 69.9 | 5.78 |
| Q88AI9 | Iron-sulfur cluster-binding protein, Rieske family | PSPTO_0401 | 6.145 | nd | nd | 7.797 | 6.372 | nd | 63 | 20 | 6 | 39.6 | 7.21 |
| Q88B06 | Iron-sulfur cluster assembly protein CyaY | cyaY | 6.145 | 2.069 | 1.584 | 7.797 | 2.242 | nd | 29 | 20 | 3 | 101.4 | 9.07 |
| Q88AI8 | Iron-sulfur cluster-binding protein | PSPTO_0402 | 6.145 | 5.891 | 6.070 | nd | 6.372 | 6.807 | 50 | 16 | 3 | 38.2 | 5.12 |
| Q87YC5 | Probable M18 family aminopeptidase 2 | apeB | nd | nd | nd | 1.540 | 1.208 | nd | 44 | 5 | 4 | 17.5 | 4.61 |
| Q87U35 | Peptidase, M23/M37 family | PSPTO_5480 | nd | 5.891 | 6.070 | nd | 6.372 | 6.807 | 51 | 19 | 9 | 47.5 | 5.55 |
| Q889Z0 | Peptidase, M23/M37 family | PSPTO_0607 | nd | nd | nd | nd | 6.372 | 6.807 | 25 | 10 | 4 | 55.9 | 7.11 |
| Q882A1 | Peptidase, U7 family | PSPTO_2728 | nd | nd | nd | 1.015 | nd | nd | 26 | 3 | 1 | 15.3 | 5.36 |
| Q884H3 | Peptidase, M20/M25/M40 family | PSPTO_2120 | nd | nd | nd | 1.666 | nd | nd | 31 | 8 | 3 | 32.5 | 4.94 |
| Q882Q4 | Peptidase, M20/M25/M40 family | PSPTO_2571 | 6.145 | nd | nd | nd | 6.372 | nd | 51 | 16 | 4 | 30.6 | 4.75 |
| Q880G9 | Peptidase, M20/M25/M40 family | PSPTO_3190 | nd | nd | nd | nd | 1.375 | nd | 19 | 6 | 6 | 53.2 | 7.3 |
| Q87WI1 | Iron-uptake factor | PSPTO_4569 | nd | -2.964 | nd | nd | -2.557 | nd | 35 | 9 | 3 | 32.4 | 5.16 |
| Q884E8 | Iron-regulated membrane protein, putative | PSPTO_2145 | -1.436 | nd | nd | -16.610 | -16.610 | -5.264 | 40 | 9 | 1 | 25.9 | 5.44 |
| Q886Z9 | Iron-binding protein IscA | iscA | nd | nd | nd | -16.610 | 2.765 | nd | 31 | 17 | 5 | 77.4 | 5.07 |
| Q87X24 | Iron-regulated protein A, putative | PSPTO_4366 | nd | nd | nd | nd | -1.069 | -1.033 | 64 | 7 | 4 | 16.7 | 4.94 |
| Q88AS2 | Iron ABC transporter, periplasmic iron-binding protein | PSPTO_0314 | nd | nd | nd | -16.610 | nd | nd | 47 | 16 | 8 | 43.6 | 8.72 |
| Q88B94 | Argininosuccinate lyase | argH | nd | nd | nd | 1.424 | nd | nd | 48 | 13 | 1 | 43 | 5.08 |
| Q882M2 | ABC transporter, ATP-binding/permease protein | PSPTO_2604 | nd | nd | nd | -3.087 | -3.348 | -3.253 | 68 | 18 | 10 | 39.7 | 5.78 |
| Q882M3 | ABC transporter, ATP-binding/permease protein | PSPTO_2603 | nd | nd | nd | -1.766 | -2.982 | -3.502 | 61 | 21 | 18 | 42.8 | 5.83 |
| Q87ZQ0 | NADH-quinone oxidoreductase subunit K | nouK | nd | nd | -16.610 | nd | nd | 1.285 | 41 | 4 | 4 | 22.9 | 4.7 |
| Q87ZR4 | Adenylosuccinate lyase | purB | nd | nd | nd | 1.547 | nd | nd | 31 | 5 | 1 | 19.7 | 7.84 |
| Q884A0 | Succinate dehydrogenase hydrophobic membrane anchor subunit | PSPTO_2196 | nd | nd | nd | 1.722 | 1.406 | 1.712 | 48 | 10 | 5 | 28.7 | 5.05 |
| Q884A1 | Succinate dehydrogenase, cytochrome b556 subunit | sdhC | 1.150 | 1.190 | nd | 2.145 | 1.611 | nd | 38 | 13 | 8 | 44.2 | 8.31 |
| Q87X50 | Oxidoreductase, iron-sulfur-binding protein | PSPTO_4338 | 6.145 | nd | nd | 7.797 | 5.121 | nd | 47 | 8 | 0 | 18 | 5.85 |
| Q87YJ4 | 1-aminocyclopropane-1-carboxylate deaminase, putative | PSPTO_3802 | nd | 5.891 | 6.070 | -16.610 | 6.372 | 6.807 | 29 | 7 | 7 | 36.2 | 5.44 |
| Q87Z00 | Recombination protein RecR | recR | nd | 1.324 | 3.101 | nd | 2.949 | 2.720 | 27 | 4 | 3 | 23.5 | 6.95 |
| Q87TV8 | Iron compound ABC transporter, iron compound-binding protein | PSPTO_5562 | nd | nd | nd | 2.083 | nd | nd | 25 | 14 | 1 | 52.4 | 6.54 |
| Q88AL8 | Indoleacetate-lysine ligase | iaaL | nd | 2.515 | nd | 4.003 | 3.637 | 2.218 | 59 | 16 | 10 | 42.4 | 5.16 |
| Q87UF5 | Probable Fe(2+)-trafficking protein | PSPTO_5343 | nd | -1.819 | -1.914 | nd | nd | nd | 51 | 11 | 5 | 32.4 | 5.91 |
| Q881B6 | Oxidoreductase, 2OG-Fe(II) oxygenase family | PSPTO_2980 | nd | -16.610 | -16.610 | nd | nd | nd | 37 | 4 | 3 | 15.2 | 7.09 |
| Q887A0 | Iron-sulfur cluster assembly scaffold protein IscU | iscU | -1.855 | -2.092 | -1.397 | nd | -2.205 | -2.005 | 67 | 16 | 3 | 32.3 | 5.17 |
| Q883Z8 | Succinate dehydrogenase, iron-sulfur protein | sdhB | nd | nd | nd | nd | nd | 1.057 | 44 | 11 | 6 | 21.3 | 5.07 |
| Q883Z9 | Succinate dehydrogenase flavoprotein subunit | sdhA | nd | nd | nd | 1.565 | nd | nd | 66 | 5 | 3 | 12.9 | 5.05 |
| Q88AX6 | Succinate-semialdehyde dehydrogenase | gabD-1 | nd | 5.891 | nd | nd | 6.372 | nd | 62 | 9 | 1 | 16 | 5.92 |
| Q882E8 | Succinate-semialdehyde dehydrogenase | gabD-3 | nd | nd | nd | 1.653 | nd | nd | 72 | 17 | 17 | 42.1 | 6.09 |
| Q87ZP7 | NADH-quinone oxidoreductase subunit N | nuoN | nd | nd | nd | -16.610 | nd | nd | 17 | 4 | 1 | 33.2 | 5.33 |
| Q889J1 | Oxidoreductase, 2OG-Fe(II) oxygenase family | PSPTO_0758 | -1.751 | nd | nd | -16.610 | nd | nd | 58 | 6 | 1 | 16 | 5.07 |
| Q88BH0 | Type III effector HopK1 | hopK1 | nd | nd | nd | nd | 1.098 | nd | 62 | 8 | 7 | 25.7 | 7.97 |
| G3XDC3 | Type III chaperone protein ShcN | shcN | nd | nd | 6.070 | 7.797 | 6.372 | 6.807 | 87 | 23 | 15 | 34.5 | 6.57 |
| Q88A09 | Type III effector HopH1 | hopH1 | nd | nd | nd | nd | 6.372 | 6.807 | 80 | 23 | 1 | 29.6 | 5.29 |
| Q87ZX9 | Type III effector HopAH2-2 | hopAH2-2 | nd | nd | nd | 1.873 | nd | nd | 17 | 5 | 1 | 21 | 4.89 |
| G3XDB9 | Type III effector HopAA1-1 | hopAA1-1 | nd | nd | nd | nd | 6.372 | 6.807 | 39 | 20 | 5 | 52.3 | 6.48 |
| Q87UE5 | Type III effector HopA1 | hopA1 | nd | nd | nd | 7.797 | 1.469 | nd | 29 | 15 | 7 | 77.6 | 5.08 |
| Q87Y16 | Type III effector protein AvrPto1 | avrPto1 | 2.595 | nd | 3.317 | 2.942 | 4.259 | 4.246 | 38 | 6 | 4 | 19.8 | 5.35 |
| G3XDC5 | Type III effector HopN1 | hopN1 | nd | nd | nd | nd | 6.372 | 6.807 | 80 | 19 | 4 | 30.4 | 4.88 |
| G3XDC7 | Type III effector HopAQ1 | hopAQ1 | nd | nd | nd | nd | 6.372 | 6.807 | 66 | 19 | 9 | 39.9 | 5.27 |
| Q87W48 | Type III effector HopAA1-2 | hopAA1-2 | nd | nd | nd | nd | 6.372 | 6.807 | 31 | 9 | 6 | 46 | 6.62 |
| Q87W38 | ATP-dependent helicase HrpB | hrpB | nd | 2.728 | 6.070 | nd | 1.879 | 6.807 | 19 | 10 | 2 | 66.1 | 5.2 |
| Q887C2 | Type III secretion protein HrpG | hrpG | 6.145 | 1.434 | 6.070 | 7.797 | nd | 6.807 | 87 | 13 | 0 | 18.6 | 5.02 |
| Q52473 | Hrp pili protein HrpA | hrpA1 | nd | nd | 1.008 | nd | nd | 1.181 | 53 | 22 | 18 | 57.7 | 5.85 |
| Q887C6 | Harpin HrpZ | hrpZ1 | nd | 2.636 | 3.671 | 3.402 | 2.560 | 3.099 | 31 | 14 | 11 | 68.4 | 6.73 |
| Q887C4 | Type III secretion protein HrpE | hrpE | nd | 5.891 | 6.070 | nd | 6.372 | 6.807 | 57 | 8 | 1 | 17.4 | 5.64 |
| Q7C4I5 | Type III helper protein HrpW1 | hrpW1 | nd | nd | nd | 1.882 | nd | nd | 65 | 25 | 10 | 39.1 | 5.53 |
| G3XDB3 | Type III helper protein HrpK1 | hrpK1 | nd | nd | nd | 2.892 | 1.546 | 2.050 | 48 | 21 | 7 | 52.7 | 6.58 |
| Q887C0 | Negative regulator of hrp expression HrpV | hrpV | nd | nd | nd | nd | 6.372 | 6.807 | 32 | 12 | 2 | 52.5 | 6.8 |
| G3XDD1 | Type III secretion protein HrpJ | hrpJ | nd | nd | nd | nd | 6.372 | 6.807 | 72 | 23 | 11 | 52.1 | 5 |
| G3XDC0 | Type III secretion protein HrpT | HrpT | -16.610 | -16.610 | nd | -16.610 | -1.560 | nd | 90 | 25 | 24 | 30.6 | 5.38 |
| Q87W07 | Type III effector HopI1 | hopI1 | nd | nd | 6.070 | 1.283 | nd | 6.807 | 45 | 9 | 9 | 28.6 | 5.39 |
| Q877R9 | Type III effector HopAM1-1 | hopAM1-2 | nd | 5.891 | 6.070 | 7.797 | 6.372 | 6.807 | 44 | 26 | 0 | 67.9 | 5.02 |
| Q87X57 | Type III effector HopE1 | hopE1 | nd | 5.891 | 6.070 | 3.724 | 6.372 | 6.807 | 41 | 6 | 3 | 19.3 | 4.41 |
| Q87ZY0 | Type III effector HopAH2-1 | hopAH2-1 | nd | 5.891 | nd | nd | 6.372 | nd | 29 | 6 | 5 | 29 | 4.39 |
| Q88BQ2 | Type III effector HopX1 | HopX1 | nd | 5.891 | nd | -16.610 | 6.372 | nd | 47 | 12 | 1 | 35 | 5.17 |
| G3XDC6 | Type III effector HopB1 | hopB1 | nd | nd | 6.070 | nd | 6.372 | 6.807 | 48 | 19 | 10 | 63.9 | 6.68 |
| Q889A9 | Type III helper protein HopAJ1 | hopAJ1 | nd | nd | 1.019 | nd | nd | nd | 48 | 11 | 10 | 24.9 | 5.03 |
| Q888Y7 | Type III effector HopQ1-1 | hopQ1-1 | nd | nd | 6.070 | 7.797 | 6.372 | 6.807 | 65 | 8 | 3 | 13.5 | 4.88 |
| Q888Y1 | Type III effector HopR1 | hopR1 | 6.145 | -2.580 | 6.070 | 7.797 | -1.888 | 6.807 | 50 | 8 | 8 | 34.8 | 5.03 |
| Q887B7 | Type III secretion protein hrcQa | hrcQa | 6.145 | 5.891 | 6.070 | nd | 6.372 | 6.807 | 47 | 12 | 2 | 41.4 | 5.08 |
| Q9F0H3 | Type III secretion protein HrcQb | hrcQb | 6.145 | 5.891 | 6.070 | nd | 6.372 | 6.807 | 50 | 8 | 1 | 12.3 | 8.4 |
| Proteins Related to Carbon and Energy Metabolism | | | | |  |  |  |  |  |  |  |  |  |
| Q88A56 | Glycerol-3-phosphate acyltransferase | plsY | nd | nd | nd | 3.552 | nd | nd | 53 | 20 | 4 | 43.8 | 7.34 |
| Q87XL0 | Glycerol kinase | glpK | nd | nd | nd | 1.265 | nd | nd | 88 | 6 | 3 | 12 | 7.42 |
| Q87XZ4 | Diacylglycerol kinase | dgkA | 6.145 | nd | 6.070 | nd | nd | 6.807 | 43 | 6 | 2 | 12.7 | 5.71 |
| Q87YG5 | Phosphate acyltransferase | plsX | nd | nd | nd | -16.610 | nd | nd | 13 | 6 | 3 | 65.6 | 5.29 |
| Q883G0 | Glycerol kinase, putative | PSPTO_2403 | nd | nd | nd | nd | -1.624 | -2.033 | 57 | 8 | 1 | 17.4 | 5.64 |
| Q87ZZ2 | Glycerate kinase | glxK | nd | nd | -1.999 | -16.610 | nd | -1.777 | 28 | 4 | 2 | 19.3 | 4.65 |
| Q885E4 | Piperideine-6-carboxylate dehydrogenase | pcD | -16.610 | -1.415 | -2.164 | nd | -1.231 | -2.083 | 45 | 10 | 4 | 40 | 4.74 |
| Q883Y4 | Glycerol-3-phosphate dehydrogenase [NAD(P)+] | gpsA | nd | nd | nd | 1.131 | nd | nd | 45 | 13 | 5 | 38.2 | 7.87 |
| Q87ZG8 | Glycerophosphoryl diester phosphodiesterase | glpQ | nd | nd | nd | 1.607 | nd | nd | 13 | 8 | 8 | 108.6 | 6.19 |
| Q880X8 | CDP-diacylglycerol--glycerol-3-phosphate 3-phosphatidyltransferase | pgsA | nd | nd | nd | nd | 1.031 | nd | 92 | 4 | 4 | 6.5 | 4.49 |
| Q87XZ4 | Diacylglycerol kinase | dgkA | 6.145 | nd | 6.070 | nd | nd | 6.807 | 43 | 6 | 2 | 12.7 | 5.71 |
| Q87XK8 | Glycerol-3-phosphate dehydrogenase | glpD | nd | 1.118 | 1.593 | 1.629 | 1.066 | 1.614 | 21 | 9 | 7 | 65.6 | 5.64 |
| Q87TY1 | CDP-alcohol phosphatidyltransferase family protein | PSPTO_5536 | 6.145 | 5.891 | nd | 7.797 | 6.372 | 1.666 | 50 | 19 | 8 | 49.8 | 7.9 |
| Q888L7 | Phosphatidylcholine synthase | PSPTO_1000 | 6.145 | 5.891 | 6.070 | 7.797 | 6.372 | 6.807 | 49 | 11 | 1 | 32.3 | 6.95 |
| Q88BC2 | Phospholipase D family protein | PSPTO_0095 | 6.145 | 5.891 | 1.558 | 7.797 | 6.372 | 3.618 | 48 | 10 | 5 | 22.9 | 4.75 |
| Q87ZG6 | CDP-diacylglycerol--serine O-phosphatidyltransferase | pssA-2 | nd | nd | nd | 1.339 | nd | nd | 75 | 5 | 1 | 9 | 8.59 |
| Q88A56 | Glycerol-3-phosphate acyltransferase | plsY | nd | nd | nd | 3.552 | nd | nd | 53 | 20 | 4 | 43.8 | 7.34 |
| Q87YG5 | Phosphate acyltransferase | plsX | nd | nd | nd | -16.610 | nd | nd | 13 | 6 | 3 | 65.6 | 5.29 |
| Q889M3 | Ethanolamine ammonia-lyase light chain | eutC | -1.109 | nd | nd | -2.514 | nd | nd | 41 | 8 | 8 | 28.3 | 5.27 |
| Q888N3 | CDP-diacylglycerol--serine O-phosphatidyltransferase | pssA-1 | -3.408 | -2.199 | -2.800 | -3.099 | -1.685 | -2.561 | 30 | 8 | 1 | 37.4 | 5.58 |
| Q87ZC6 | Cardiolipin synthase B | clsB | nd | nd | nd | -16.610 | nd | nd | 37 | 8 | 8 | 31.8 | 7.88 |
| Q889T7 | Glycerophosphoryl diester phosphodiesterase, putative | PSPTO_0660 | nd | -16.610 | -16.610 | nd | -16.610 | nd | 15 | 16 | 1 | 118 | 9.14 |
| Q87X49 | Fumarate hydratase class I; Catalyzes the reversible hydration of fumarate to (S)- malate; Belongs to the class-I fumarase family | PSPTO_4339 | 2.527 | nd | 1.315 | 4.632 | 2.221 | 2.089 | 59 | 8 | 8 | 15.7 | 5.57 |
| Q87YE0 | Pyruvate dehydrogenase E1 component; Component of the pyruvate dehydrogenase (PDH) complex, that catalyzes the overall conversion of pyruvate to acetyl-CoA and CO(2) | aceE-1 | nd | 5.891 | nd | nd | 6.372 | nd | 22 | 5 | 1 | 34.8 | 6.9 |
| Q883Z9 | Succinate dehydrogenase flavoprotein subunit; Similar to SP:P10444, GB:D12485, GB:M57736, SP:P22413, PID:189650, PID:219944, and PID:219945; identified by sequence similarity; putative; see PMID:20190049 for expression data; similar to SP:P10444, GB:D12485, GB:M57736, SP:P22413, PID:189650, PID:219944, and PID:219945, identified by sequence similarity, putative; Belongs to the FAD-dependent oxidoreductase 2 family. FRD/SDH subfamily | sdhA | nd | nd | nd | 1.565 | nd | nd | 66 | 5 | 3 | 12.9 | 5.05 |
| Q87WT2 | Fumarate hydratase class II; Involved in the TCA cycle. Catalyzes the stereospecific interconversion of fumarate to L-malate; Belongs to the class-II fumarase/aspartase family. Fumarase subfamily | fumC-2 | -2.182 | -2.393 | -2.312 | -16.610 | -5.118 | -5.705 | 42 | 8 | 5 | 34.4 | 8.22 |
| Q883Z5 | Dihydrolipoyl dehydrogenase; See PMID:20190049 for expression data | lpdA | -1.211 | -1.613 | -1.555 | nd | -1.184 | -1.368 | 57 | 9 | 7 | 19.6 | 7.11 |
| Q87VD4 | Pyruvate dehydrogenase E1 component; Component of the pyruvate dehydrogenase (PDH) complex, that catalyzes the overall conversion of pyruvate to acetyl-CoA and CO(2) | aceE-2 | nd | nd | nd | 1.370 | nd | nd | 49 | 12 | 6 | 32.1 | 5.29 |
| Q884A1 | Succinate dehydrogenase, cytochrome b556 subunit; See PMID:20190049 for expression data; similar to GP:9947544, and SP:P10446; identified by sequence similarity; putative | sdhC | 1.150 | 1.190 | nd | 2.145 | 1.611 | nd | 38 | 13 | 8 | 44.2 | 8.31 |
| Q883Z8 | Succinate dehydrogenase, iron-sulfur protein; Similar to SP:P07014, GB:L05004, and PID:152957; identified by sequence similarity; putative; see PMID:20190049 for expression data; similar to SP:P07014, GB:L05004, and PID:152957, identified by sequence similarity, putative | sdhB | nd | nd | nd | nd | nd | 1.057 | 44 | 11 | 6 | 21.3 | 5.07 |
| Q87YP3 | Aconitate hydratase B; Identified by match to TIGR protein family HMM TIGR01342; see PMID:20190049 for expression data; identified by match to TIGR protein family HMM TIGR01342; Belongs to the aconitase/IPM isomerase family | acnB | 1.235 | 1.369 | 1.209 | 2.339 | 2.183 | 2.071 | 33 | 6 | 6 | 23.2 | 6.3 |
| Q87VD3 | Acetyltransferase component of pyruvate dehydrogenase complex; The pyruvate dehydrogenase complex catalyzes the overall conversion of pyruvate to acetyl-CoA and CO(2) | aceF | nd | nd | nd | 1.577 | nd | nd | 48 | 11 | 11 | 36.4 | 6.46 |
| Q884A0 | Succinate dehydrogenase hydrophobic membrane anchor subunit; Membrane-anchoring subunit of succinate dehydrogenase (SDH) | PSPTO_2196 | nd | nd | nd | 1.722 | 1.406 | 1.712 | 48 | 10 | 5 | 28.7 | 5.05 |
| Q87U50 | Identified by match to PFAM protein family HMM PF04223 | PSPTO_5465 | nd | nd | -1.201 | nd | nd | -1.292 | 36 | 14 | 12 | 73.5 | 5.34 |
| Q883Z6 | Dihydrolipoyllysine-residue succinyltransferase component of 2-oxoglutarate dehydrogenase complex; The 2-oxoglutarate dehydrogenase complex catalyzes the overall conversion of 2-oxoglutarate to succinyl-CoA and CO(2) | sucB | -1.172 | -1.913 | -1.949 | nd | -1.649 | -1.849 | 60 | 13 | 10 | 36.2 | 7.68 |
| Q887Z4 | Probable malate:quinone oxidoreductase; See PMID:20190049 for expression data | mqo | nd | nd | nd | nd | nd | -1.079 | 43 | 12 | 1 | 37.8 | 4.86 |
| Q883Z3 | Succinate--CoA ligase [ADP-forming] subunit alpha; Succinyl-CoA synthetase functions in the citric acid cycle (TCA), coupling the hydrolysis of succinyl-CoA to the synthesis of either ATP or GTP and thus represents the only step of substrate-level phosphorylation in the TCA. The alpha subunit of the enzyme binds the substrates coenzyme A and phosphate, while succinate binding and nucleotide specificity is provided by the beta subunit | sucD | nd | nd | -1.091 | nd | nd | -1.065 | 84 | 13 | 10 | 21.6 | 7.55 |
| Q87U07 | Oxaloacetate decarboxylase, alpha subunit; See PMID:20190049 for expression data | oadA | nd | nd | nd | 1.206 | nd | nd | 49 | 14 | 14 | 43.5 | 6.25 |
| Q884A2 | Citrate synthase; Similar to PIR:A33596; identified by sequence similarity; putative; Belongs to the citrate synthase family | gltA | nd | nd | nd | 1.176 | nd | nd | 38 | 9 | 7 | 40.1 | 5.14 |
| Q87U25 | Putative glucose-6-phosphate 1-epimerase; See PMID:20190049 for expression data; identified by match to PFAM protein family HMM PF01263; Belongs to the glucose-6-phosphate 1-epimerase family | PSPTO_5490 | nd | nd | nd | 1.454 | nd | nd | 40 | 19 | 1 | 53 | 6.64 |
| Q87X51 | Pyruvate kinase; Identified by match to PFAM protein family HMM PF02887; Belongs to the pyruvate kinase family | pyk | nd | nd | 1.004 | nd | nd | nd | 31 | 6 | 5 | 25.6 | 8.82 |
| Q887K3 | Glucokinase; See PMID:20190049 for expression data; Belongs to the bacterial glucokinase family | glk | nd | nd | 1.094 | 1.258 | nd | nd | 31 | 16 | 14 | 97.9 | 9.01 |
| P52832 | 2,3-bisphosphoglycerate-independent phosphoglycerate mutase; Catalyzes the interconversion of 2-phosphoglycerate and 3-phosphoglycerate. Essential for the growth and pathogenicity on the host plant; Belongs to the BPG-independent phosphoglycerate mutase family | gpmA | nd | nd | nd | 1.413 | nd | nd | 51 | 15 | 15 | 41.5 | 5.14 |
| Q87WQ1 | Triosephosphate isomerase; Involved in the gluconeogenesis. Catalyzes stereospecifically the conversion of dihydroxyacetone phosphate (DHAP) to D-glyceraldehyde-3-phosphate (G3P); Belongs to the triosephosphate isomerase family | tpiA | nd | nd | nd | 1.982 | nd | nd | 30 | 7 | 7 | 41.7 | 7.12 |
| Q87XA3 | Alcohol dehydrogenase II; See PMID:20190049 for expression data; similar to SP:P06758; identified by sequence similarity; putative | adhB | nd | 5.891 | nd | 1.014 | 6.372 | 1.841 | 43 | 5 | 2 | 17.6 | 7.66 |
| Q888Q7 | Glucose-6-phosphate isomerase; Identified by match to PFAM protein family HMM PF00342; see PMID:20190049 for expression data; identified by match to PFAM protein family HMM PF00342 | pgi | nd | nd | nd | 1.877 | nd | nd | 42 | 10 | 6 | 35.3 | 6.62 |
| Q885K7 | Acetyl-coenzyme A synthetase; Catalyzes the conversion of acetate into acetyl-CoA (AcCoA), an essential intermediate at the junction of anabolic and catabolic pathways. AcsA undergoes a two-step reaction. In the first half reaction, AcsA combines acetate with ATP to form acetyl-adenylate (AcAMP) intermediate. In the second half reaction, it can then transfer the acetyl group from AcAMP to the sulfhydryl group of CoA, forming the product AcCoA; Belongs to the ATP-dependent AMP-binding enzyme family | acs | -1.970 | -1.827 | -1.591 | nd | -1.480 | -1.503 | 24 | 11 | 5 | 73.5 | 5.01 |
| Q87WD5 | Enolase 2; Catalyzes the reversible conversion of 2- phosphoglycerate into phosphoenolpyruvate. It is essential for the degradation of carbohydrates via glycolysis | eno-2 | nd | nd | nd | nd | nd | -1.081 | 56 | 8 | 2 | 18.5 | 4.86 |
| Q885E4 | Piperideine-6-carboxylate dehydrogenase; Similar to GP:15076316, and GP:11990463; identified by sequence similarity; putative; Belongs to the aldehyde dehydrogenase family | pcD | -16.610 | -1.415 | -2.164 | nd | -1.231 | -2.083 | 45 | 10 | 4 | 40 | 4.74 |
| Q887K5 | Glyceraldehyde-3-phosphate dehydrogenase; See PMID:20190049 for expression data; Belongs to the glyceraldehyde-3-phosphate dehydrogenase family | gap-1 | nd | nd | nd | 1.101 | nd | nd | 45 | 16 | 14 | 52.1 | 5.67 |
| Q88BD4 | Phosphomannomutase/phosphoglucomutase; The phosphomannomutase activity produces a precursor for alginate polymerization. The alginate layer causes a mucoid phenotype and provides a protective barrier against host immune defenses and antibiotics. Also involved in core-LPS biosynthesis due to its phosphoglucomutase activity. Essential for biofilm production (By similarity) | algC | nd | nd | nd | 1.148 | nd | nd | 36 | 11 | 8 | 37.9 | 5.72 |
| Q886M3 | Enolase 1; Catalyzes the reversible conversion of 2- phosphoglycerate into phosphoenolpyruvate. It is essential for the degradation of carbohydrates via glycolysis | eno-1 | nd | nd | nd | 1.033 | nd | nd | 61 | 25 | 20 | 40.4 | 6.29 |
| Q87UX4 | Fructose-1,6-bisphosphatase class 1; Similar to SP:P19911; identified by sequence similarity; putative; see PMID:20190049 for expression data; similar to SP:P19911, identified by sequence similarity, putative | fbp | nd | nd | -1.003 | nd | nd | nd | 60 | 12 | 12 | 26.7 | 6.7 |
| Q888H7 | annotation not available | PSPTO_1047 | -16.610 | nd | nd | -16.610 | -2.218 | -2.117 | 27 | 10 | 4 | 38.4 | 5.5 |
| Proteins Related to ABC Transporters | | | |  |  |  |  |  |  |  |  |  |  |
| Q88AM5 | Sugar ABC transporter, periplasmic sugar-binding protein, putative | PSPTO_0364 | nd | nd | nd | 1.631 | nd | nd | 31 | 23 | 9 | 101.5 | 9.19 |
| Q882C4 | Mannitol ABC transporter, permease protein | PSPTO_2705 | nd | nd | 1.145 | 1.663 | 1.178 | nd | 68 | 26 | 3 | 59.1 | 5.52 |
| Q87VP8 | Branched-chain amino acid ABC transporter, permease protein | PSPTO_4887 | nd | 5.891 | nd | nd | nd | 6.807 | 31 | 9 | 4 | 38.3 | 5.92 |
| Q887J8 | Glucose ABC transporter, permease protein, putative | PSPTO_1294 | nd | nd | nd | nd | 1.550 | nd | 45 | 11 | 1 | 41.2 | 6.39 |
| Q887J9 | Glucose ABC transporter, permease protein, putative | PSPTO_1293 | 2.620 | 2.371 | 1.593 | 3.177 | 1.655 | nd | 41 | 16 | 3 | 52.5 | 4.96 |
| Q887J7 | Glucose ABC transporter, ATP-binding protein | gltK | nd | nd | nd | 1.052 | nd | 1.192 | 22 | 20 | 9 | 129.6 | 6.55 |
| Q881U7 | Putrescine ABC transporter, periplasmic putrescine-binding protein | PSPTO_2785 | -1.806 | -1.297 | -1.001 | -1.317 | -1.370 | -1.586 | 36 | 4 | 2 | 13 | 5.16 |
| Q888F1 | O-antigen ABC transporter, ATP-binding protein, putative | PSPTO_1075 | nd | nd | nd | 2.267 | nd | nd | 70 | 8 | 4 | 12.9 | 4.73 |
| Q880U3 | Glycine betaine ABC transporter, periplasmic glycine betaine-binding protein | PSPTO_3058 | -1.413 | -1.069 | nd | -16.610 | -1.487 | -1.154 | 27 | 6 | 3 | 29.4 | 4.78 |
| Q87XK5 | Amino acid ABC transporter, permease protein | PSPTO_4173 | 6.145 | 5.891 | 6.070 | 7.797 | 6.372 | 6.807 | 35 | 7 | 2 | 22.5 | 5 |
| Q885K5 | Arginine/ornithine ABC transporter, permease protein | PSPTO_1827 | 1.098 | nd | nd | -16.610 | nd | nd | 32 | 7 | 6 | 30.2 | 4.91 |
| Q885K4 | Arginine/ornithine ABC transporter, permease protein | PSPTO_1828 | nd | 5.891 | nd | -16.610 | nd | nd | 34 | 8 | 1 | 46.1 | 4.88 |
| Q885K6 | Arginine/ornithine ABC transporter, periplasmic arginine/ornithine-binding protein | PSPTO_1826 | nd | -1.397 | nd | -1.545 | -1.792 | -1.499 | 49 | 9 | 1 | 31.4 | 5.02 |
| Q885K2 | Histidine ABC transporter, ATP-binding protein | hisP | nd | nd | nd | nd | 1.182 | nd | 31 | 7 | 6 | 26 | 8.35 |
| Q880B6 | Iron ABC transporter, periplasmic iron-binding protein, putative | PSPTO_3256 | nd | nd | nd | nd | -1.350 | nd | 82 | 4 | 2 | 9.2 | 7.21 |
| Q880T0 | ABC transporter, ATP-binding/permease protein | PSPTO_3071 | nd | nd | nd | nd | -1.183 | -1.178 | 45 | 7 | 5 | 17.8 | 10.9 |
| Q87ZD9 | Sugar ABC transporter, periplasmic sugar-binding protein | PSPTO_3490 | nd | nd | nd | 1.907 | nd | nd | 21 | 7 | 2 | 35.6 | 5.27 |
| Q87UJ0 | Putrescine ABC transporter, periplasmic putrescine-binding protein | PSPTO_5307 | -1.211 | nd | nd | nd | -1.260 | -1.573 | 72 | 13 | 2 | 20.3 | 5.44 |
| Q87UM8 | Zinc ABC transporter, periplasmic zinc-binding protein | znuA | nd | nd | nd | 1.458 | nd | 1.284 | 23 | 21 | 7 | 126.3 | 9.38 |
| Q87UM3 | Histidine ABC transporter, ATP-binding protein, putative | PSPTO_5273 | nd | nd | nd | nd | 1.669 | nd | 37 | 14 | 1 | 51.1 | 5.08 |
| Q87VL7 | High affinity branched-chain amino acid ABC transporter, periplasmic amino acid-binding protein | PSPTO_4919 | nd | nd | nd | 1.411 | nd | nd | 38 | 11 | 8 | 36.1 | 4.58 |
| Q87ZT9 | ABC transporter, periplasmic substrate-binding protein, putative | PSPTO_3335 | nd | nd | -1.425 | nd | nd | -1.777 | 42 | 6 | 4 | 38.1 | 5.31 |
| Q87VL8 | High-affinity branched-chain amino acid ABC transporter, permease protein BraD | braD | 6.145 | 2.144 | 1.207 | 7.797 | 1.014 | nd | 32 | 11 | 11 | 55.1 | 5.8 |
| Q87VL9 | High-affinity branched-chain amino acid ABC transporter, permease protein BraE | braE | nd | nd | -1.432 | -16.610 | nd | nd | 52 | 10 | 8 | 30.6 | 8.62 |
| Q87TV8 | Iron compound ABC transporter, iron compound-binding protein | PSPTO_5562 | nd | nd | nd | 2.083 | nd | nd | 25 | 14 | 1 | 52.4 | 6.54 |
| Q87TV9 | Iron-compound ABC transporter, ATP-binding protein, putative | PSPTO_5561 | 6.145 | 5.891 | nd | 7.797 | 6.372 | 2.257 | 47 | 13 | 1 | 42.1 | 5.39 |
| Q889G2 | Phosphonate ABC transporter, permease protein, putative | PSPTO_0788 | 6.145 | 5.891 | 6.070 | nd | 6.372 | 6.807 | 44 | 9 | 1 | 37.4 | 4.89 |
| Q87TV7 | Hemin ABC transporter, permease protein, putative | PSPTO_5563 | 6.145 | nd | -16.610 | nd | nd | nd | 71 | 20 | 6 | 37.1 | 9 |
| Q887N3 | Amino acid ABC transporter, ATP-binding protein | PSPTO_1258 | nd | nd | nd | 1.029 | nd | nd | 26 | 11 | 4 | 73.3 | 5.68 |
| Q887N5 | Amino acid ABC transporter, permease protein | PSPTO_1256 | 2.547 | 2.289 | 1.935 | 3.838 | 2.311 | 2.116 | 47 | 11 | 3 | 36.2 | 5.02 |
| Q887N6 | Amino acid ABC transporter, periplasmic amino acid-binding protein | PSPTO_1255 | 1.181 | nd | 1.097 | 1.313 | nd | nd | 48 | 11 | 3 | 27 | 4.49 |
| Q87UN5 | D-methionine ABC transporter, permease protein | metI-2 | -2.710 | -1.698 | nd | nd | -1.477 | nd | 36 | 14 | 1 | 51.2 | 5.05 |
| Q87UN1 | Zinc ABC transporter, permease protein | znuB | 6.145 | 5.891 | 6.070 | nd | 6.372 | 6.807 | 41 | 7 | 7 | 27.4 | 6.64 |
| Q87WI4 | Dipeptide ABC transporter, permease protein | PSPTO_4562 | nd | nd | nd | 3.154 | nd | nd | 35 | 6 | 2 | 17.5 | 4.64 |
| Q87WI5 | Dipeptide ABC transporter, periplasmic dipeptide-binding protein | dppA | nd | -1.391 | nd | nd | -1.825 | nd | 22 | 11 | 11 | 70.3 | 6.07 |
| Q87WI7 | Dipeptide ABC transporter, periplasmic dipeptide-binding protein | PSPTO_4559 | nd | nd | nd | 1.511 | nd | nd | 38 | 8 | 2 | 27.4 | 5.94 |
| Q884E0 | Pyoverdine ABC transporter, ATP-binding/permease protein | pvdE | nd | nd | nd | -16.610 | -3.934 | -3.569 | 36 | 9 | 6 | 37.1 | 5.44 |
| Q87VM0 | High affinity branched-chain amino acid ABC transporter, ATP-binding protein | PSPTO_4916 | 2.430 | 2.086 | 2.680 | nd | 1.674 | 1.990 | 44 | 7 | 6 | 29.6 | 7.06 |
| Q88AC9 | Glycine/betaine/L-proline ABC transporter, permease protein | PSPTO_0463 | nd | nd | nd | -16.610 | nd | nd | 55 | 17 | 15 | 55.2 | 5.07 |
| Q87UI2 | Aliphatic sulfonates ABC transporter, permease protein | ssuC | nd | -1.429 | -1.767 | -16.610 | -16.610 | -16.610 | 35 | 17 | 16 | 52.7 | 9.07 |
| Q87UI1 | Sulfonate ABC transporter, periplasmic sulfonate-binding protein, putative | PSPTO_5316 | -1.014 | -1.964 | -2.538 | -16.610 | -3.802 | -4.121 | 24 | 20 | 18 | 116.4 | 4.88 |
| Q87WJ5 | ABC transporter, ATP-binding protein | PSPTO_4551 | nd | nd | nd | 1.311 | nd | nd | 39 | 10 | 10 | 31.8 | 6.64 |
| Q87YQ6 | ABC transporter, permease protein | PSPTO_3738 | 6.145 | 5.891 | 6.070 | 7.797 | 6.372 | 6.807 | 41 | 7 | 6 | 26.9 | 4.91 |
| Q87YQ7 | ABC transporter, permease protein | PSPTO_3737 | nd | 1.316 | 1.244 | 1.734 | 1.275 | 1.387 | 22 | 6 | 2 | 28.1 | 4.92 |
| Q87YQ4 | ABC transporter, ATP-binding protein | PSPTO_3740 | 1.158 | 1.718 | 1.843 | -16.610 | 2.010 | 2.527 | 52 | 7 | 6 | 22.8 | 5.66 |
| Q87YQ5 | ABC transporter, ATP-binding protein | PSPTO_3739 | nd | nd | nd | 2.007 | 1.205 | 1.092 | 31 | 8 | 4 | 35.5 | 4.82 |
| Q87YQ9 | ABC transporter, periplasmic substrate-binding protein, putative | PSPTO_3735 | nd | nd | nd | 1.848 | 1.299 | 1.283 | 48 | 4 | 1 | 13.8 | 4.75 |
| Q886H9 | ABC transporter, periplasmic substrate-binding protein, putative | PSPTO_1600 | nd | nd | -1.005 | nd | nd | -1.005 | 45 | 24 | 1 | 56.9 | 4.91 |
| Q87UW1 | Cystine ABC transporter, permease protein, putative | PSPTO_5181 | 6.145 | 5.891 | 6.070 | nd | 6.372 | 6.807 | 44 | 10 | 10 | 30.1 | 5.91 |
| Q880X0 | Peptide ABC transporter, permease protein | PSPTO_3030 | nd | nd | -1.290 | -16.610 | nd | nd | 25 | 5 | 3 | 29.5 | 8.75 |
| Q887K0 | Glucose ABC transporter, periplasmic glucose-binding protein, putative | PSPTO_1292 | nd | nd | -1.006 | nd | nd | nd | 40 | 10 | 8 | 35 | 5.38 |
| Q87WI3 | Dipeptide ABC transporter, permease protein | dppC | 6.145 | 5.891 | 6.070 | nd | 6.372 | 6.807 | 77 | 7 | 2 | 10.5 | 8.13 |
| Q87UH8 | Taurine ABC transporter, periplasmic taurine-binding protein | tauA | -1.000 | -1.714 | -1.792 | -16.610 | -3.319 | -3.082 | 86 | 13 | 9 | 22.9 | 4.93 |
| Q87WI8 | Dipeptide ABC transporter, periplasmic dipeptide-binding protein, putative | PSPTO_4558 | -1.906 | -2.148 | -2.212 | nd | -1.518 | -1.188 | 33 | 8 | 3 | 33.3 | 6.07 |
| Q87WI2 | Dipeptide ABC transporter, ATP-binding protein | PSPTO_4564 | nd | -16.610 | nd | 7.797 | nd | 6.807 | 19 | 12 | 6 | 93.4 | 6.99 |
| Q882I7 | L-arabinose ABC transporter, permease protein | araH | 6.145 | 5.891 | 6.070 | 7.797 | nd | 6.807 | 53 | 22 | 18 | 57.7 | 5.85 |
| Q87WH2 | Glycine betaine/carnitine/choline ABC transporter, permease protein | PSPTO_4578 | 3.102 | nd | nd | -16.610 | nd | nd | 34 | 4 | 1 | 18.2 | 5.31 |
| Q87WH4 | Glycine betaine/choline OpuC ABC transporter, permease protein | PSPTO_4576 | nd | nd | nd | -16.610 | -1.050 | nd | 48 | 10 | 0 | 28 | 4.98 |
| Q87YS4 | ABC transporter, permease protein | PSPTO_3718 | nd | nd | nd | 1.217 | nd | nd | 56 | 3 | 1 | 11.6 | 5.43 |
| Q87YS6 | ABC transporter, periplasmic substrate-binding protein | PSPTO_3716 | -1.014 | nd | nd | nd | -1.080 | nd | 29 | 7 | 5 | 37.5 | 5.08 |
| Q883E1 | ABC transporter, periplasmic substrate-binding protein, aliphatic sulfonates family | PSPTO_2423 | -2.227 | -3.903 | -2.719 | -16.610 | -16.610 | -16.610 | 74 | 14 | 8 | 26.9 | 5.91 |
| Q883E7 | Branched-chain amino acid ABC transporter, permease protein | PSPTO_2417 | nd | 5.891 | nd | nd | nd | 6.807 | 62 | 18 | 2 | 40.4 | 6.32 |
| Q883E6 | Branched-chain amino acid ABC transporter, periplasmic amino acid-binding protein | PSPTO_2418 | nd | nd | -16.610 | nd | nd | nd | 53 | 12 | 9 | 27.9 | 7.84 |
| Q883E9 | Branched-chain amino acid ABC transporter, ATP-binding protein | PSPTO_2415 | nd | nd | -16.610 | nd | nd | -16.610 | 47 | 16 | 8 | 43.6 | 8.72 |
| Q884F4 | Cation ABC transporter, permease protein | PSPTO_2139 | 1.804 | 1.587 | 1.477 | -16.610 | -16.610 | -16.610 | 44 | 9 | 9 | 37.1 | 5.22 |
| Q884F3 | Cation ABC transporter, ATP-binding protein | PSPTO_2140 | nd | nd | nd | -16.610 | -3.889 | -16.610 | 32 | 15 | 8 | 79.6 | 6.48 |
| Q884F2 | Cation ABC transporter, periplasmic cation-binding protein | PSPTO_2141 | -2.130 | -3.049 | -1.198 | -16.610 | -16.610 | -4.342 | 61 | 12 | 9 | 31.6 | 6.29 |
| Q88AS2 | Iron ABC transporter, periplasmic iron-binding protein | PSPTO_0314 | nd | nd | nd | -16.610 | nd | nd | 47 | 16 | 8 | 43.6 | 8.72 |
| Q880Z1 | D-xylose ABC transporter, permease protein | xylH | 6.145 | 2.506 | 1.661 | 7.797 | 3.211 | nd | 10 | 4 | 1 | 83.3 | 7.52 |
| Q885P0 | ABC transporter, periplasmic substrate-binding protein, aliphatic sulfonates family | PSPTO_1791 | nd | nd | nd | -16.610 | -2.148 | -2.843 | 61 | 8 | 6 | 17.4 | 5.26 |
| Q87UJ5 | Putrescine ABC transporter, permease protein | potI | nd | nd | -1.114 | -16.610 | nd | nd | 66 | 17 | 4 | 34 | 4.93 |
| Q87UJ4 | Putrescine ABC transporter, permease protein | potH | -16.610 | -16.610 | -16.610 | -16.610 | nd | nd | 58 | 10 | 8 | 27.1 | 5.03 |
| Q87WU6 | Toluene tolerance ABC transporter, ATP-binding protein, putative | PSPTO_4447 | nd | nd | nd | nd | nd | 1.046 | 46 | 10 | 9 | 36.2 | 7.17 |
| Q87XU7 | Branched-chain amino acid ABC transporter, periplasmic amino acid-binding protein | PSPTO_4078 | 6.145 | 5.891 | nd | nd | nd | 6.807 | 30 | 10 | 3 | 50.1 | 9.03 |
| Q883I9 | Ribose ABC transporter, ATP-binding protein | rbsA-1 | nd | nd | nd | 1.138 | nd | nd | 25 | 14 | 1 | 62.6 | 7.78 |
| Q87XR6 | High-affinity branched-chain amino acid ABC transporter, permease protein | PSPTO_4110 | 1.387 | 1.629 | 1.092 | 2.546 | 2.077 | 1.746 | 69 | 5 | 5 | 10.1 | 4.74 |
| Q87XR8 | High-affinity branched-chain amino acid ABC transporter, periplasmic amino acid-binding protein | PSPTO_4108 | nd | nd | nd | 1.450 | nd | nd | 68 | 6 | 6 | 15.6 | 4.46 |
| Q88B53 | Glycine/betaine family, ABC transporter, substrate-binding protein | PSPTO_0166 | nd | nd | nd | nd | 2.464 | 2.207 | 50 | 10 | 9 | 21.8 | 5.2 |
| Q883J0 | Ribose ABC transporter, periplasmic ribose-binding protein | rbsB-1 | -1.063 | nd | nd | nd | -1.430 | -1.604 | 53 | 13 | 3 | 33.9 | 7.81 |
| Q87Z12 | Sulfate ABC transporter, periplasmic sulfate-binding protein | PSPTO_3625 | nd | nd | nd | nd | -1.303 | -1.397 | 73 | 5 | 4 | 13.5 | 4.79 |
| Q87XP0 | Amino acid ABC transporter, periplasmic amino acid-binding protein | PSPTO_4136 | nd | nd | nd | 1.006 | -1.395 | -1.463 | 47 | 4 | 2 | 9.3 | 4.16 |
| Q88AW9 | Peptide ABC transporter, ATP-binding protein | PSPTO_0264 | nd | 5.891 | nd | -16.610 | nd | nd | 63 | 11 | 1 | 17.4 | 8.27 |
| Q87UV3 | D-methionine ABC transporter, permease protein | metI-1 | nd | -1.624 | nd | -16.610 | -16.610 | -16.610 | 58 | 9 | 2 | 14.7 | 5.71 |
| Q881B0 | Branched-chain amino acid ABC transporter, ATP-binding protein | PSPTO_2986 | nd | nd | -16.610 | nd | nd | -16.610 | 25 | 5 | 4 | 23.4 | 5.19 |

nd-not detected as significantly differentially expressed
