# Supplementary material for: Data-Independent Acquisition Proteomics Unravels the Effects of Iron Ions on Coronatine Synthesis in Pseudomonas syringae pv. tomato DC3000
Source: Front Microbiol. 2020 Jul 21;11:1362. doi: 10.3389/fmicb.2020.01362 (PMC7385143; doi:10.3389/fmicb.2020.01362)
Supplement: Supplementary file 2 [file Table_2.DOCX]

Table S2. Detailed information of differentially expressed proteins in PPI network

| 0 | node2 | node1_string_internal_id | node2_string_internal_id | node1_external_id | node2_external_id | neighborhood_on_chromosome | gene_fusion | phylogenetic_cooccurrence | homology | coexpression | experimentally_determined_interaction | database_annotated | automated_textmining | combined_score |
| --- | --- | --- | --- | --- | --- | --- | --- | --- | --- | --- | --- | --- | --- | --- |
| rpoA | rpoB | 11124831 | 11124799 | 223283.PSPTO_0651 | 223283.PSPTO_0619 | 0.276 | 0 | 0.777 | 0 | 0.841 | 0.899 | 0.8 | 0.739 | 0.999 |
| PSPTO_2196 | sdhC | 11126348 | 11126347 | 223283.PSPTO_2196 | 223283.PSPTO_2195 | 0.891 | 0 | 0.73 | 0 | 0.971 | 0.896 | 0.8 | 0.563 | 0.999 |
| aceF | aceE-2 | 11129065 | 11129064 | 223283.PSPTO_5006 | 223283.PSPTO_5005 | 0.559 | 0.011 | 0.639 | 0 | 0.846 | 0.51 | 0.9 | 0.459 | 0.999 |
| sdhA | sdhC | 11126349 | 11126347 | 223283.PSPTO_2197 | 223283.PSPTO_2195 | 0.891 | 0 | 0.471 | 0 | 0.853 | 0.696 | 0.8 | 0.501 | 0.999 |
| sdhB | sdhA | 11126350 | 11126349 | 223283.PSPTO_2198 | 223283.PSPTO_2197 | 0.875 | 0 | 0.784 | 0 | 0.908 | 0.673 | 0.8 | 0.557 | 0.999 |
| sdhA | PSPTO_2196 | 11126349 | 11126348 | 223283.PSPTO_2197 | 223283.PSPTO_2196 | 0.891 | 0.264 | 0.354 | 0 | 0.805 | 0.805 | 0.8 | 0.338 | 0.999 |
| rpoA | rpoC | 11124831 | 11124800 | 223283.PSPTO_0651 | 223283.PSPTO_0620 | 0.305 | 0 | 0.765 | 0 | 0.648 | 0.9 | 0.8 | 0.603 | 0.999 |
| sdhB | sdhC | 11126350 | 11126347 | 223283.PSPTO_2198 | 223283.PSPTO_2195 | 0.875 | 0 | 0.522 | 0 | 0.857 | 0.534 | 0.8 | 0.461 | 0.999 |
| rpoC | rpoB | 11124800 | 11124799 | 223283.PSPTO_0620 | 223283.PSPTO_0619 | 0.754 | 0.898 | 0.782 | 0 | 0.92 | 0.9 | 0.8 | 0.603 | 0.999 |
| hrcQa | hrcQb | 11125555 | 11125554 | 223283.PSPTO_1397 | 223283.PSPTO_1396 | 0.845 | 0.654 | 0.784 | 0 | 0 | 0 | 0.8 | 0.375 | 0.998 |
| lpdA | sucB | 11126353 | 11126352 | 223283.PSPTO_2201 | 223283.PSPTO_2200 | 0.651 | 0.124 | 0.731 | 0 | 0.334 | 0.435 | 0.9 | 0.558 | 0.998 |
| aceF | aceE-1 | 11129065 | 11127959 | 223283.PSPTO_5006 | 223283.PSPTO_3860 | 0.125 | 0.011 | 0.634 | 0 | 0.846 | 0.51 | 0.9 | 0.459 | 0.998 |
| sucD | sucB | 11126355 | 11126352 | 223283.PSPTO_2203 | 223283.PSPTO_2200 | 0.524 | 0.006 | 0.238 | 0 | 0.94 | 0.093 | 0.9 | 0.582 | 0.998 |
| sdhB | PSPTO_2196 | 11126350 | 11126348 | 223283.PSPTO_2198 | 223283.PSPTO_2196 | 0.875 | 0 | 0.349 | 0 | 0.605 | 0.532 | 0.8 | 0.371 | 0.997 |
| sucD | sdhB | 11126355 | 11126350 | 223283.PSPTO_2203 | 223283.PSPTO_2198 | 0.451 | 0 | 0.57 | 0 | 0.82 | 0.144 | 0.9 | 0.457 | 0.997 |
| aceF | lpdA | 11129065 | 11126353 | 223283.PSPTO_5006 | 223283.PSPTO_2201 | 0.352 | 0.093 | 0.546 | 0 | 0.642 | 0.49 | 0.9 | 0.528 | 0.997 |
| nuoN | nouK | 11127487 | 11127484 | 223283.PSPTO_3377 | 223283.PSPTO_3374 | 0.801 | 0 | 0.783 | 0 | 0.821 | 0.531 | 0 | 0.457 | 0.997 |
| hscB | iscU | 11125584 | 11125582 | 223283.PSPTO_1426 | 223283.PSPTO_1424 | 0.784 | 0 | 0.769 | 0 | 0.706 | 0.519 | 0 | 0.594 | 0.996 |
| tpiA | gap-1 | 11128574 | 11125447 | 223283.PSPTO_4494 | 223283.PSPTO_1287 | 0.116 | 0.18 | 0.709 | 0 | 0.647 | 0.408 | 0.9 | 0.459 | 0.996 |
| sucD | sdhA | 11126355 | 11126349 | 223283.PSPTO_2203 | 223283.PSPTO_2197 | 0.449 | 0 | 0.602 | 0 | 0.696 | 0.316 | 0.9 | 0.457 | 0.996 |
| PSPTO_5562 | PSPTO_5561 | 11129610 | 11129609 | 223283.PSPTO_5562 | 223283.PSPTO_5561 | 0.852 | 0.011 | 0.742 | 0 | 0.381 | 0.258 | 0.8 | 0.104 | 0.996 |
| pchB | pchA | 11126735 | 11126734 | 223283.PSPTO_2596 | 223283.PSPTO_2595 | 0.868 | 0 | 0.233 | 0 | 0.52 | 0 | 0.9 | 0.403 | 0.996 |
| irp5 | pchA | 11126736 | 11126734 | 223283.PSPTO_2597 | 223283.PSPTO_2595 | 0.861 | 0.347 | 0.314 | 0 | 0.871 | 0.127 | 0 | 0.457 | 0.995 |
| tpiA | pgi | 11128574 | 11125130 | 223283.PSPTO_4494 | 223283.PSPTO_0959 | 0.086 | 0 | 0.252 | 0 | 0.857 | 0.139 | 0.8 | 0.739 | 0.994 |
| rpoB | rpoD | 11124799 | 11124718 | 223283.PSPTO_0619 | 223283.PSPTO_0537 | 0.056 | 0 | 0.754 | 0 | 0.076 | 0.898 | 0 | 0.74 | 0.993 |
| hrcR | hrcU | 11125553 | 11125550 | 223283.PSPTO_1395 | 223283.PSPTO_1392 | 0.891 | 0.147 | 0.784 | 0 | 0.255 | 0 | 0 | 0.589 | 0.992 |
| sucD | sdhC | 11126355 | 11126347 | 223283.PSPTO_2203 | 223283.PSPTO_2195 | 0.449 | 0 | 0 | 0 | 0.72 | 0 | 0.9 | 0.537 | 0.991 |
| acnB | gltA | 11127851 | 11126346 | 223283.PSPTO_3752 | 223283.PSPTO_2194 | 0.338 | 0 | 0 | 0 | 0.602 | 0 | 0.9 | 0.712 | 0.991 |
| irp1 | irp5 | 11126739 | 11126736 | 223283.PSPTO_2600 | 223283.PSPTO_2597 | 0.892 | 0.006 | 0.688 | 0 | 0.545 | 0 | 0 | 0.46 | 0.99 |
| PSPTO_4339 | sdhA | 11128421 | 11126349 | 223283.PSPTO_4339 | 223283.PSPTO_2197 | 0.407 | 0 | 0 | 0 | 0.524 | 0 | 0.9 | 0.653 | 0.988 |
| pyk | eno-1 | 11128419 | 11125711 | 223283.PSPTO_4337 | 223283.PSPTO_1554 | 0.116 | 0 | 0.26 | 0 | 0.704 | 0.12 | 0.9 | 0.461 | 0.988 |
| rpoA | rpoD | 11124831 | 11124718 | 223283.PSPTO_0651 | 223283.PSPTO_0537 | 0.043 | 0 | 0.748 | 0 | 0.059 | 0.896 | 0 | 0.561 | 0.987 |
| irp4 | irp5 | 11126737 | 11126736 | 223283.PSPTO_2598 | 223283.PSPTO_2597 | 0.838 | 0.002 | 0.727 | 0 | 0.52 | 0 | 0 | 0.458 | 0.987 |
| rpoC | rpoD | 11124800 | 11124718 | 223283.PSPTO_0620 | 223283.PSPTO_0537 | 0.056 | 0 | 0.737 | 0 | 0.084 | 0.898 | 0 | 0.462 | 0.985 |
| PSPTO_4339 | sdhC | 11128421 | 11126347 | 223283.PSPTO_4339 | 223283.PSPTO_2195 | 0.407 | 0 | 0 | 0 | 0.381 | 0 | 0.9 | 0.654 | 0.985 |
| pyk | pgi | 11128419 | 11125130 | 223283.PSPTO_4337 | 223283.PSPTO_0959 | 0.186 | 0 | 0.151 | 0 | 0.822 | 0 | 0.8 | 0.459 | 0.984 |
| PSPTO_4339 | sdhB | 11128421 | 11126350 | 223283.PSPTO_4339 | 223283.PSPTO_2198 | 0.407 | 0 | 0 | 0 | 0.35 | 0 | 0.9 | 0.65 | 0.984 |
| hrcV | hrcU | 11125560 | 11125550 | 223283.PSPTO_1402 | 223283.PSPTO_1392 | 0.72 | 0.006 | 0.784 | 0 | 0.454 | 0 | 0 | 0.58 | 0.984 |
| sucD | PSPTO_2196 | 11126355 | 11126348 | 223283.PSPTO_2203 | 223283.PSPTO_2196 | 0.449 | 0 | 0 | 0 | 0.497 | 0 | 0.9 | 0.461 | 0.983 |
| irp1 | irp4 | 11126739 | 11126737 | 223283.PSPTO_2600 | 223283.PSPTO_2598 | 0.888 | 0.003 | 0.765 | 0 | 0.155 | 0 | 0 | 0.35 | 0.983 |
| eno-2 | pyk | 11128692 | 11128419 | 223283.PSPTO_4616 | 223283.PSPTO_4337 | 0.116 | 0 | 0.25 | 0 | 0.704 | 0.12 | 0.9 | 0.188 | 0.982 |
| irp1 | irp3 | 11126739 | 11126738 | 223283.PSPTO_2600 | 223283.PSPTO_2599 | 0.9 | 0 | 0.78 | 0 | 0.091 | 0 | 0 | 0.209 | 0.982 |
| PSPTO_2289 | gltA | 11126439 | 11126346 | 223283.PSPTO_2289 | 223283.PSPTO_2194 | 0.079 | 0 | 0.386 | 0 | 0.449 | 0.232 | 0.9 | 0.341 | 0.98 |
| hrcV | hrcR | 11125560 | 11125553 | 223283.PSPTO_1402 | 223283.PSPTO_1395 | 0.731 | 0 | 0.784 | 0 | 0.267 | 0 | 0 | 0.583 | 0.979 |
| PSPTO_5561 | fecC | 11129609 | 11124942 | 223283.PSPTO_5561 | 223283.PSPTO_0762 | 0.083 | 0.055 | 0.631 | 0 | 0.551 | 0.398 | 0.8 | 0.083 | 0.979 |
| sucD | lpdA | 11126355 | 11126353 | 223283.PSPTO_2203 | 223283.PSPTO_2201 | 0.634 | 0 | 0.202 | 0 | 0.263 | 0 | 0.9 | 0.2 | 0.979 |
| irp3 | irp5 | 11126738 | 11126736 | 223283.PSPTO_2599 | 223283.PSPTO_2597 | 0.882 | 0 | 0.655 | 0 | 0.141 | 0 | 0 | 0.462 | 0.978 |
| eno-1 | pgi | 11125711 | 11125130 | 223283.PSPTO_1554 | 223283.PSPTO_0959 | 0.113 | 0 | 0 | 0 | 0.705 | 0.125 | 0.8 | 0.574 | 0.976 |
| cmaT | cmaA | 11128781 | 11128778 | 223283.PSPTO_4712 | 223283.PSPTO_4709 | 0.829 | 0.017 | 0.76 | 0 | 0.114 | 0 | 0 | 0.391 | 0.975 |
| PSPTO_2602 | irp4 | 11126741 | 11126737 | 223283.PSPTO_2602 | 223283.PSPTO_2598 | 0.847 | 0 | 0.781 | 0 | 0.155 | 0 | 0 | 0.068 | 0.97 |
| irp3 | irp4 | 11126738 | 11126737 | 223283.PSPTO_2599 | 223283.PSPTO_2598 | 0.858 | 0 | 0.715 | 0 | 0.068 | 0 | 0 | 0.271 | 0.969 |
| PSPTO_2602 | irp3 | 11126741 | 11126738 | 223283.PSPTO_2602 | 223283.PSPTO_2599 | 0.847 | 0 | 0.78 | 0 | 0.091 | 0 | 0 | 0.078 | 0.968 |
| cmaC | cmaB | 11128780 | 11128779 | 223283.PSPTO_4711 | 223283.PSPTO_4710 | 0.837 | 0 | 0.711 | 0 | 0 | 0 | 0 | 0.375 | 0.968 |
| cfa3 | cfa2 | 11128756 | 11128755 | 223283.PSPTO_4683 | 223283.PSPTO_4682 | 0.847 | 0 | 0.652 | 0 | 0.116 | 0.366 | 0 | 0.077 | 0.967 |
| hrpJ | hrcQb | 11125561 | 11125554 | 223283.PSPTO_1403 | 223283.PSPTO_1396 | 0.76 | 0 | 0.783 | 0 | 0 | 0 | 0 | 0.396 | 0.966 |
| gap-1 | pgi | 11125447 | 11125130 | 223283.PSPTO_1287 | 223283.PSPTO_0959 | 0.116 | 0 | 0 | 0 | 0.607 | 0 | 0.8 | 0.546 | 0.964 |
| irp3 | pchB | 11126738 | 11126735 | 223283.PSPTO_2599 | 223283.PSPTO_2596 | 0.841 | 0 | 0.49 | 0 | 0.267 | 0 | 0 | 0.458 | 0.963 |
| gpmA | eno-1 | 11129383 | 11125711 | 223283.PSPTO_5327 | 223283.PSPTO_1554 | 0.116 | 0 | 0 | 0 | 0.193 | 0 | 0.9 | 0.551 | 0.963 |
| hrpJ | hrcV | 11125561 | 11125560 | 223283.PSPTO_1403 | 223283.PSPTO_1402 | 0.891 | 0 | 0.491 | 0 | 0 | 0 | 0 | 0.375 | 0.962 |
| aceF | gltA | 11129065 | 11126346 | 223283.PSPTO_5006 | 223283.PSPTO_2194 | 0.078 | 0 | 0 | 0 | 0.336 | 0.39 | 0.9 | 0.156 | 0.962 |
| hscB | iscA | 11125584 | 11125583 | 223283.PSPTO_1426 | 223283.PSPTO_1425 | 0.878 | 0 | 0.538 | 0 | 0.267 | 0.093 | 0 | 0.104 | 0.96 |
| PSPTO_2601 | irp1 | 11126740 | 11126739 | 223283.PSPTO_2601 | 223283.PSPTO_2600 | 0.847 | 0 | 0.723 | 0 | 0 | 0 | 0 | 0.084 | 0.958 |
| tpiA | eno-1 | 11128574 | 11125711 | 223283.PSPTO_4494 | 223283.PSPTO_1554 | 0.116 | 0 | 0.47 | 0 | 0.708 | 0.385 | 0 | 0.572 | 0.957 |
| glk | pgi | 11125449 | 11125130 | 223283.PSPTO_1289 | 223283.PSPTO_0959 | 0.243 | 0 | 0 | 0 | 0.052 | 0 | 0.9 | 0.46 | 0.956 |
| aceE-2 | lpdA | 11129064 | 11126353 | 223283.PSPTO_5005 | 223283.PSPTO_2201 | 0.125 | 0 | 0 | 0 | 0.34 | 0.126 | 0.9 | 0.267 | 0.956 |
| PSPTO_2601 | irp3 | 11126740 | 11126738 | 223283.PSPTO_2601 | 223283.PSPTO_2599 | 0.859 | 0 | 0.701 | 0 | 0.051 | 0 | 0 | 0 | 0.956 |
| aceE-1 | lpdA | 11127959 | 11126353 | 223283.PSPTO_3860 | 223283.PSPTO_2201 | 0.125 | 0 | 0 | 0 | 0.34 | 0.126 | 0.9 | 0.267 | 0.956 |
| eno-2 | pgi | 11128692 | 11125130 | 223283.PSPTO_4616 | 223283.PSPTO_0959 | 0.113 | 0 | 0 | 0 | 0.705 | 0.125 | 0.8 | 0.188 | 0.956 |
| irp1 | pchA | 11126739 | 11126734 | 223283.PSPTO_2600 | 223283.PSPTO_2595 | 0.868 | 0 | 0.235 | 0 | 0.269 | 0 | 0 | 0.458 | 0.955 |
| iscA | iscU | 11125583 | 11125582 | 223283.PSPTO_1425 | 223283.PSPTO_1424 | 0.794 | 0.199 | 0.41 | 0 | 0.375 | 0 | 0 | 0.364 | 0.954 |
| PSPTO_5561 | PSPTO_3256 | 11129609 | 11127366 | 223283.PSPTO_5561 | 223283.PSPTO_3256 | 0.217 | 0 | 0.426 | 0 | 0.381 | 0.258 | 0.8 | 0.104 | 0.954 |
| fbp | pgi | 11129224 | 11125130 | 223283.PSPTO_5168 | 223283.PSPTO_0959 | 0 | 0 | 0.154 | 0 | 0.064 | 0 | 0.9 | 0.461 | 0.951 |
| pyk | rpoB | 11128419 | 11124799 | 223283.PSPTO_4337 | 223283.PSPTO_0619 | 0.127 | 0 | 0 | 0 | 0.09 | 0 | 0.9 | 0.459 | 0.951 |
| acnB | PSPTO_2289 | 11127851 | 11126439 | 223283.PSPTO_3752 | 223283.PSPTO_2289 | 0.116 | 0 | 0 | 0 | 0.061 | 0.094 | 0.9 | 0.459 | 0.951 |
| hrcC | hrpE | 11125547 | 11125544 | 223283.PSPTO_1389 | 223283.PSPTO_1386 | 0.617 | 0 | 0.781 | 0 | 0.131 | 0 | 0 | 0.415 | 0.951 |
| hrcQb | hrcU | 11125554 | 11125550 | 223283.PSPTO_1396 | 223283.PSPTO_1392 | 0.718 | 0 | 0.666 | 0 | 0.157 | 0 | 0 | 0.441 | 0.949 |
| hrpJ | hrcQa | 11125561 | 11125555 | 223283.PSPTO_1403 | 223283.PSPTO_1397 | 0.76 | 0 | 0.784 | 0 | 0 | 0 | 0 | 0.085 | 0.948 |
| irp5 | pchB | 11126736 | 11126735 | 223283.PSPTO_2597 | 223283.PSPTO_2596 | 0.856 | 0 | 0.192 | 0 | 0.267 | 0 | 0 | 0.458 | 0.947 |
| PSPTO_2602 | irp5 | 11126741 | 11126736 | 223283.PSPTO_2602 | 223283.PSPTO_2597 | 0.833 | 0 | 0.773 | 0.596 | 0.545 | 0 | 0 | 0.104 | 0.947 |
| rpoN | pilR | 11128533 | 11125003 | 223283.PSPTO_4453 | 223283.PSPTO_0823 | 0.056 | 0 | 0.715 | 0 | 0 | 0 | 0.8 | 0.143 | 0.947 |
| oadA | pyk | 11129558 | 11128419 | 223283.PSPTO_5510 | 223283.PSPTO_4337 | 0.086 | 0 | 0 | 0 | 0 | 0 | 0.9 | 0.458 | 0.946 |
| cfa6 | cfa5 | 11128759 | 11128758 | 223283.PSPTO_4686 | 223283.PSPTO_4685 | 0.847 | 0 | 0.649 | 0 | 0 | 0 | 0 | 0.076 | 0.946 |
| cmaB | cmaA | 11128779 | 11128778 | 223283.PSPTO_4710 | 223283.PSPTO_4709 | 0.845 | 0 | 0.492 | 0 | 0 | 0 | 0 | 0.375 | 0.946 |
| hrpV | hrpG | 11125549 | 11125546 | 223283.PSPTO_1391 | 223283.PSPTO_1388 | 0.845 | 0 | 0 | 0 | 0 | 0 | 0 | 0.667 | 0.946 |
| pgi | algC | 11125130 | 11124280 | 223283.PSPTO_0959 | 223283.PSPTO_0083 | 0.317 | 0 | 0 | 0 | 0.052 | 0 | 0.9 | 0.213 | 0.942 |
| pcD | acs | 11126045 | 11125981 | 223283.PSPTO_1891 | 223283.PSPTO_1825 | 0.067 | 0 | 0 | 0 | 0.264 | 0.102 | 0.9 | 0.211 | 0.942 |
| cmaT | cmaB | 11128781 | 11128779 | 223283.PSPTO_4712 | 223283.PSPTO_4710 | 0.833 | 0 | 0.489 | 0 | 0 | 0 | 0 | 0.375 | 0.942 |
| cfa7 | cfa5 | 11128760 | 11128758 | 223283.PSPTO_4687 | 223283.PSPTO_4685 | 0.847 | 0 | 0.624 | 0 | 0 | 0 | 0 | 0.076 | 0.942 |
| rpoS | rpoB | 11125722 | 11124799 | 223283.PSPTO_1565 | 223283.PSPTO_0619 | 0.056 | 0 | 0.342 | 0 | 0.052 | 0.895 | 0 | 0.197 | 0.941 |
| rpoS | rpoA | 11125722 | 11124831 | 223283.PSPTO_1565 | 223283.PSPTO_0651 | 0.043 | 0 | 0.358 | 0 | 0.059 | 0.898 | 0 | 0.148 | 0.94 |
| cfa9 | cfa7 | 11128763 | 11128760 | 223283.PSPTO_4690 | 223283.PSPTO_4687 | 0.551 | 0.125 | 0.735 | 0 | 0 | 0 | 0 | 0.493 | 0.94 |
| pvsA | PSPTO_2134 | 11126289 | 11126288 | 223283.PSPTO_2135 | 223283.PSPTO_2134 | 0.702 | 0 | 0.774 | 0 | 0.116 | 0 | 0 | 0.107 | 0.939 |
| tpiA | pyk | 11128574 | 11128419 | 223283.PSPTO_4494 | 223283.PSPTO_4337 | 0.125 | 0 | 0.243 | 0 | 0.84 | 0 | 0 | 0.5 | 0.939 |
| aceF | sucD | 11129065 | 11126355 | 223283.PSPTO_5006 | 223283.PSPTO_2203 | 0.116 | 0 | 0 | 0 | 0.602 | 0.093 | 0.8 | 0.188 | 0.938 |
| aceE-1 | pgi | 11127959 | 11125130 | 223283.PSPTO_3860 | 223283.PSPTO_0959 | 0 | 0 | 0 | 0 | 0.062 | 0 | 0.8 | 0.696 | 0.938 |
| aceE-2 | pgi | 11129064 | 11125130 | 223283.PSPTO_5005 | 223283.PSPTO_0959 | 0 | 0 | 0 | 0 | 0.062 | 0 | 0.8 | 0.696 | 0.938 |
| PSPTO_2602 | PSPTO_2601 | 11126741 | 11126740 | 223283.PSPTO_2602 | 223283.PSPTO_2601 | 0.845 | 0 | 0.618 | 0 | 0 | 0 | 0 | 0 | 0.938 |
| gltA | mqo | 11126346 | 11125301 | 223283.PSPTO_2194 | 223283.PSPTO_1136 | 0.056 | 0 | 0 | 0 | 0.312 | 0 | 0.9 | 0.154 | 0.937 |
| pyk | rpoC | 11128419 | 11124800 | 223283.PSPTO_4337 | 223283.PSPTO_0620 | 0.127 | 0 | 0 | 0 | 0.067 | 0 | 0.9 | 0.312 | 0.936 |
| cmaU | cmaC | 11128783 | 11128780 | 223283.PSPTO_4714 | 223283.PSPTO_4711 | 0.784 | 0 | 0.718 | 0 | 0 | 0 | 0 | 0 | 0.936 |
| gpmA | pgi | 11129383 | 11125130 | 223283.PSPTO_5327 | 223283.PSPTO_0959 | 0.08 | 0 | 0 | 0 | 0.115 | 0 | 0.8 | 0.655 | 0.936 |
| cmaC | cmaA | 11128780 | 11128778 | 223283.PSPTO_4711 | 223283.PSPTO_4709 | 0.837 | 0 | 0.429 | 0 | 0 | 0 | 0 | 0.375 | 0.936 |
| gpmA | eno-2 | 11129383 | 11128692 | 223283.PSPTO_5327 | 223283.PSPTO_4616 | 0.116 | 0 | 0 | 0 | 0.193 | 0 | 0.9 | 0.188 | 0.934 |
| cfa5 | cfa3 | 11128758 | 11128756 | 223283.PSPTO_4685 | 223283.PSPTO_4683 | 0.857 | 0 | 0.535 | 0 | 0 | 0 | 0 | 0.067 | 0.932 |
| PSPTO_4339 | mqo | 11128421 | 11125301 | 223283.PSPTO_4339 | 223283.PSPTO_1136 | 0.262 | 0 | 0 | 0 | 0 | 0 | 0.9 | 0.153 | 0.932 |
| PSPTO_4339 | gltA | 11128421 | 11126346 | 223283.PSPTO_4339 | 223283.PSPTO_2194 | 0.126 | 0 | 0 | 0 | 0.142 | 0 | 0.8 | 0.605 | 0.932 |
| cmaT | cmaC | 11128781 | 11128780 | 223283.PSPTO_4712 | 223283.PSPTO_4711 | 0.837 | 0 | 0.381 | 0 | 0 | 0 | 0 | 0.375 | 0.931 |
| pyk | rpoA | 11128419 | 11124831 | 223283.PSPTO_4337 | 223283.PSPTO_0651 | 0 | 0 | 0 | 0 | 0.084 | 0.123 | 0.9 | 0.245 | 0.931 |
| PSPTO_5465 | acs | 11129513 | 11125981 | 223283.PSPTO_5465 | 223283.PSPTO_1825 | 0 | 0 | 0 | 0 | 0.13 | 0.164 | 0.9 | 0.153 | 0.93 |
| PSPTO_5562 | PSPTO_5560 | 11129610 | 11129608 | 223283.PSPTO_5562 | 223283.PSPTO_5560 | 0.88 | 0 | 0.366 | 0 | 0.111 | 0 | 0 | 0.083 | 0.93 |
| hrcQb | hrpE | 11125554 | 11125544 | 223283.PSPTO_1396 | 223283.PSPTO_1386 | 0.068 | 0 | 0.784 | 0 | 0.481 | 0.355 | 0 | 0.104 | 0.928 |
| hrcQa | hrpE | 11125555 | 11125544 | 223283.PSPTO_1397 | 223283.PSPTO_1386 | 0.068 | 0 | 0.785 | 0 | 0.481 | 0.355 | 0 | 0.104 | 0.928 |
| sdhA | gltA | 11126349 | 11126346 | 223283.PSPTO_2197 | 223283.PSPTO_2194 | 0.533 | 0 | 0.316 | 0 | 0.388 | 0.416 | 0 | 0.461 | 0.927 |
| eno-2 | tpiA | 11128692 | 11128574 | 223283.PSPTO_4616 | 223283.PSPTO_4494 | 0.116 | 0 | 0.493 | 0 | 0.708 | 0.385 | 0 | 0.204 | 0.924 |
| PSPTO_5465 | aceF | 11129513 | 11129065 | 223283.PSPTO_5465 | 223283.PSPTO_5006 | 0 | 0 | 0.176 | 0 | 0.089 | 0.1 | 0.9 | 0 | 0.923 |
| cfa4 | cfa3 | 11128757 | 11128756 | 223283.PSPTO_4684 | 223283.PSPTO_4683 | 0.857 | 0.482 | 0 | 0 | 0 | 0 | 0 | 0 | 0.923 |
| PSPTO_5465 | sdhB | 11129513 | 11126350 | 223283.PSPTO_5465 | 223283.PSPTO_2198 | 0 | 0 | 0 | 0 | 0.236 | 0 | 0.9 | 0.072 | 0.922 |
| hrcQa | hrcU | 11125555 | 11125550 | 223283.PSPTO_1397 | 223283.PSPTO_1392 | 0.718 | 0 | 0.612 | 0 | 0.157 | 0 | 0 | 0.25 | 0.921 |
| cfa9 | cfa6 | 11128763 | 11128759 | 223283.PSPTO_4690 | 223283.PSPTO_4686 | 0.551 | 0.074 | 0.717 | 0 | 0 | 0 | 0 | 0.402 | 0.92 |
| PSPTO_4339 | PSPTO_2196 | 11128421 | 11126348 | 223283.PSPTO_4339 | 223283.PSPTO_2196 | 0.407 | 0 | 0 | 0 | 0.495 | 0 | 0.65 | 0.327 | 0.92 |
| sdhB | gltA | 11126350 | 11126346 | 223283.PSPTO_2198 | 223283.PSPTO_2194 | 0.529 | 0 | 0.327 | 0 | 0.548 | 0.42 | 0 | 0.188 | 0.92 |
| oadA | aceE-2 | 11129558 | 11129064 | 223283.PSPTO_5510 | 223283.PSPTO_5005 | 0 | 0 | 0 | 0 | 0 | 0 | 0.9 | 0.226 | 0.919 |
| oadA | aceE-1 | 11129558 | 11127959 | 223283.PSPTO_5510 | 223283.PSPTO_3860 | 0 | 0 | 0 | 0 | 0 | 0 | 0.9 | 0.226 | 0.919 |
| hrcV | hrcC | 11125560 | 11125547 | 223283.PSPTO_1402 | 223283.PSPTO_1389 | 0.352 | 0 | 0.576 | 0 | 0.265 | 0 | 0 | 0.648 | 0.919 |
| rpoA | rpoH | 11124831 | 11124614 | 223283.PSPTO_0651 | 223283.PSPTO_0430 | 0.043 | 0 | 0.72 | 0 | 0.059 | 0.501 | 0 | 0.461 | 0.919 |
| PSPTO_5465 | sdhA | 11129513 | 11126349 | 223283.PSPTO_5465 | 223283.PSPTO_2197 | 0 | 0 | 0 | 0 | 0.142 | 0.093 | 0.9 | 0.075 | 0.918 |
| hrcV | hrcQb | 11125560 | 11125554 | 223283.PSPTO_1402 | 223283.PSPTO_1396 | 0.76 | 0 | 0.571 | 0 | 0.162 | 0 | 0 | 0.157 | 0.918 |
| gabD-3 | sdhA | 11126816 | 11126349 | 223283.PSPTO_2680 | 223283.PSPTO_2197 | 0.044 | 0 | 0 | 0 | 0.124 | 0.093 | 0.9 | 0.08 | 0.917 |
| sdhA | gabD-1 | 11126349 | 11124444 | 223283.PSPTO_2197 | 223283.PSPTO_0257 | 0.044 | 0 | 0 | 0 | 0.124 | 0.093 | 0.9 | 0.08 | 0.917 |
| aceF | acs | 11129065 | 11125981 | 223283.PSPTO_5006 | 223283.PSPTO_1825 | 0.084 | 0 | 0 | 0 | 0.06 | 0 | 0.9 | 0.154 | 0.917 |
| purB | argH | 11127470 | 11124321 | 223283.PSPTO_3360 | 223283.PSPTO_0125 | 0.086 | 0 | 0 | 0 | 0.064 | 0 | 0.9 | 0.143 | 0.916 |
| hrcQb | hrcR | 11125554 | 11125553 | 223283.PSPTO_1396 | 223283.PSPTO_1395 | 0.718 | 0 | 0.455 | 0 | 0.141 | 0 | 0 | 0.444 | 0.916 |
| PSPTO_2604 | PSPTO_2603 | 11126743 | 11126742 | 223283.PSPTO_2604 | 223283.PSPTO_2603 | 0.891 | 0 | 0.784 | 0.802 | 0 | 0 | 0 | 0.457 | 0.916 |
| hrpJ | hrcU | 11125561 | 11125550 | 223283.PSPTO_1403 | 223283.PSPTO_1392 | 0.697 | 0 | 0.597 | 0 | 0 | 0 | 0 | 0.364 | 0.915 |
| PSPTO_3256 | fecC | 11127366 | 11124942 | 223283.PSPTO_3256 | 223283.PSPTO_0762 | 0.059 | 0 | 0.413 | 0 | 0.218 | 0.101 | 0.8 | 0.104 | 0.914 |
| PSPTO_2604 | irp3 | 11126743 | 11126738 | 223283.PSPTO_2604 | 223283.PSPTO_2599 | 0.749 | 0 | 0.592 | 0 | 0.121 | 0 | 0 | 0.149 | 0.913 |
| oadA | gltA | 11129558 | 11126346 | 223283.PSPTO_5510 | 223283.PSPTO_2194 | 0.042 | 0 | 0 | 0 | 0 | 0 | 0.9 | 0.154 | 0.911 |
| irp3 | pchA | 11126738 | 11126734 | 223283.PSPTO_2599 | 223283.PSPTO_2595 | 0.836 | 0 | 0.323 | 0 | 0.266 | 0 | 0 | 0 | 0.911 |
| hrpV | hrpT | 11125549 | 11125548 | 223283.PSPTO_1391 | 223283.PSPTO_1390 | 0.845 | 0 | 0 | 0 | 0 | 0 | 0 | 0.449 | 0.911 |
| glk | algC | 11125449 | 11124280 | 223283.PSPTO_1289 | 223283.PSPTO_0083 | 0 | 0 | 0 | 0 | 0 | 0 | 0.9 | 0.15 | 0.911 |
| sdhC | gabD-1 | 11126347 | 11124444 | 223283.PSPTO_2195 | 223283.PSPTO_0257 | 0 | 0 | 0 | 0 | 0.092 | 0.093 | 0.9 | 0 | 0.91 |
| aceE-2 | gltA | 11129064 | 11126346 | 223283.PSPTO_5005 | 223283.PSPTO_2194 | 0 | 0 | 0 | 0 | 0 | 0 | 0.9 | 0.143 | 0.91 |
| PSPTO_5490 | glk | 11129538 | 11125449 | 223283.PSPTO_5490 | 223283.PSPTO_1289 | 0.123 | 0 | 0 | 0 | 0.052 | 0 | 0.9 | 0.051 | 0.91 |
| PSPTO_5465 | sucB | 11129513 | 11126352 | 223283.PSPTO_5465 | 223283.PSPTO_2200 | 0 | 0 | 0 | 0 | 0.089 | 0.1 | 0.9 | 0 | 0.91 |
| aceE-1 | gltA | 11127959 | 11126346 | 223283.PSPTO_3860 | 223283.PSPTO_2194 | 0 | 0 | 0 | 0 | 0 | 0 | 0.9 | 0.143 | 0.91 |
| gabD-3 | sdhC | 11126816 | 11126347 | 223283.PSPTO_2680 | 223283.PSPTO_2195 | 0 | 0 | 0 | 0 | 0.092 | 0.093 | 0.9 | 0 | 0.91 |
| cfa5 | cfa2 | 11128758 | 11128755 | 223283.PSPTO_4685 | 223283.PSPTO_4682 | 0.858 | 0 | 0.37 | 0 | 0 | 0 | 0 | 0.072 | 0.91 |
| adhB | pcD | 11128368 | 11126045 | 223283.PSPTO_4285 | 223283.PSPTO_1891 | 0.054 | 0.002 | 0 | 0 | 0.091 | 0 | 0.9 | 0.074 | 0.909 |
| cfa3 | cfa1 | 11128756 | 11128754 | 223283.PSPTO_4683 | 223283.PSPTO_4681 | 0.828 | 0 | 0 | 0 | 0.117 | 0.425 | 0 | 0.077 | 0.909 |
| pyk | aceE-1 | 11128419 | 11127959 | 223283.PSPTO_4337 | 223283.PSPTO_3860 | 0 | 0 | 0 | 0 | 0 | 0 | 0.9 | 0.124 | 0.908 |
| cfa2 | cfa1 | 11128755 | 11128754 | 223283.PSPTO_4682 | 223283.PSPTO_4681 | 0.833 | 0 | 0 | 0 | 0.06 | 0.435 | 0 | 0.082 | 0.908 |
| aceE-2 | pyk | 11129064 | 11128419 | 223283.PSPTO_5005 | 223283.PSPTO_4337 | 0 | 0 | 0 | 0 | 0 | 0 | 0.9 | 0.124 | 0.908 |
| sdhC | gltA | 11126347 | 11126346 | 223283.PSPTO_2195 | 223283.PSPTO_2194 | 0.533 | 0 | 0.31 | 0 | 0.527 | 0 | 0 | 0.461 | 0.907 |
| PSPTO_5465 | sdhC | 11129513 | 11126347 | 223283.PSPTO_5465 | 223283.PSPTO_2195 | 0 | 0 | 0 | 0 | 0.101 | 0 | 0.9 | 0 | 0.906 |
| PSPTO_5561 | PSPTO_5560 | 11129609 | 11129608 | 223283.PSPTO_5561 | 223283.PSPTO_5560 | 0.847 | 0 | 0.37 | 0 | 0.1 | 0 | 0 | 0 | 0.906 |
| cfa8 | cfa7 | 11128762 | 11128760 | 223283.PSPTO_4689 | 223283.PSPTO_4687 | 0.684 | 0 | 0.465 | 0 | 0 | 0 | 0 | 0.488 | 0.906 |
| sucB | sdhC | 11126352 | 11126347 | 223283.PSPTO_2200 | 223283.PSPTO_2195 | 0.569 | 0 | 0 | 0 | 0.563 | 0 | 0 | 0.536 | 0.905 |
| irp4 | pchA | 11126737 | 11126734 | 223283.PSPTO_2598 | 223283.PSPTO_2595 | 0.81 | 0.004 | 0 | 0 | 0.497 | 0 | 0 | 0.083 | 0.905 |
| PSPTO_5465 | gabD-3 | 11129513 | 11126816 | 223283.PSPTO_5465 | 223283.PSPTO_2680 | 0.051 | 0 | 0 | 0 | 0.061 | 0 | 0.9 | 0.066 | 0.905 |
| PSPTO_5465 | gabD-1 | 11129513 | 11124444 | 223283.PSPTO_5465 | 223283.PSPTO_0257 | 0.051 | 0 | 0 | 0 | 0.061 | 0 | 0.9 | 0.066 | 0.905 |
| PSPTO_5465 | pcD | 11129513 | 11126045 | 223283.PSPTO_5465 | 223283.PSPTO_1891 | 0.051 | 0 | 0 | 0 | 0.061 | 0 | 0.9 | 0.067 | 0.905 |
| eno-1 | gap-1 | 11125711 | 11125447 | 223283.PSPTO_1554 | 223283.PSPTO_1287 | 0.068 | 0 | 0.511 | 0 | 0.578 | 0.408 | 0 | 0.299 | 0.905 |
| glk | PSPTO_1047 | 11125449 | 11125215 | 223283.PSPTO_1289 | 223283.PSPTO_1047 | 0.055 | 0 | 0 | 0 | 0 | 0 | 0.9 | 0.076 | 0.905 |
| PSPTO_2601 | irp4 | 11126740 | 11126737 | 223283.PSPTO_2601 | 223283.PSPTO_2598 | 0.851 | 0 | 0.37 | 0 | 0.057 | 0 | 0 | 0 | 0.904 |
| sucB | sdhB | 11126352 | 11126350 | 223283.PSPTO_2200 | 223283.PSPTO_2198 | 0.575 | 0 | 0.153 | 0 | 0.525 | 0.127 | 0 | 0.459 | 0.904 |
| PSPTO_2602 | irp1 | 11126741 | 11126739 | 223283.PSPTO_2602 | 223283.PSPTO_2600 | 0.849 | 0 | 0.782 | 0.584 | 0.061 | 0 | 0 | 0.104 | 0.903 |
| PSPTO_2601 | irp5 | 11126740 | 11126736 | 223283.PSPTO_2601 | 223283.PSPTO_2597 | 0.845 | 0 | 0.376 | 0 | 0.061 | 0 | 0 | 0.054 | 0.903 |
| PSPTO_5490 | algC | 11129538 | 11124280 | 223283.PSPTO_5490 | 223283.PSPTO_0083 | 0 | 0 | 0 | 0 | 0 | 0 | 0.9 | 0.076 | 0.903 |
| oadA | mqo | 11129558 | 11125301 | 223283.PSPTO_5510 | 223283.PSPTO_1136 | 0.076 | 0 | 0 | 0 | 0 | 0 | 0.9 | 0 | 0.903 |
| aceE-2 | aceE-1 | 11129064 | 11127959 | 223283.PSPTO_5005 | 223283.PSPTO_3860 | 0 | 0 | 0.785 | 0.968 | 0 | 0 | 0.9 | 0 | 0.902 |
| PSPTO_5562 | fecC | 11129610 | 11124942 | 223283.PSPTO_5562 | 223283.PSPTO_0762 | 0.059 | 0.01 | 0.336 | 0 | 0.218 | 0.101 | 0.8 | 0.104 | 0.902 |
| hrcV | hrcQa | 11125560 | 11125555 | 223283.PSPTO_1402 | 223283.PSPTO_1397 | 0.76 | 0 | 0.488 | 0 | 0.162 | 0 | 0 | 0.158 | 0.902 |
| PSPTO_5490 | pgi | 11129538 | 11125130 | 223283.PSPTO_5490 | 223283.PSPTO_0959 | 0 | 0 | 0 | 0 | 0.042 | 0 | 0.9 | 0.059 | 0.901 |
| PSPTO_5465 | PSPTO_2196 | 11129513 | 11126348 | 223283.PSPTO_5465 | 223283.PSPTO_2196 | 0.043 | 0 | 0 | 0 | 0 | 0 | 0.9 | 0 | 0.9 |
| hrpV | hrcC | 11125549 | 11125547 | 223283.PSPTO_1391 | 223283.PSPTO_1389 | 0.845 | 0 | 0 | 0 | 0 | 0 | 0 | 0.375 | 0.899 |
| cmaB | cmaE | 11128779 | 11128777 | 223283.PSPTO_4710 | 223283.PSPTO_4708 | 0.845 | 0 | 0 | 0 | 0 | 0 | 0 | 0.375 | 0.899 |
| hrpT | hrpG | 11125548 | 11125546 | 223283.PSPTO_1390 | 223283.PSPTO_1388 | 0.845 | 0 | 0 | 0 | 0 | 0 | 0 | 0.375 | 0.899 |
| cmaA | cmaE | 11128778 | 11128777 | 223283.PSPTO_4709 | 223283.PSPTO_4708 | 0.845 | 0 | 0 | 0 | 0 | 0 | 0 | 0.375 | 0.899 |
| hrcC | hrpG | 11125547 | 11125546 | 223283.PSPTO_1389 | 223283.PSPTO_1388 | 0.845 | 0 | 0 | 0 | 0 | 0 | 0 | 0.374 | 0.899 |
| hrpT | hrcC | 11125548 | 11125547 | 223283.PSPTO_1390 | 223283.PSPTO_1389 | 0.845 | 0 | 0 | 0 | 0 | 0 | 0 | 0.375 | 0.899 |
| irp1 | pchB | 11126739 | 11126735 | 223283.PSPTO_2600 | 223283.PSPTO_2596 | 0.805 | 0 | 0.414 | 0 | 0.083 | 0.093 | 0 | 0.091 | 0.898 |
| cfa7 | cfa6 | 11128760 | 11128759 | 223283.PSPTO_4687 | 223283.PSPTO_4686 | 0.852 | 0 | 0.784 | 0.763 | 0 | 0 | 0 | 0.54 | 0.894 |
| PSPTO_2603 | irp3 | 11126742 | 11126738 | 223283.PSPTO_2603 | 223283.PSPTO_2599 | 0.73 | 0 | 0.558 | 0 | 0.121 | 0 | 0 | 0.105 | 0.893 |
| cmaC | cmaE | 11128780 | 11128777 | 223283.PSPTO_4711 | 223283.PSPTO_4708 | 0.837 | 0 | 0 | 0 | 0 | 0 | 0 | 0.375 | 0.893 |
| sucB | PSPTO_2196 | 11126352 | 11126348 | 223283.PSPTO_2200 | 223283.PSPTO_2196 | 0.569 | 0 | 0 | 0 | 0.575 | 0 | 0 | 0.461 | 0.892 |
| hrpL | hrpJ | 11125562 | 11125561 | 223283.PSPTO_1404 | 223283.PSPTO_1403 | 0.475 | 0 | 0.512 | 0 | 0 | 0 | 0 | 0.61 | 0.891 |
| cmaB | cmaD | 11128779 | 11128776 | 223283.PSPTO_4710 | 223283.PSPTO_4707 | 0.831 | 0 | 0 | 0 | 0 | 0 | 0 | 0.375 | 0.89 |
| cmaE | cmaD | 11128777 | 11128776 | 223283.PSPTO_4708 | 223283.PSPTO_4707 | 0.831 | 0 | 0 | 0 | 0 | 0 | 0 | 0.375 | 0.89 |
| cmaA | cmaD | 11128778 | 11128776 | 223283.PSPTO_4709 | 223283.PSPTO_4707 | 0.831 | 0 | 0 | 0 | 0 | 0 | 0 | 0.375 | 0.89 |
| PSPTO_4339 | acnB | 11128421 | 11127851 | 223283.PSPTO_4339 | 223283.PSPTO_3752 | 0.378 | 0 | 0.171 | 0 | 0.448 | 0 | 0 | 0.653 | 0.888 |
| iscU | cyaY | 11125582 | 11124414 | 223283.PSPTO_1424 | 223283.PSPTO_0227 | 0 | 0 | 0.614 | 0 | 0.062 | 0.499 | 0 | 0.459 | 0.888 |
| rpoC | rpoH | 11124800 | 11124614 | 223283.PSPTO_0620 | 223283.PSPTO_0430 | 0.056 | 0 | 0.675 | 0 | 0.052 | 0.501 | 0 | 0.348 | 0.888 |
| cmaT | cmaE | 11128781 | 11128777 | 223283.PSPTO_4712 | 223283.PSPTO_4708 | 0.828 | 0 | 0 | 0 | 0 | 0 | 0 | 0.375 | 0.888 |
| sucB | sdhA | 11126352 | 11126349 | 223283.PSPTO_2200 | 223283.PSPTO_2197 | 0.569 | 0 | 0 | 0 | 0.527 | 0.093 | 0 | 0.464 | 0.887 |
| PSPTO_2602 | pchA | 11126741 | 11126734 | 223283.PSPTO_2602 | 223283.PSPTO_2595 | 0.805 | 0.009 | 0.235 | 0 | 0.269 | 0 | 0 | 0.082 | 0.887 |
| PSPTO_2147 | PSPTO_2146 | 11126301 | 11126300 | 223283.PSPTO_2147 | 223283.PSPTO_2146 | 0.43 | 0.349 | 0.707 | 0 | 0.061 | 0 | 0 | 0.064 | 0.887 |
| cfa6 | cfa2 | 11128759 | 11128755 | 223283.PSPTO_4686 | 223283.PSPTO_4682 | 0.847 | 0 | 0.234 | 0 | 0 | 0 | 0 | 0.083 | 0.884 |
| sucD | acs | 11126355 | 11125981 | 223283.PSPTO_2203 | 223283.PSPTO_1825 | 0.053 | 0 | 0.277 | 0 | 0.155 | 0 | 0.8 | 0.156 | 0.884 |
| PSPTO_2149 | PSPTO_2146 | 11126303 | 11126300 | 223283.PSPTO_2149 | 223283.PSPTO_2146 | 0.416 | 0.369 | 0.698 | 0 | 0.061 | 0 | 0 | 0.064 | 0.884 |
| cmaC | cmaD | 11128780 | 11128776 | 223283.PSPTO_4711 | 223283.PSPTO_4707 | 0.823 | 0 | 0 | 0 | 0 | 0 | 0 | 0.375 | 0.884 |
| hscB | cyaY | 11125584 | 11124414 | 223283.PSPTO_1426 | 223283.PSPTO_0227 | 0 | 0 | 0.768 | 0 | 0.14 | 0 | 0 | 0.461 | 0.883 |
| cfa7 | cfa2 | 11128760 | 11128755 | 223283.PSPTO_4687 | 223283.PSPTO_4682 | 0.847 | 0 | 0.229 | 0 | 0 | 0 | 0 | 0.083 | 0.883 |
| PSPTO_2148 | PSPTO_2146 | 11126302 | 11126300 | 223283.PSPTO_2148 | 223283.PSPTO_2146 | 0.43 | 0.23 | 0.741 | 0 | 0.061 | 0 | 0 | 0.064 | 0.882 |
| PSPTO_0402 | PSPTO_0401 | 11124586 | 11124585 | 223283.PSPTO_0402 | 223283.PSPTO_0401 | 0.372 | 0.083 | 0.773 | 0 | 0.061 | 0 | 0 | 0.192 | 0.882 |
| hopN1 | shcN | 11125528 | 11125527 | 223283.PSPTO_1370 | 223283.PSPTO_1369 | 0.816 | 0 | 0 | 0 | 0 | 0 | 0 | 0.374 | 0.88 |
| PSPTO_2604 | irp1 | 11126743 | 11126739 | 223283.PSPTO_2604 | 223283.PSPTO_2600 | 0.684 | 0 | 0.536 | 0 | 0.064 | 0.123 | 0 | 0.152 | 0.879 |
| cmaT | cmaD | 11128781 | 11128776 | 223283.PSPTO_4712 | 223283.PSPTO_4707 | 0.815 | 0 | 0 | 0 | 0 | 0 | 0 | 0.375 | 0.879 |
| PSPTO_2150 | PSPTO_2146 | 11126304 | 11126300 | 223283.PSPTO_2150 | 223283.PSPTO_2146 | 0.416 | 0.218 | 0.744 | 0 | 0.061 | 0 | 0 | 0.064 | 0.878 |
| PSPTO_2601 | pchB | 11126740 | 11126735 | 223283.PSPTO_2601 | 223283.PSPTO_2596 | 0.802 | 0 | 0.404 | 0 | 0 | 0 | 0 | 0 | 0.877 |
| cfa7 | cfa3 | 11128760 | 11128756 | 223283.PSPTO_4687 | 223283.PSPTO_4683 | 0.847 | 0 | 0.458 | 0.605 | 0 | 0 | 0 | 0.078 | 0.876 |
| PSPTO_2602 | pchB | 11126741 | 11126735 | 223283.PSPTO_2602 | 223283.PSPTO_2596 | 0.802 | 0 | 0.355 | 0 | 0.083 | 0 | 0 | 0.062 | 0.876 |
| cfa6 | cfa3 | 11128759 | 11128756 | 223283.PSPTO_4686 | 223283.PSPTO_4683 | 0.847 | 0 | 0.481 | 0.617 | 0 | 0 | 0 | 0.078 | 0.876 |
| rpoB | rpoH | 11124799 | 11124614 | 223283.PSPTO_0619 | 223283.PSPTO_0430 | 0.056 | 0 | 0.693 | 0 | 0.052 | 0.501 | 0 | 0.241 | 0.876 |
| hrcQb | hrcC | 11125554 | 11125547 | 223283.PSPTO_1396 | 223283.PSPTO_1389 | 0.058 | 0 | 0.783 | 0 | 0.09 | 0 | 0 | 0.407 | 0.875 |
| cmaU | cmaB | 11128783 | 11128779 | 223283.PSPTO_4714 | 223283.PSPTO_4710 | 0.776 | 0 | 0.462 | 0 | 0 | 0 | 0 | 0 | 0.874 |
| PSPTO_2604 | irp5 | 11126743 | 11126736 | 223283.PSPTO_2604 | 223283.PSPTO_2597 | 0.751 | 0 | 0.388 | 0 | 0.061 | 0 | 0 | 0.213 | 0.872 |
| eno-2 | gap-1 | 11128692 | 11125447 | 223283.PSPTO_4616 | 223283.PSPTO_1287 | 0.068 | 0 | 0.452 | 0 | 0.578 | 0.408 | 0 | 0.136 | 0.869 |
| oadA | argH | 11129558 | 11124321 | 223283.PSPTO_5510 | 223283.PSPTO_0125 | 0.371 | 0 | 0 | 0 | 0 | 0 | 0.8 | 0 | 0.868 |
| PSPTO_2603 | irp1 | 11126742 | 11126739 | 223283.PSPTO_2603 | 223283.PSPTO_2600 | 0.684 | 0 | 0.486 | 0 | 0.064 | 0.123 | 0 | 0.152 | 0.866 |
| PSPTO_2148 | PSPTO_2147 | 11126302 | 11126301 | 223283.PSPTO_2148 | 223283.PSPTO_2147 | 0.845 | 0 | 0.784 | 0.835 | 0 | 0 | 0 | 0 | 0.865 |
| cfa9 | cfa8 | 11128763 | 11128762 | 223283.PSPTO_4690 | 223283.PSPTO_4689 | 0.668 | 0 | 0.159 | 0 | 0 | 0 | 0 | 0.55 | 0.863 |
| PSPTO_4339 | sucD | 11128421 | 11126355 | 223283.PSPTO_4339 | 223283.PSPTO_2203 | 0.407 | 0 | 0 | 0 | 0.389 | 0 | 0 | 0.653 | 0.863 |
| irp4 | pchB | 11126737 | 11126735 | 223283.PSPTO_2598 | 223283.PSPTO_2596 | 0.81 | 0 | 0.204 | 0 | 0.119 | 0 | 0 | 0.08 | 0.862 |
| PSPTO_2601 | pchA | 11126740 | 11126734 | 223283.PSPTO_2601 | 223283.PSPTO_2595 | 0.802 | 0 | 0.298 | 0 | 0.064 | 0 | 0 | 0.059 | 0.861 |
| cfa5 | cfa4 | 11128758 | 11128757 | 223283.PSPTO_4685 | 223283.PSPTO_4684 | 0.858 | 0 | 0 | 0 | 0 | 0 | 0 | 0 | 0.859 |
| cfa4 | cfa2 | 11128757 | 11128755 | 223283.PSPTO_4684 | 223283.PSPTO_4682 | 0.858 | 0 | 0 | 0 | 0 | 0 | 0 | 0 | 0.859 |
| hrpJ | hrpE | 11125561 | 11125544 | 223283.PSPTO_1403 | 223283.PSPTO_1386 | 0 | 0 | 0.784 | 0 | 0 | 0 | 0 | 0.374 | 0.859 |
| rpoN | hrpS | 11128533 | 11125538 | 223283.PSPTO_4453 | 223283.PSPTO_1380 | 0.061 | 0 | 0.761 | 0 | 0 | 0 | 0 | 0.414 | 0.857 |
| PSPTO_2150 | PSPTO_2149 | 11126304 | 11126303 | 223283.PSPTO_2150 | 223283.PSPTO_2149 | 0.845 | 0 | 0.784 | 0.9 | 0 | 0 | 0 | 0 | 0.857 |
| cmaC | iaaL | 11128780 | 11124556 | 223283.PSPTO_4711 | 223283.PSPTO_0371 | 0 | 0 | 0.783 | 0 | 0 | 0 | 0 | 0.372 | 0.857 |
| cmaA | cfa6 | 11128778 | 11128759 | 223283.PSPTO_4709 | 223283.PSPTO_4686 | 0.056 | 0.08 | 0.707 | 0 | 0.06 | 0 | 0 | 0.496 | 0.857 |
| cfa5 | cfa1 | 11128758 | 11128754 | 223283.PSPTO_4685 | 223283.PSPTO_4681 | 0.841 | 0 | 0 | 0 | 0 | 0.101 | 0 | 0.075 | 0.856 |
| rpoN | hrpR | 11128533 | 11125537 | 223283.PSPTO_4453 | 223283.PSPTO_1379 | 0.061 | 0 | 0.76 | 0 | 0 | 0 | 0 | 0.414 | 0.856 |
| hrpJ | hrcC | 11125561 | 11125547 | 223283.PSPTO_1403 | 223283.PSPTO_1389 | 0 | 0 | 0.781 | 0 | 0 | 0 | 0 | 0.372 | 0.856 |
| oadA | acs | 11129558 | 11125981 | 223283.PSPTO_5510 | 223283.PSPTO_1825 | 0.126 | 0 | 0 | 0 | 0 | 0 | 0.8 | 0.239 | 0.855 |
| oadA | aceF | 11129558 | 11129065 | 223283.PSPTO_5510 | 223283.PSPTO_5006 | 0 | 0 | 0 | 0 | 0.088 | 0 | 0.8 | 0.272 | 0.855 |
| PSPTO_2604 | PSPTO_2602 | 11126743 | 11126741 | 223283.PSPTO_2604 | 223283.PSPTO_2602 | 0.656 | 0 | 0.57 | 0 | 0.064 | 0 | 0 | 0.059 | 0.852 |
| PSPTO_4339 | argH | 11128421 | 11124321 | 223283.PSPTO_4339 | 223283.PSPTO_0125 | 0.196 | 0 | 0 | 0 | 0.062 | 0 | 0.8 | 0.135 | 0.852 |
| hrcQa | hrcR | 11125555 | 11125553 | 223283.PSPTO_1397 | 223283.PSPTO_1395 | 0.718 | 0 | 0.355 | 0 | 0.141 | 0 | 0 | 0.147 | 0.849 |
| hrcU | hrpE | 11125550 | 11125544 | 223283.PSPTO_1392 | 223283.PSPTO_1386 | 0.116 | 0 | 0.594 | 0 | 0.333 | 0 | 0 | 0.447 | 0.849 |
| cfa6 | cfa4 | 11128759 | 11128757 | 223283.PSPTO_4686 | 223283.PSPTO_4684 | 0.847 | 0 | 0 | 0 | 0 | 0 | 0 | 0 | 0.848 |
| PSPTO_2150 | PSPTO_2147 | 11126304 | 11126301 | 223283.PSPTO_2150 | 223283.PSPTO_2147 | 0.811 | 0 | 0.784 | 0.84 | 0 | 0 | 0 | 0.542 | 0.848 |
| cfa7 | cfa4 | 11128760 | 11128757 | 223283.PSPTO_4687 | 223283.PSPTO_4684 | 0.847 | 0 | 0 | 0 | 0 | 0 | 0 | 0 | 0.848 |
| PSPTO_2605 | PSPTO_2601 | 11126744 | 11126740 | 223283.PSPTO_2605 | 223283.PSPTO_2601 | 0.409 | 0 | 0.745 | 0 | 0 | 0 | 0 | 0 | 0.843 |
| PSPTO_2605 | irp3 | 11126744 | 11126738 | 223283.PSPTO_2605 | 223283.PSPTO_2599 | 0.426 | 0 | 0.702 | 0 | 0 | 0 | 0 | 0.15 | 0.842 |
| PSPTO_5562 | PSPTO_3256 | 11129610 | 11127366 | 223283.PSPTO_5562 | 223283.PSPTO_3256 | 0 | 0 | 0.588 | 0.657 | 0 | 0 | 0.8 | 0 | 0.839 |
| cmaA | cfa7 | 11128778 | 11128760 | 223283.PSPTO_4709 | 223283.PSPTO_4687 | 0.056 | 0.369 | 0.736 | 0 | 0.06 | 0 | 0 | 0.075 | 0.838 |
| PSPTO_2603 | irp5 | 11126742 | 11126736 | 223283.PSPTO_2603 | 223283.PSPTO_2597 | 0.75 | 0 | 0.322 | 0 | 0.061 | 0 | 0 | 0.104 | 0.838 |
| PSPTO_2149 | PSPTO_2148 | 11126303 | 11126302 | 223283.PSPTO_2149 | 223283.PSPTO_2148 | 0.811 | 0 | 0.784 | 0.817 | 0 | 0 | 0 | 0 | 0.838 |
| PSPTO_2605 | irp1 | 11126744 | 11126739 | 223283.PSPTO_2605 | 223283.PSPTO_2600 | 0.416 | 0 | 0.679 | 0 | 0.141 | 0 | 0 | 0.104 | 0.836 |
| cfa7 | cfa1 | 11128760 | 11128754 | 223283.PSPTO_4687 | 223283.PSPTO_4681 | 0.828 | 0 | 0 | 0 | 0 | 0 | 0 | 0.083 | 0.836 |
| cfa6 | cfa1 | 11128759 | 11128754 | 223283.PSPTO_4686 | 223283.PSPTO_4681 | 0.828 | 0 | 0 | 0 | 0 | 0 | 0 | 0.083 | 0.836 |
| hrpL | hrcQb | 11125562 | 11125554 | 223283.PSPTO_1404 | 223283.PSPTO_1396 | 0.45 | 0 | 0.514 | 0 | 0 | 0.116 | 0 | 0.372 | 0.832 |
| cfa4 | cfa1 | 11128757 | 11128754 | 223283.PSPTO_4684 | 223283.PSPTO_4681 | 0.831 | 0 | 0 | 0 | 0 | 0 | 0 | 0 | 0.831 |
| sucB | gltA | 11126352 | 11126346 | 223283.PSPTO_2200 | 223283.PSPTO_2194 | 0.434 | 0 | 0.168 | 0 | 0.336 | 0.39 | 0 | 0.251 | 0.831 |
| gltA | acs | 11126346 | 11125981 | 223283.PSPTO_2194 | 223283.PSPTO_1825 | 0.115 | 0 | 0 | 0 | 0.642 | 0.129 | 0 | 0.458 | 0.83 |
| PSPTO_2147 | PSPTO_2134 | 11126301 | 11126288 | 223283.PSPTO_2147 | 223283.PSPTO_2134 | 0.054 | 0.131 | 0.779 | 0 | 0.155 | 0 | 0 | 0.067 | 0.83 |
| gltA | argH | 11126346 | 11124321 | 223283.PSPTO_2194 | 223283.PSPTO_0125 | 0.044 | 0 | 0 | 0 | 0.06 | 0 | 0.8 | 0.161 | 0.829 |
| gltA | gabD-1 | 11126346 | 11124444 | 223283.PSPTO_2194 | 223283.PSPTO_0257 | 0.051 | 0 | 0 | 0 | 0.141 | 0 | 0.8 | 0.076 | 0.829 |
| gabD-3 | gltA | 11126816 | 11126346 | 223283.PSPTO_2680 | 223283.PSPTO_2194 | 0.051 | 0 | 0 | 0 | 0.141 | 0 | 0.8 | 0.076 | 0.829 |
| PSPTO_2603 | PSPTO_2602 | 11126742 | 11126741 | 223283.PSPTO_2603 | 223283.PSPTO_2602 | 0.658 | 0 | 0.498 | 0 | 0.064 | 0 | 0 | 0.058 | 0.828 |
| PSPTO_5465 | sucD | 11129513 | 11126355 | 223283.PSPTO_5465 | 223283.PSPTO_2203 | 0 | 0 | 0 | 0 | 0.064 | 0.103 | 0.8 | 0.088 | 0.826 |
| gabD-3 | mqo | 11126816 | 11125301 | 223283.PSPTO_2680 | 223283.PSPTO_1136 | 0 | 0 | 0 | 0 | 0.141 | 0 | 0.8 | 0.067 | 0.825 |
| mqo | gabD-1 | 11125301 | 11124444 | 223283.PSPTO_1136 | 223283.PSPTO_0257 | 0 | 0 | 0 | 0 | 0.141 | 0 | 0.8 | 0.067 | 0.825 |
| PSPTO_2149 | PSPTO_2147 | 11126303 | 11126301 | 223283.PSPTO_2149 | 223283.PSPTO_2147 | 0.811 | 0 | 0.784 | 0.911 | 0 | 0 | 0 | 0.082 | 0.824 |
| cmaU | cmaT | 11128783 | 11128781 | 223283.PSPTO_4714 | 223283.PSPTO_4712 | 0.792 | 0 | 0.192 | 0 | 0 | 0 | 0 | 0 | 0.824 |
| irp5 | PSPTO_2134 | 11126736 | 11126288 | 223283.PSPTO_2597 | 223283.PSPTO_2134 | 0.084 | 0.007 | 0.586 | 0 | 0.52 | 0 | 0 | 0.14 | 0.822 |
| PSPTO_2605 | PSPTO_2604 | 11126744 | 11126743 | 223283.PSPTO_2605 | 223283.PSPTO_2604 | 0.549 | 0 | 0.533 | 0 | 0.062 | 0 | 0 | 0.203 | 0.821 |
| PSPTO_3062 | PSPTO_0402 | 11127184 | 11124586 | 223283.PSPTO_3062 | 223283.PSPTO_0402 | 0.125 | 0.084 | 0.751 | 0 | 0.061 | 0 | 0 | 0.192 | 0.821 |
| hrpJ | hrcR | 11125561 | 11125553 | 223283.PSPTO_1403 | 223283.PSPTO_1395 | 0.731 | 0 | 0.346 | 0 | 0 | 0 | 0 | 0.059 | 0.82 |
| aceE-2 | acs | 11129064 | 11125981 | 223283.PSPTO_5005 | 223283.PSPTO_1825 | 0.056 | 0 | 0 | 0 | 0 | 0 | 0.8 | 0.119 | 0.819 |
| aceE-1 | acs | 11127959 | 11125981 | 223283.PSPTO_3860 | 223283.PSPTO_1825 | 0.056 | 0 | 0 | 0 | 0 | 0 | 0.8 | 0.119 | 0.819 |
| irp1 | PSPTO_2134 | 11126739 | 11126288 | 223283.PSPTO_2600 | 223283.PSPTO_2134 | 0.312 | 0.025 | 0.579 | 0 | 0.155 | 0 | 0 | 0.35 | 0.819 |
| purB | gltA | 11127470 | 11126346 | 223283.PSPTO_3360 | 223283.PSPTO_2194 | 0 | 0 | 0 | 0 | 0.091 | 0 | 0.8 | 0.082 | 0.818 |
| gabD-3 | gabD-1 | 11126816 | 11124444 | 223283.PSPTO_2680 | 223283.PSPTO_0257 | 0 | 0 | 0.781 | 0.894 | 0 | 0 | 0.8 | 0 | 0.816 |
| cmaB | iaaL | 11128779 | 11124556 | 223283.PSPTO_4710 | 223283.PSPTO_0371 | 0 | 0 | 0.72 | 0 | 0 | 0 | 0 | 0.371 | 0.816 |
| PSPTO_2150 | PSPTO_2148 | 11126304 | 11126302 | 223283.PSPTO_2150 | 223283.PSPTO_2148 | 0.811 | 0 | 0.785 | 0.962 | 0 | 0 | 0 | 0 | 0.816 |
| cfa9 | irp5 | 11128763 | 11126736 | 223283.PSPTO_4690 | 223283.PSPTO_2597 | 0.084 | 0.003 | 0.573 | 0 | 0.52 | 0 | 0 | 0.14 | 0.816 |
| PSPTO_2604 | PSPTO_2601 | 11126743 | 11126740 | 223283.PSPTO_2604 | 223283.PSPTO_2601 | 0.655 | 0 | 0.484 | 0 | 0.051 | 0 | 0 | 0 | 0.816 |
| PSPTO_4339 | gabD-3 | 11128421 | 11126816 | 223283.PSPTO_4339 | 223283.PSPTO_2680 | 0 | 0 | 0 | 0 | 0.084 | 0 | 0.8 | 0.075 | 0.815 |
| PSPTO_4339 | gabD-1 | 11128421 | 11124444 | 223283.PSPTO_4339 | 223283.PSPTO_0257 | 0 | 0 | 0 | 0 | 0.084 | 0 | 0.8 | 0.075 | 0.815 |
| oadA | gabD-1 | 11129558 | 11124444 | 223283.PSPTO_5510 | 223283.PSPTO_0257 | 0 | 0.006 | 0 | 0 | 0 | 0.095 | 0.8 | 0.059 | 0.814 |
| oadA | gabD-3 | 11129558 | 11126816 | 223283.PSPTO_5510 | 223283.PSPTO_2680 | 0 | 0.003 | 0 | 0 | 0 | 0.095 | 0.8 | 0.059 | 0.814 |
| PSPTO_4339 | PSPTO_4338 | 11128421 | 11128420 | 223283.PSPTO_4339 | 223283.PSPTO_4338 | 0.661 | 0 | 0 | 0 | 0.068 | 0 | 0 | 0.457 | 0.813 |
| PSPTO_2149 | PSPTO_2134 | 11126303 | 11126288 | 223283.PSPTO_2149 | 223283.PSPTO_2134 | 0.054 | 0.011 | 0.779 | 0 | 0.155 | 0 | 0 | 0.067 | 0.813 |
| mqo | argH | 11125301 | 11124321 | 223283.PSPTO_1136 | 223283.PSPTO_0125 | 0.083 | 0 | 0 | 0 | 0.048 | 0 | 0.8 | 0.059 | 0.813 |
| oadA | purB | 11129558 | 11127470 | 223283.PSPTO_5510 | 223283.PSPTO_3360 | 0.084 | 0 | 0 | 0 | 0.064 | 0 | 0.8 | 0 | 0.813 |
| irp4 | PSPTO_2149 | 11126737 | 11126303 | 223283.PSPTO_2598 | 223283.PSPTO_2149 | 0.054 | 0.008 | 0.778 | 0 | 0.155 | 0 | 0 | 0.068 | 0.812 |
| sdhB | argH | 11126350 | 11124321 | 223283.PSPTO_2198 | 223283.PSPTO_0125 | 0 | 0 | 0 | 0 | 0.061 | 0 | 0.8 | 0.082 | 0.812 |
| cmaU | cmaA | 11128783 | 11128778 | 223283.PSPTO_4714 | 223283.PSPTO_4709 | 0.776 | 0 | 0.19 | 0 | 0 | 0 | 0 | 0 | 0.811 |
| PSPTO_2148 | PSPTO_2134 | 11126302 | 11126288 | 223283.PSPTO_2148 | 223283.PSPTO_2134 | 0.054 | 0 | 0.776 | 0 | 0.155 | 0 | 0 | 0.067 | 0.81 |
| irp4 | PSPTO_2147 | 11126737 | 11126301 | 223283.PSPTO_2598 | 223283.PSPTO_2147 | 0.054 | 0.021 | 0.774 | 0 | 0.155 | 0 | 0 | 0.068 | 0.809 |
| PSPTO_2150 | PSPTO_2134 | 11126304 | 11126288 | 223283.PSPTO_2150 | 223283.PSPTO_2134 | 0.054 | 0 | 0.775 | 0 | 0.155 | 0 | 0 | 0.067 | 0.809 |
| PSPTO_2603 | PSPTO_2601 | 11126742 | 11126740 | 223283.PSPTO_2603 | 223283.PSPTO_2601 | 0.655 | 0 | 0.461 | 0 | 0.051 | 0 | 0 | 0 | 0.808 |
| irp4 | PSPTO_2148 | 11126737 | 11126302 | 223283.PSPTO_2598 | 223283.PSPTO_2148 | 0.054 | 0 | 0.773 | 0 | 0.155 | 0 | 0 | 0.068 | 0.808 |
| cfa9 | PSPTO_2147 | 11128763 | 11126301 | 223283.PSPTO_4690 | 223283.PSPTO_2147 | 0.054 | 0.029 | 0.773 | 0 | 0.155 | 0 | 0 | 0.067 | 0.808 |
| hrcQa | hrcC | 11125555 | 11125547 | 223283.PSPTO_1397 | 223283.PSPTO_1389 | 0.058 | 0 | 0.782 | 0 | 0.09 | 0 | 0 | 0.097 | 0.808 |
| eno-2 | eno-1 | 11128692 | 11125711 | 223283.PSPTO_4616 | 223283.PSPTO_1554 | 0 | 0 | 0.784 | 0.951 | 0 | 0 | 0.8 | 0 | 0.807 |
| sdhA | argH | 11126349 | 11124321 | 223283.PSPTO_2197 | 223283.PSPTO_0125 | 0 | 0 | 0 | 0 | 0.052 | 0 | 0.8 | 0.064 | 0.807 |
| irp4 | pvsA | 11126737 | 11126289 | 223283.PSPTO_2598 | 223283.PSPTO_2135 | 0.112 | 0 | 0.755 | 0 | 0.116 | 0 | 0 | 0.107 | 0.805 |
| irp4 | PSPTO_2150 | 11126737 | 11126304 | 223283.PSPTO_2598 | 223283.PSPTO_2150 | 0.054 | 0 | 0.769 | 0 | 0.155 | 0 | 0 | 0.068 | 0.804 |
| cfa9 | PSPTO_2149 | 11128763 | 11126303 | 223283.PSPTO_4690 | 223283.PSPTO_2149 | 0.054 | 0.006 | 0.767 | 0 | 0.155 | 0 | 0 | 0.067 | 0.802 |
| purB | mqo | 11127470 | 11125301 | 223283.PSPTO_3360 | 223283.PSPTO_1136 | 0 | 0 | 0 | 0 | 0.048 | 0 | 0.8 | 0 | 0.801 |
| cfa7 | PSPTO_2147 | 11128760 | 11126301 | 223283.PSPTO_4687 | 223283.PSPTO_2147 | 0.056 | 0.419 | 0.648 | 0 | 0.06 | 0 | 0 | 0.075 | 0.801 |
| pyk | gap-1 | 11128419 | 11125447 | 223283.PSPTO_4337 | 223283.PSPTO_1287 | 0.125 | 0 | 0.174 | 0 | 0.644 | 0.12 | 0 | 0.253 | 0.8 |
| PSPTO_3190 | pcD | 11127305 | 11126045 | 223283.PSPTO_3190 | 223283.PSPTO_1891 | 0 | 0 | 0 | 0 | 0 | 0 | 0.8 | 0 | 0.8 |
| PSPTO_2196 | argH | 11126348 | 11124321 | 223283.PSPTO_2196 | 223283.PSPTO_0125 | 0 | 0 | 0 | 0 | 0 | 0 | 0.8 | 0 | 0.8 |
| sdhC | argH | 11126347 | 11124321 | 223283.PSPTO_2195 | 223283.PSPTO_0125 | 0 | 0 | 0 | 0 | 0 | 0 | 0.8 | 0 | 0.8 |
| cmaA | cfa9 | 11128778 | 11128763 | 223283.PSPTO_4709 | 223283.PSPTO_4690 | 0.054 | 0.051 | 0.754 | 0 | 0.155 | 0 | 0 | 0.067 | 0.794 |
| cmaT | PSPTO_2147 | 11128781 | 11126301 | 223283.PSPTO_4712 | 223283.PSPTO_2147 | 0.052 | 0.013 | 0.766 | 0 | 0.114 | 0 | 0 | 0.067 | 0.792 |
| PSPTO_2604 | pchA | 11126743 | 11126734 | 223283.PSPTO_2604 | 223283.PSPTO_2595 | 0.738 | 0 | 0.2 | 0 | 0.089 | 0 | 0 | 0 | 0.792 |
| PSPTO_2604 | irp4 | 11126743 | 11126737 | 223283.PSPTO_2604 | 223283.PSPTO_2598 | 0.66 | 0 | 0.319 | 0 | 0.052 | 0 | 0 | 0.152 | 0.789 |
| cfa9 | PSPTO_2148 | 11128763 | 11126302 | 223283.PSPTO_4690 | 223283.PSPTO_2148 | 0.054 | 0 | 0.748 | 0 | 0.155 | 0 | 0 | 0.067 | 0.786 |
| pvdE | pvsA | 11126307 | 11126289 | 223283.PSPTO_2153 | 223283.PSPTO_2135 | 0.087 | 0 | 0.757 | 0 | 0 | 0 | 0 | 0.107 | 0.784 |
| hrcV | hrpE | 11125560 | 11125544 | 223283.PSPTO_1402 | 223283.PSPTO_1386 | 0.116 | 0 | 0.476 | 0 | 0.266 | 0 | 0 | 0.442 | 0.784 |
| cfa9 | pvsA | 11128763 | 11126289 | 223283.PSPTO_4690 | 223283.PSPTO_2135 | 0.112 | 0 | 0.729 | 0 | 0.116 | 0 | 0 | 0.107 | 0.784 |
| gpmA | tpiA | 11129383 | 11128574 | 223283.PSPTO_5327 | 223283.PSPTO_4494 | 0.186 | 0 | 0 | 0 | 0.121 | 0 | 0 | 0.722 | 0.784 |
| cmaA | PSPTO_2134 | 11128778 | 11126288 | 223283.PSPTO_4709 | 223283.PSPTO_2134 | 0.054 | 0.156 | 0.71 | 0 | 0.155 | 0 | 0 | 0.067 | 0.784 |
| PSPTO_4569 | PSPTO_4338 | 11128647 | 11128420 | 223283.PSPTO_4569 | 223283.PSPTO_4338 | 0.253 | 0 | 0 | 0 | 0.085 | 0.479 | 0 | 0.464 | 0.783 |
| cfa8 | cfa6 | 11128762 | 11128759 | 223283.PSPTO_4689 | 223283.PSPTO_4686 | 0.684 | 0 | 0.473 | 0.585 | 0 | 0 | 0 | 0.397 | 0.783 |
| PSPTO_5424 | rpoN | 11129476 | 11128533 | 223283.PSPTO_5424 | 223283.PSPTO_4453 | 0 | 0 | 0.744 | 0 | 0 | 0 | 0 | 0.188 | 0.783 |
| cfa9 | PSPTO_2150 | 11128763 | 11126304 | 223283.PSPTO_4690 | 223283.PSPTO_2150 | 0.054 | 0 | 0.742 | 0 | 0.155 | 0 | 0 | 0.067 | 0.781 |
| sucD | gltA | 11126355 | 11126346 | 223283.PSPTO_2203 | 223283.PSPTO_2194 | 0.393 | 0 | 0 | 0 | 0.592 | 0 | 0 | 0.188 | 0.781 |
| hrpZ1 | hrpA1 | 11125540 | 11125539 | 223283.PSPTO_1382 | 223283.PSPTO_1381 | 0.663 | 0 | 0 | 0 | 0 | 0 | 0 | 0.376 | 0.78 |
| cmaT | PSPTO_2149 | 11128781 | 11126303 | 223283.PSPTO_4712 | 223283.PSPTO_2149 | 0.052 | 0 | 0.753 | 0 | 0.114 | 0 | 0 | 0.067 | 0.78 |
| cfa9 | irp1 | 11128763 | 11126739 | 223283.PSPTO_4690 | 223283.PSPTO_2600 | 0.312 | 0.012 | 0.486 | 0 | 0.155 | 0 | 0 | 0.35 | 0.779 |
| PSPTO_2605 | PSPTO_2603 | 11126744 | 11126742 | 223283.PSPTO_2605 | 223283.PSPTO_2603 | 0.549 | 0 | 0.484 | 0 | 0.062 | 0 | 0 | 0.109 | 0.779 |
| cmaT | irp5 | 11128781 | 11126736 | 223283.PSPTO_4712 | 223283.PSPTO_2597 | 0.079 | 0 | 0.542 | 0 | 0.463 | 0 | 0 | 0.134 | 0.777 |
| cmaU | cmaE | 11128783 | 11128777 | 223283.PSPTO_4714 | 223283.PSPTO_4708 | 0.776 | 0 | 0 | 0 | 0 | 0 | 0 | 0 | 0.776 |
| PSPTO_2602 | PSPTO_2134 | 11126741 | 11126288 | 223283.PSPTO_2602 | 223283.PSPTO_2134 | 0.054 | 0.079 | 0.724 | 0 | 0.155 | 0 | 0 | 0.067 | 0.776 |
| cfa8 | cfa5 | 11128762 | 11128758 | 223283.PSPTO_4689 | 223283.PSPTO_4685 | 0.679 | 0 | 0.319 | 0 | 0 | 0 | 0 | 0.058 | 0.776 |
| cmaT | cfa7 | 11128781 | 11128760 | 223283.PSPTO_4712 | 223283.PSPTO_4687 | 0.056 | 0.066 | 0.756 | 0 | 0 | 0 | 0 | 0.083 | 0.776 |
| pvdE | PSPTO_2146 | 11126307 | 11126300 | 223283.PSPTO_2153 | 223283.PSPTO_2146 | 0.215 | 0 | 0.715 | 0 | 0 | 0 | 0 | 0.075 | 0.774 |
| PSPTO_2603 | pchA | 11126742 | 11126734 | 223283.PSPTO_2603 | 223283.PSPTO_2595 | 0.718 | 0 | 0.189 | 0 | 0.089 | 0 | 0 | 0 | 0.773 |
| rpoN | PSPTO_0964 | 11128533 | 11125135 | 223283.PSPTO_4453 | 223283.PSPTO_0964 | 0.056 | 0 | 0.744 | 0 | 0 | 0 | 0 | 0.135 | 0.772 |
| pvdE | PSPTO_2150 | 11126307 | 11126304 | 223283.PSPTO_2153 | 223283.PSPTO_2150 | 0.056 | 0 | 0.755 | 0 | 0 | 0 | 0 | 0.076 | 0.767 |
| aceF | sdhA | 11129065 | 11126349 | 223283.PSPTO_5006 | 223283.PSPTO_2197 | 0.116 | 0 | 0 | 0 | 0.527 | 0.093 | 0 | 0.458 | 0.766 |
| PSPTO_2605 | PSPTO_2602 | 11126744 | 11126741 | 223283.PSPTO_2605 | 223283.PSPTO_2602 | 0.416 | 0 | 0.558 | 0 | 0.141 | 0 | 0 | 0.069 | 0.766 |
| pvdE | PSPTO_2148 | 11126307 | 11126302 | 223283.PSPTO_2153 | 223283.PSPTO_2148 | 0.056 | 0 | 0.753 | 0 | 0 | 0 | 0 | 0.076 | 0.765 |
| lpdA | sdhB | 11126353 | 11126350 | 223283.PSPTO_2201 | 223283.PSPTO_2198 | 0.492 | 0 | 0.162 | 0 | 0.257 | 0.21 | 0 | 0.196 | 0.763 |
| cmaU | cmaD | 11128783 | 11128776 | 223283.PSPTO_4714 | 223283.PSPTO_4707 | 0.763 | 0 | 0 | 0 | 0 | 0 | 0 | 0 | 0.763 |
| hrpJ | hrpW1 | 11125561 | 11125531 | 223283.PSPTO_1403 | 223283.PSPTO_1373 | 0 | 0 | 0.627 | 0 | 0 | 0 | 0 | 0.388 | 0.762 |
| cmaT | cfa6 | 11128781 | 11128759 | 223283.PSPTO_4712 | 223283.PSPTO_4686 | 0.056 | 0.046 | 0.746 | 0 | 0 | 0 | 0 | 0.083 | 0.762 |
| cfa7 | cfl | 11128760 | 11128753 | 223283.PSPTO_4687 | 223283.PSPTO_4680 | 0.588 | 0.126 | 0.368 | 0 | 0 | 0 | 0 | 0.076 | 0.762 |
| PSPTO_2603 | irp4 | 11126742 | 11126737 | 223283.PSPTO_2603 | 223283.PSPTO_2598 | 0.66 | 0 | 0.265 | 0 | 0.052 | 0 | 0 | 0.106 | 0.76 |
| cfa9 | PSPTO_2602 | 11128763 | 11126741 | 223283.PSPTO_4690 | 223283.PSPTO_2602 | 0.054 | 0.138 | 0.683 | 0 | 0.155 | 0 | 0 | 0.067 | 0.759 |
| lpdA | sdhC | 11126353 | 11126347 | 223283.PSPTO_2201 | 223283.PSPTO_2195 | 0.49 | 0 | 0 | 0 | 0.192 | 0 | 0 | 0.457 | 0.757 |
| rpoN | PSPTO_4292 | 11128533 | 11128375 | 223283.PSPTO_4453 | 223283.PSPTO_4292 | 0.056 | 0 | 0.726 | 0 | 0 | 0 | 0 | 0.135 | 0.756 |
| cmaT | pvsA | 11128781 | 11126289 | 223283.PSPTO_4712 | 223283.PSPTO_2135 | 0.112 | 0 | 0.692 | 0 | 0.116 | 0 | 0 | 0.107 | 0.755 |
| cfa6 | cfl | 11128759 | 11128753 | 223283.PSPTO_4686 | 223283.PSPTO_4680 | 0.588 | 0.05 | 0.401 | 0 | 0 | 0 | 0 | 0.076 | 0.755 |
| cfa5 | iaaL | 11128758 | 11124556 | 223283.PSPTO_4685 | 223283.PSPTO_0371 | 0.056 | 0 | 0.746 | 0 | 0 | 0 | 0 | 0.06 | 0.754 |
| hrpL | hrcQa | 11125562 | 11125555 | 223283.PSPTO_1404 | 223283.PSPTO_1397 | 0.45 | 0 | 0.533 | 0 | 0 | 0.116 | 0 | 0 | 0.753 |
| hopB1 | hrpK1 | 11125564 | 11125563 | 223283.PSPTO_1406 | 223283.PSPTO_1405 | 0.618 | 0 | 0 | 0 | 0 | 0 | 0 | 0.374 | 0.751 |
| cmaT | PSPTO_2148 | 11128781 | 11126302 | 223283.PSPTO_4712 | 223283.PSPTO_2148 | 0.052 | 0 | 0.717 | 0 | 0.114 | 0 | 0 | 0.067 | 0.748 |
| cmaT | irp1 | 11128781 | 11126739 | 223283.PSPTO_4712 | 223283.PSPTO_2600 | 0.312 | 0.005 | 0.441 | 0 | 0.114 | 0 | 0 | 0.35 | 0.748 |
| pvdE | PSPTO_2147 | 11126307 | 11126301 | 223283.PSPTO_2153 | 223283.PSPTO_2147 | 0.056 | 0 | 0.733 | 0 | 0 | 0 | 0 | 0.076 | 0.746 |
| PSPTO_2604 | pchB | 11126743 | 11126735 | 223283.PSPTO_2604 | 223283.PSPTO_2596 | 0.655 | 0 | 0.232 | 0 | 0.088 | 0 | 0 | 0.07 | 0.745 |
| cfa8 | cfa3 | 11128762 | 11128756 | 223283.PSPTO_4689 | 223283.PSPTO_4683 | 0.679 | 0 | 0.228 | 0 | 0.052 | 0 | 0 | 0 | 0.745 |
| oadA | PSPTO_4339 | 11129558 | 11128421 | 223283.PSPTO_5510 | 223283.PSPTO_4339 | 0.514 | 0 | 0 | 0 | 0.1 | 0 | 0 | 0.462 | 0.744 |
| hrcU | hrcC | 11125550 | 11125547 | 223283.PSPTO_1392 | 223283.PSPTO_1389 | 0.084 | 0 | 0.651 | 0 | 0.157 | 0 | 0 | 0.159 | 0.743 |
| PSPTO_2603 | pchB | 11126742 | 11126735 | 223283.PSPTO_2603 | 223283.PSPTO_2596 | 0.657 | 0 | 0.216 | 0 | 0.088 | 0 | 0 | 0.07 | 0.741 |
| gltA | rpoD | 11126346 | 11124718 | 223283.PSPTO_2194 | 223283.PSPTO_0537 | 0 | 0 | 0 | 0 | 0 | 0 | 0 | 0.74 | 0.74 |
| rpoN | PSPTO_0111 | 11128533 | 11124308 | 223283.PSPTO_4453 | 223283.PSPTO_0111 | 0.061 | 0 | 0.717 | 0 | 0 | 0 | 0 | 0.102 | 0.74 |
| rpoN | rpoS | 11128533 | 11125722 | 223283.PSPTO_4453 | 223283.PSPTO_1565 | 0 | 0 | 0 | 0 | 0 | 0 | 0 | 0.739 | 0.739 |
| cmaT | PSPTO_2150 | 11128781 | 11126304 | 223283.PSPTO_4712 | 223283.PSPTO_2150 | 0.052 | 0 | 0.706 | 0 | 0.114 | 0 | 0 | 0.067 | 0.738 |
| pvdE | PSPTO_2149 | 11126307 | 11126303 | 223283.PSPTO_2153 | 223283.PSPTO_2149 | 0.056 | 0 | 0.723 | 0 | 0 | 0 | 0 | 0.076 | 0.737 |
| rpoN | rpoD | 11128533 | 11124718 | 223283.PSPTO_4453 | 223283.PSPTO_0537 | 0 | 0 | 0 | 0 | 0 | 0 | 0 | 0.736 | 0.736 |
| hrpV | hrpS | 11125549 | 11125538 | 223283.PSPTO_1391 | 223283.PSPTO_1380 | 0.285 | 0 | 0 | 0 | 0 | 0 | 0 | 0.644 | 0.734 |
| hrcR | hrcC | 11125553 | 11125547 | 223283.PSPTO_1395 | 223283.PSPTO_1389 | 0.129 | 0 | 0.475 | 0 | 0.264 | 0 | 0 | 0.299 | 0.732 |
| irp5 | PSPTO_2147 | 11126736 | 11126301 | 223283.PSPTO_2597 | 223283.PSPTO_2147 | 0.057 | 0.174 | 0.706 | 0.586 | 0.545 | 0 | 0 | 0.104 | 0.732 |
| hrpL | hrpR | 11125562 | 11125537 | 223283.PSPTO_1404 | 223283.PSPTO_1379 | 0 | 0 | 0.172 | 0 | 0 | 0 | 0 | 0.688 | 0.731 |
| gltA | pgi | 11126346 | 11125130 | 223283.PSPTO_2194 | 223283.PSPTO_0959 | 0.053 | 0 | 0.165 | 0 | 0.087 | 0.093 | 0 | 0.651 | 0.73 |
| cfa3 | cfl | 11128756 | 11128753 | 223283.PSPTO_4683 | 223283.PSPTO_4680 | 0.586 | 0.012 | 0.354 | 0 | 0 | 0 | 0 | 0.067 | 0.729 |
| hrpT | hrpE | 11125548 | 11125544 | 223283.PSPTO_1390 | 223283.PSPTO_1386 | 0.581 | 0 | 0 | 0 | 0 | 0 | 0 | 0.375 | 0.727 |
| hrpG | hrpE | 11125546 | 11125544 | 223283.PSPTO_1388 | 223283.PSPTO_1386 | 0.581 | 0 | 0 | 0 | 0 | 0 | 0 | 0.375 | 0.727 |
| rpoN | rpoA | 11128533 | 11124831 | 223283.PSPTO_4453 | 223283.PSPTO_0651 | 0 | 0 | 0 | 0 | 0 | 0.517 | 0 | 0.457 | 0.726 |
| hrpV | hrpE | 11125549 | 11125544 | 223283.PSPTO_1391 | 223283.PSPTO_1386 | 0.581 | 0 | 0 | 0 | 0 | 0 | 0 | 0.374 | 0.726 |
| pvdE | PSPTO_2134 | 11126307 | 11126288 | 223283.PSPTO_2153 | 223283.PSPTO_2134 | 0.084 | 0 | 0.71 | 0 | 0 | 0 | 0 | 0 | 0.723 |
| cfa7 | PSPTO_2134 | 11128760 | 11126288 | 223283.PSPTO_4687 | 223283.PSPTO_2134 | 0.068 | 0.267 | 0.609 | 0 | 0 | 0 | 0 | 0.083 | 0.722 |
| PSPTO_2146 | pvsA | 11126300 | 11126289 | 223283.PSPTO_2146 | 223283.PSPTO_2135 | 0.129 | 0.1 | 0.654 | 0 | 0.052 | 0 | 0 | 0.082 | 0.721 |
| PSPTO_2196 | gltA | 11126348 | 11126346 | 223283.PSPTO_2196 | 223283.PSPTO_2194 | 0.53 | 0 | 0.206 | 0 | 0.263 | 0 | 0 | 0.099 | 0.719 |
| cfa5 | cfl | 11128758 | 11128753 | 223283.PSPTO_4685 | 223283.PSPTO_4680 | 0.585 | 0 | 0.772 | 0.584 | 0 | 0 | 0 | 0 | 0.717 |
| cfa8 | cfa2 | 11128762 | 11128755 | 223283.PSPTO_4689 | 223283.PSPTO_4682 | 0.679 | 0 | 0.15 | 0 | 0 | 0 | 0 | 0 | 0.716 |
| rpoN | phhR | 11128533 | 11125979 | 223283.PSPTO_4453 | 223283.PSPTO_1823 | 0 | 0 | 0.704 | 0 | 0 | 0 | 0 | 0.072 | 0.713 |
| lpdA | sdhA | 11126353 | 11126349 | 223283.PSPTO_2201 | 223283.PSPTO_2197 | 0.49 | 0 | 0 | 0 | 0.268 | 0.134 | 0 | 0.213 | 0.712 |
| cmaC | cfa5 | 11128780 | 11128758 | 223283.PSPTO_4711 | 223283.PSPTO_4685 | 0 | 0 | 0.71 | 0 | 0 | 0 | 0 | 0 | 0.71 |
| cfl | iaaL | 11128753 | 11124556 | 223283.PSPTO_4680 | 223283.PSPTO_0371 | 0.056 | 0 | 0.698 | 0 | 0 | 0 | 0 | 0.06 | 0.708 |
| hrpS | hrpR | 11125538 | 11125537 | 223283.PSPTO_1380 | 223283.PSPTO_1379 | 0.691 | 0 | 0.785 | 0.96 | 0 | 0 | 0 | 0.542 | 0.707 |
| PSPTO_2605 | irp5 | 11126744 | 11126736 | 223283.PSPTO_2605 | 223283.PSPTO_2597 | 0.409 | 0 | 0.297 | 0 | 0.268 | 0 | 0 | 0.147 | 0.706 |
| hrpL | hrcV | 11125562 | 11125560 | 223283.PSPTO_1404 | 223283.PSPTO_1402 | 0.475 | 0 | 0.181 | 0 | 0 | 0 | 0 | 0.37 | 0.705 |
| pyk | gltA | 11128419 | 11126346 | 223283.PSPTO_4337 | 223283.PSPTO_2194 | 0.086 | 0 | 0 | 0 | 0.101 | 0.408 | 0 | 0.458 | 0.701 |
| PSPTO_2146 | PSPTO_2134 | 11126300 | 11126288 | 223283.PSPTO_2146 | 223283.PSPTO_2134 | 0.084 | 0 | 0.683 | 0 | 0.05 | 0 | 0 | 0 | 0.7 |
| rpoN | rpoC | 11128533 | 11124800 | 223283.PSPTO_4453 | 223283.PSPTO_0620 | 0 | 0 | 0 | 0 | 0 | 0.529 | 0 | 0.382 | 0.696 |
| hrpL | hrpE | 11125562 | 11125544 | 223283.PSPTO_1404 | 223283.PSPTO_1386 | 0 | 0 | 0.535 | 0 | 0 | 0 | 0 | 0.37 | 0.694 |
| cmaA | irp4 | 11128778 | 11126737 | 223283.PSPTO_4709 | 223283.PSPTO_2598 | 0.054 | 0.015 | 0.637 | 0 | 0.155 | 0 | 0 | 0.068 | 0.693 |
| rpoN | rpoB | 11128533 | 11124799 | 223283.PSPTO_4453 | 223283.PSPTO_0619 | 0 | 0 | 0 | 0 | 0 | 0.53 | 0 | 0.372 | 0.692 |
| PSPTO_4339 | acs | 11128421 | 11125981 | 223283.PSPTO_4339 | 223283.PSPTO_1825 | 0.406 | 0 | 0 | 0 | 0.114 | 0 | 0 | 0.461 | 0.691 |
| irp5 | PSPTO_2149 | 11126736 | 11126303 | 223283.PSPTO_2597 | 223283.PSPTO_2149 | 0.057 | 0 | 0.73 | 0.598 | 0.545 | 0 | 0 | 0.104 | 0.69 |
| cfa8 | cfa1 | 11128762 | 11128754 | 223283.PSPTO_4689 | 223283.PSPTO_4681 | 0.664 | 0 | 0 | 0 | 0.084 | 0 | 0 | 0.07 | 0.689 |
| sdhB | gabD-1 | 11126350 | 11124444 | 223283.PSPTO_2198 | 223283.PSPTO_0257 | 0.048 | 0 | 0 | 0 | 0.106 | 0 | 0.65 | 0.076 | 0.687 |
| irp5 | PSPTO_2150 | 11126736 | 11126304 | 223283.PSPTO_2597 | 223283.PSPTO_2150 | 0.057 | 0 | 0.709 | 0.594 | 0.545 | 0 | 0 | 0.104 | 0.687 |
| gabD-3 | sdhB | 11126816 | 11126350 | 223283.PSPTO_2680 | 223283.PSPTO_2198 | 0.048 | 0 | 0 | 0 | 0.106 | 0 | 0.65 | 0.076 | 0.687 |
| irp5 | PSPTO_2148 | 11126736 | 11126302 | 223283.PSPTO_2597 | 223283.PSPTO_2148 | 0.057 | 0 | 0.711 | 0.598 | 0.545 | 0 | 0 | 0.104 | 0.686 |
| PSPTO_2605 | pchB | 11126744 | 11126735 | 223283.PSPTO_2605 | 223283.PSPTO_2596 | 0.39 | 0 | 0.501 | 0 | 0 | 0 | 0 | 0 | 0.683 |
| hrcR | hrpE | 11125553 | 11125544 | 223283.PSPTO_1395 | 223283.PSPTO_1386 | 0.116 | 0 | 0.338 | 0 | 0.141 | 0 | 0 | 0.444 | 0.683 |
| PSPTO_4339 | PSPTO_2289 | 11128421 | 11126439 | 223283.PSPTO_4339 | 223283.PSPTO_2289 | 0.124 | 0 | 0 | 0 | 0.159 | 0 | 0 | 0.605 | 0.683 |
| cmaT | PSPTO_2602 | 11128781 | 11126741 | 223283.PSPTO_4712 | 223283.PSPTO_2602 | 0.052 | 0 | 0.643 | 0 | 0.114 | 0 | 0 | 0.067 | 0.682 |
| rpoS | rpoC | 11125722 | 11124800 | 223283.PSPTO_1565 | 223283.PSPTO_0620 | 0.056 | 0 | 0.288 | 0 | 0.052 | 0.502 | 0 | 0.149 | 0.68 |
| hrpA1 | hrpS | 11125539 | 11125538 | 223283.PSPTO_1381 | 223283.PSPTO_1380 | 0.583 | 0 | 0 | 0 | 0 | 0 | 0 | 0.263 | 0.679 |
| cfa8 | cfa4 | 11128762 | 11128757 | 223283.PSPTO_4689 | 223283.PSPTO_4684 | 0.679 | 0 | 0 | 0 | 0 | 0 | 0 | 0 | 0.679 |
| hrpE | hrpW1 | 11125544 | 11125531 | 223283.PSPTO_1386 | 223283.PSPTO_1373 | 0 | 0 | 0.678 | 0 | 0 | 0 | 0 | 0 | 0.678 |
| cmaB | cfa6 | 11128779 | 11128759 | 223283.PSPTO_4710 | 223283.PSPTO_4686 | 0.053 | 0 | 0.663 | 0 | 0.051 | 0 | 0 | 0.064 | 0.678 |
| PSPTO_2196 | gabD-1 | 11126348 | 11124444 | 223283.PSPTO_2196 | 223283.PSPTO_0257 | 0 | 0 | 0 | 0 | 0.102 | 0 | 0.65 | 0 | 0.672 |
| gabD-3 | PSPTO_2196 | 11126816 | 11126348 | 223283.PSPTO_2680 | 223283.PSPTO_2196 | 0 | 0 | 0 | 0 | 0.102 | 0 | 0.65 | 0 | 0.672 |
| sdhC | cyoE | 11126347 | 11125487 | 223283.PSPTO_2195 | 223283.PSPTO_1329 | 0 | 0 | 0.161 | 0 | 0.483 | 0 | 0 | 0.296 | 0.667 |
| hopB1 | hrpJ | 11125564 | 11125561 | 223283.PSPTO_1406 | 223283.PSPTO_1403 | 0.284 | 0 | 0.321 | 0 | 0 | 0 | 0 | 0.371 | 0.667 |
| iscU | erpA | 11125582 | 11124785 | 223283.PSPTO_1424 | 223283.PSPTO_0605 | 0.128 | 0 | 0 | 0 | 0.268 | 0 | 0 | 0.521 | 0.667 |
| cmaA | cfa5 | 11128778 | 11128758 | 223283.PSPTO_4709 | 223283.PSPTO_4685 | 0.044 | 0.001 | 0.653 | 0 | 0.061 | 0 | 0 | 0.059 | 0.667 |
| hopI1 | clpB | 11128837 | 11125009 | 223283.PSPTO_4776 | 223283.PSPTO_0829 | 0.056 | 0 | 0.305 | 0 | 0.266 | 0.362 | 0 | 0.082 | 0.666 |
| pchA | pvsA | 11126734 | 11126289 | 223283.PSPTO_2595 | 223283.PSPTO_2135 | 0.129 | 0 | 0 | 0 | 0.265 | 0.093 | 0 | 0.492 | 0.665 |
| cmaA | irp5 | 11128778 | 11126736 | 223283.PSPTO_4709 | 223283.PSPTO_2597 | 0.057 | 0 | 0.578 | 0.59 | 0.545 | 0 | 0 | 0.104 | 0.664 |
| PSPTO_2151 | PSPTO_2145 | 11126305 | 11126299 | 223283.PSPTO_2151 | 223283.PSPTO_2145 | 0.068 | 0 | 0.641 | 0 | 0.062 | 0 | 0 | 0.058 | 0.664 |
| cfa6 | iaaL | 11128759 | 11124556 | 223283.PSPTO_4686 | 223283.PSPTO_0371 | 0.056 | 0 | 0.652 | 0 | 0 | 0 | 0 | 0.062 | 0.664 |
| hrpA1 | hrpR | 11125539 | 11125537 | 223283.PSPTO_1381 | 223283.PSPTO_1379 | 0.485 | 0 | 0 | 0 | 0 | 0 | 0 | 0.371 | 0.662 |
| aceF | sdhB | 11129065 | 11126350 | 223283.PSPTO_5006 | 223283.PSPTO_2198 | 0.116 | 0 | 0 | 0 | 0.525 | 0.127 | 0 | 0.181 | 0.659 |
| hrpE | hrpZ1 | 11125544 | 11125540 | 223283.PSPTO_1386 | 223283.PSPTO_1382 | 0.645 | 0 | 0 | 0 | 0 | 0 | 0 | 0.079 | 0.659 |
| cfa6 | PSPTO_2134 | 11128759 | 11126288 | 223283.PSPTO_4686 | 223283.PSPTO_2134 | 0.068 | 0.212 | 0.554 | 0 | 0 | 0 | 0 | 0.083 | 0.659 |
| cfa9 | cfa5 | 11128763 | 11128758 | 223283.PSPTO_4690 | 223283.PSPTO_4685 | 0.545 | 0 | 0.248 | 0 | 0.052 | 0 | 0 | 0.064 | 0.656 |
| cfa6 | PSPTO_2147 | 11128759 | 11126301 | 223283.PSPTO_4686 | 223283.PSPTO_2147 | 0.056 | 0.177 | 0.569 | 0 | 0.06 | 0 | 0 | 0.075 | 0.656 |
| cmaA | iaaL | 11128778 | 11124556 | 223283.PSPTO_4709 | 223283.PSPTO_0371 | 0.053 | 0 | 0.468 | 0 | 0 | 0 | 0 | 0.37 | 0.654 |
| cfa2 | cfl | 11128755 | 11128753 | 223283.PSPTO_4682 | 223283.PSPTO_4680 | 0.587 | 0 | 0.167 | 0 | 0 | 0 | 0 | 0.072 | 0.654 |
| hrpZ1 | hrpS | 11125540 | 11125538 | 223283.PSPTO_1382 | 223283.PSPTO_1380 | 0.467 | 0 | 0 | 0 | 0 | 0 | 0 | 0.375 | 0.653 |
| aceE-2 | sucB | 11129064 | 11126352 | 223283.PSPTO_5005 | 223283.PSPTO_2200 | 0.068 | 0 | 0 | 0 | 0.294 | 0.467 | 0 | 0.122 | 0.65 |
| aceE-1 | sucB | 11127959 | 11126352 | 223283.PSPTO_3860 | 223283.PSPTO_2200 | 0.068 | 0 | 0 | 0 | 0.294 | 0.467 | 0 | 0.122 | 0.65 |
| cmaB | cfa7 | 11128779 | 11128760 | 223283.PSPTO_4710 | 223283.PSPTO_4687 | 0.053 | 0 | 0.634 | 0 | 0.051 | 0 | 0 | 0.064 | 0.65 |
| clpB | rpoH | 11125009 | 11124614 | 223283.PSPTO_0829 | 223283.PSPTO_0430 | 0.044 | 0 | 0 | 0 | 0.063 | 0.365 | 0 | 0.458 | 0.65 |
| cfa1 | cfl | 11128754 | 11128753 | 223283.PSPTO_4681 | 223283.PSPTO_4680 | 0.612 | 0 | 0 | 0 | 0 | 0.101 | 0 | 0.075 | 0.649 |
| acs | pgi | 11125981 | 11125130 | 223283.PSPTO_1825 | 223283.PSPTO_0959 | 0.352 | 0 | 0 | 0 | 0.083 | 0 | 0 | 0.457 | 0.649 |
| hrpE | hrpS | 11125544 | 11125538 | 223283.PSPTO_1386 | 223283.PSPTO_1380 | 0.383 | 0 | 0.166 | 0 | 0 | 0 | 0 | 0.373 | 0.649 |
| PSPTO_2605 | irp4 | 11126744 | 11126737 | 223283.PSPTO_2605 | 223283.PSPTO_2598 | 0.416 | 0 | 0.289 | 0 | 0.121 | 0 | 0 | 0.15 | 0.648 |
| gltA | rpoB | 11126346 | 11124799 | 223283.PSPTO_2194 | 223283.PSPTO_0619 | 0 | 0 | 0 | 0 | 0 | 0 | 0 | 0.648 | 0.648 |
| hrcQa | hrpW1 | 11125555 | 11125531 | 223283.PSPTO_1397 | 223283.PSPTO_1373 | 0 | 0 | 0.624 | 0 | 0 | 0 | 0 | 0.098 | 0.646 |
| hrcC | hrpZ1 | 11125547 | 11125540 | 223283.PSPTO_1389 | 223283.PSPTO_1382 | 0.455 | 0 | 0 | 0 | 0 | 0 | 0 | 0.375 | 0.645 |
| hrpL | hrcC | 11125562 | 11125547 | 223283.PSPTO_1404 | 223283.PSPTO_1389 | 0.056 | 0 | 0.429 | 0 | 0 | 0 | 0 | 0.395 | 0.645 |
| cfa1 | irp1 | 11128754 | 11126739 | 223283.PSPTO_4681 | 223283.PSPTO_2600 | 0.226 | 0 | 0 | 0 | 0.18 | 0.093 | 0 | 0.457 | 0.645 |
| hrpV | hrpZ1 | 11125549 | 11125540 | 223283.PSPTO_1391 | 223283.PSPTO_1382 | 0.455 | 0 | 0 | 0 | 0 | 0 | 0 | 0.373 | 0.644 |
| hrpT | hrpZ1 | 11125548 | 11125540 | 223283.PSPTO_1390 | 223283.PSPTO_1382 | 0.455 | 0 | 0 | 0 | 0 | 0 | 0 | 0.373 | 0.644 |
| hrcQb | hrpW1 | 11125554 | 11125531 | 223283.PSPTO_1396 | 223283.PSPTO_1373 | 0 | 0 | 0.621 | 0 | 0 | 0 | 0 | 0.097 | 0.643 |
| hrpG | hrpZ1 | 11125546 | 11125540 | 223283.PSPTO_1388 | 223283.PSPTO_1382 | 0.455 | 0 | 0 | 0 | 0 | 0 | 0 | 0.371 | 0.642 |
| cmaC | cfl | 11128780 | 11128753 | 223283.PSPTO_4711 | 223283.PSPTO_4680 | 0 | 0 | 0.641 | 0 | 0 | 0 | 0 | 0 | 0.641 |
| PSPTO_2152 | PSPTO_2145 | 11126306 | 11126299 | 223283.PSPTO_2152 | 223283.PSPTO_2145 | 0.068 | 0 | 0.616 | 0 | 0.062 | 0 | 0 | 0.058 | 0.641 |
| irp1 | pvsA | 11126739 | 11126289 | 223283.PSPTO_2600 | 223283.PSPTO_2135 | 0.393 | 0.005 | 0.572 | 0.578 | 0.085 | 0 | 0 | 0.461 | 0.638 |
| cfa7 | PSPTO_2149 | 11128760 | 11126303 | 223283.PSPTO_4687 | 223283.PSPTO_2149 | 0.056 | 0.154 | 0.559 | 0 | 0.06 | 0 | 0 | 0.075 | 0.638 |
| cfa7 | iaaL | 11128760 | 11124556 | 223283.PSPTO_4687 | 223283.PSPTO_0371 | 0.056 | 0 | 0.613 | 0 | 0 | 0 | 0 | 0.062 | 0.627 |
| cfa7 | irp4 | 11128760 | 11126737 | 223283.PSPTO_4687 | 223283.PSPTO_2598 | 0.068 | 0.033 | 0.598 | 0 | 0 | 0 | 0 | 0.083 | 0.626 |
| cmaU | iaaL | 11128783 | 11124556 | 223283.PSPTO_4714 | 223283.PSPTO_0371 | 0.054 | 0 | 0.61 | 0 | 0 | 0 | 0 | 0.065 | 0.624 |
| cfa9 | PSPTO_2146 | 11128763 | 11126300 | 223283.PSPTO_4690 | 223283.PSPTO_2146 | 0.084 | 0 | 0.603 | 0 | 0.05 | 0 | 0 | 0 | 0.624 |
| cfa8 | cfl | 11128762 | 11128753 | 223283.PSPTO_4689 | 223283.PSPTO_4680 | 0.478 | 0 | 0.261 | 0 | 0 | 0 | 0 | 0.101 | 0.623 |
| acs | rpoB | 11125981 | 11124799 | 223283.PSPTO_1825 | 223283.PSPTO_0619 | 0 | 0 | 0 | 0 | 0 | 0 | 0 | 0.622 | 0.622 |
| gpmA | pyk | 11129383 | 11128419 | 223283.PSPTO_5327 | 223283.PSPTO_4337 | 0.128 | 0 | 0 | 0 | 0.265 | 0 | 0 | 0.457 | 0.621 |
| corR | hopAQ1 | 11128773 | 11128772 | 223283.PSPTO_4704 | 223283.PSPTO_4703 | 0.509 | 0 | 0 | 0 | 0 | 0 | 0 | 0.261 | 0.621 |
| irp4 | PSPTO_2146 | 11126737 | 11126300 | 223283.PSPTO_2598 | 223283.PSPTO_2146 | 0.084 | 0 | 0.593 | 0 | 0.05 | 0 | 0 | 0 | 0.614 |
| cmaT | iaaL | 11128781 | 11124556 | 223283.PSPTO_4712 | 223283.PSPTO_0371 | 0 | 0 | 0.39 | 0 | 0 | 0 | 0 | 0.394 | 0.614 |
| pvdE | PSPTO_2151 | 11126307 | 11126305 | 223283.PSPTO_2153 | 223283.PSPTO_2151 | 0.279 | 0 | 0.192 | 0 | 0 | 0 | 0 | 0.386 | 0.611 |
| hrpL | hrpS | 11125562 | 11125538 | 223283.PSPTO_1404 | 223283.PSPTO_1380 | 0 | 0 | 0.177 | 0 | 0 | 0 | 0 | 0.544 | 0.609 |
| cmaC | cfa6 | 11128780 | 11128759 | 223283.PSPTO_4711 | 223283.PSPTO_4686 | 0 | 0 | 0.607 | 0 | 0 | 0 | 0 | 0 | 0.607 |
| fbp | tpiA | 11129224 | 11128574 | 223283.PSPTO_5168 | 223283.PSPTO_4494 | 0.125 | 0 | 0 | 0 | 0.103 | 0 | 0 | 0.538 | 0.605 |
| hrpZ1 | hrpR | 11125540 | 11125537 | 223283.PSPTO_1382 | 223283.PSPTO_1379 | 0.395 | 0 | 0 | 0 | 0 | 0 | 0 | 0.373 | 0.604 |
| cfa9 | pvdE | 11128763 | 11126307 | 223283.PSPTO_4690 | 223283.PSPTO_2153 | 0.084 | 0 | 0.584 | 0 | 0 | 0 | 0 | 0 | 0.602 |
| acnB | PSPTO_2196 | 11127851 | 11126348 | 223283.PSPTO_3752 | 223283.PSPTO_2196 | 0.073 | 0 | 0 | 0 | 0.264 | 0 | 0 | 0.461 | 0.6 |
| PSPTO_2605 | pchA | 11126744 | 11126734 | 223283.PSPTO_2605 | 223283.PSPTO_2595 | 0.39 | 0 | 0.232 | 0 | 0.198 | 0 | 0 | 0.055 | 0.598 |
| acnB | sdhA | 11127851 | 11126349 | 223283.PSPTO_3752 | 223283.PSPTO_2197 | 0.071 | 0 | 0 | 0 | 0.265 | 0 | 0 | 0.457 | 0.596 |
| hopI1 | clpA | 11128837 | 11127463 | 223283.PSPTO_4776 | 223283.PSPTO_3353 | 0.056 | 0 | 0.156 | 0 | 0.266 | 0.362 | 0 | 0.082 | 0.595 |
| hrpK1 | hrpL | 11125563 | 11125562 | 223283.PSPTO_1405 | 223283.PSPTO_1404 | 0.595 | 0 | 0 | 0 | 0 | 0 | 0 | 0 | 0.595 |
| PSPTO_4339 | sucB | 11128421 | 11126352 | 223283.PSPTO_4339 | 223283.PSPTO_2200 | 0.069 | 0 | 0 | 0 | 0.137 | 0 | 0 | 0.535 | 0.593 |
| rpoN | rpoH | 11128533 | 11124614 | 223283.PSPTO_4453 | 223283.PSPTO_0430 | 0 | 0 | 0 | 0 | 0 | 0 | 0 | 0.589 | 0.589 |
| hrpV | hrpA1 | 11125549 | 11125539 | 223283.PSPTO_1391 | 223283.PSPTO_1381 | 0.373 | 0 | 0 | 0 | 0 | 0 | 0 | 0.371 | 0.588 |
| cfa4 | cfl | 11128757 | 11128753 | 223283.PSPTO_4684 | 223283.PSPTO_4680 | 0.585 | 0 | 0 | 0 | 0 | 0 | 0 | 0 | 0.585 |
| glk | gap-1 | 11125449 | 11125447 | 223283.PSPTO_1289 | 223283.PSPTO_1287 | 0.527 | 0 | 0 | 0 | 0 | 0 | 0 | 0.158 | 0.585 |
| hscB | erpA | 11125584 | 11124785 | 223283.PSPTO_1426 | 223283.PSPTO_0605 | 0.129 | 0 | 0 | 0 | 0.142 | 0.093 | 0 | 0.461 | 0.585 |
| PSPTO_4338 | sdhA | 11128420 | 11126349 | 223283.PSPTO_4338 | 223283.PSPTO_2197 | 0.374 | 0 | 0 | 0 | 0.14 | 0.12 | 0 | 0.226 | 0.584 |
| nuoN | rpoD | 11127487 | 11124718 | 223283.PSPTO_3377 | 223283.PSPTO_0537 | 0 | 0 | 0 | 0 | 0 | 0 | 0 | 0.584 | 0.584 |
| aceF | PSPTO_4339 | 11129065 | 11128421 | 223283.PSPTO_5006 | 223283.PSPTO_4339 | 0.048 | 0 | 0 | 0 | 0.131 | 0 | 0 | 0.535 | 0.581 |
| hrcC | hrpW1 | 11125547 | 11125531 | 223283.PSPTO_1389 | 223283.PSPTO_1373 | 0 | 0 | 0.544 | 0 | 0 | 0 | 0 | 0.113 | 0.578 |
| hrpK1 | hrcV | 11125563 | 11125560 | 223283.PSPTO_1405 | 223283.PSPTO_1402 | 0.355 | 0 | 0 | 0 | 0 | 0 | 0 | 0.372 | 0.577 |
| cmaC | cfa7 | 11128780 | 11128760 | 223283.PSPTO_4711 | 223283.PSPTO_4687 | 0 | 0 | 0.575 | 0 | 0 | 0 | 0 | 0 | 0.575 |
| irp5 | pvsA | 11126736 | 11126289 | 223283.PSPTO_2597 | 223283.PSPTO_2135 | 0.086 | 0 | 0.649 | 0.605 | 0.371 | 0 | 0 | 0.173 | 0.575 |
| irp4 | pvdE | 11126737 | 11126307 | 223283.PSPTO_2598 | 223283.PSPTO_2153 | 0.084 | 0 | 0.553 | 0 | 0 | 0 | 0 | 0 | 0.573 |
| fbp | eno-1 | 11129224 | 11125711 | 223283.PSPTO_5168 | 223283.PSPTO_1554 | 0 | 0 | 0 | 0 | 0.127 | 0 | 0 | 0.532 | 0.573 |
| hrpK1 | hrpJ | 11125563 | 11125561 | 223283.PSPTO_1405 | 223283.PSPTO_1403 | 0.355 | 0 | 0 | 0 | 0 | 0 | 0 | 0.365 | 0.573 |
| cmaB | cfa5 | 11128779 | 11128758 | 223283.PSPTO_4710 | 223283.PSPTO_4685 | 0 | 0 | 0.554 | 0 | 0.062 | 0 | 0 | 0.059 | 0.571 |
| acnB | sucD | 11127851 | 11126355 | 223283.PSPTO_3752 | 223283.PSPTO_2203 | 0.126 | 0 | 0 | 0 | 0.268 | 0.094 | 0 | 0.344 | 0.568 |
| cfa6 | irp4 | 11128759 | 11126737 | 223283.PSPTO_4686 | 223283.PSPTO_2598 | 0.068 | 0.013 | 0.536 | 0 | 0 | 0 | 0 | 0.083 | 0.568 |
| cfa9 | cfa3 | 11128763 | 11128756 | 223283.PSPTO_4690 | 223283.PSPTO_4683 | 0.543 | 0.089 | 0 | 0 | 0 | 0 | 0 | 0 | 0.567 |
| cmaT | PSPTO_2146 | 11128781 | 11126300 | 223283.PSPTO_4712 | 223283.PSPTO_2146 | 0.056 | 0 | 0.554 | 0 | 0.05 | 0 | 0 | 0 | 0.565 |
| PSPTO_4338 | iscU | 11128420 | 11125582 | 223283.PSPTO_4338 | 223283.PSPTO_1424 | 0.126 | 0 | 0 | 0 | 0.118 | 0.416 | 0 | 0.143 | 0.562 |
| cfa7 | PSPTO_2602 | 11128760 | 11126741 | 223283.PSPTO_4687 | 223283.PSPTO_2602 | 0.056 | 0.231 | 0.411 | 0 | 0.06 | 0 | 0 | 0.075 | 0.56 |
| cfa9 | cfa1 | 11128763 | 11128754 | 223283.PSPTO_4690 | 223283.PSPTO_4681 | 0.535 | 0 | 0 | 0 | 0 | 0 | 0 | 0.082 | 0.556 |
| tpiA | sdhA | 11128574 | 11126349 | 223283.PSPTO_4494 | 223283.PSPTO_2197 | 0.127 | 0 | 0 | 0 | 0.134 | 0 | 0 | 0.457 | 0.553 |
| lpdA | PSPTO_2196 | 11126353 | 11126348 | 223283.PSPTO_2201 | 223283.PSPTO_2196 | 0.49 | 0 | 0 | 0 | 0.142 | 0 | 0 | 0.06 | 0.553 |
| cfa9 | cfa2 | 11128763 | 11128755 | 223283.PSPTO_4690 | 223283.PSPTO_4682 | 0.542 | 0 | 0 | 0 | 0 | 0 | 0 | 0.059 | 0.551 |
| PSPTO_4338 | sdhB | 11128420 | 11126350 | 223283.PSPTO_4338 | 223283.PSPTO_2198 | 0.378 | 0 | 0 | 0 | 0.237 | 0 | 0 | 0.128 | 0.55 |
| oadA | eno-2 | 11129558 | 11128692 | 223283.PSPTO_5510 | 223283.PSPTO_4616 | 0 | 0 | 0.161 | 0 | 0.06 | 0.365 | 0 | 0.206 | 0.549 |
| cmaT | pvdE | 11128781 | 11126307 | 223283.PSPTO_4712 | 223283.PSPTO_2153 | 0.079 | 0 | 0.53 | 0 | 0 | 0 | 0 | 0 | 0.548 |
| cfa9 | cfl | 11128763 | 11128753 | 223283.PSPTO_4690 | 223283.PSPTO_4680 | 0.397 | 0.008 | 0.255 | 0 | 0.052 | 0 | 0 | 0.064 | 0.548 |
| clpA | rpoS | 11127463 | 11125722 | 223283.PSPTO_3353 | 223283.PSPTO_1565 | 0.044 | 0 | 0.247 | 0 | 0.084 | 0.365 | 0 | 0.083 | 0.546 |
| oadA | eno-1 | 11129558 | 11125711 | 223283.PSPTO_5510 | 223283.PSPTO_1554 | 0 | 0 | 0.153 | 0 | 0.06 | 0.365 | 0 | 0.206 | 0.544 |
| rpoS | clpB | 11125722 | 11125009 | 223283.PSPTO_1565 | 223283.PSPTO_0829 | 0.044 | 0 | 0.164 | 0 | 0.052 | 0.365 | 0 | 0.197 | 0.543 |
| PSPTO_4339 | gap-1 | 11128421 | 11125447 | 223283.PSPTO_4339 | 223283.PSPTO_1287 | 0 | 0 | 0 | 0 | 0.187 | 0 | 0 | 0.461 | 0.543 |
| nuoN | rpoB | 11127487 | 11124799 | 223283.PSPTO_3377 | 223283.PSPTO_0619 | 0 | 0 | 0 | 0 | 0 | 0 | 0 | 0.541 | 0.541 |
| cmaA | PSPTO_2146 | 11128778 | 11126300 | 223283.PSPTO_4709 | 223283.PSPTO_2146 | 0.056 | 0.211 | 0.407 | 0 | 0.061 | 0 | 0 | 0.064 | 0.541 |
| hrcC | hrpS | 11125547 | 11125538 | 223283.PSPTO_1389 | 223283.PSPTO_1380 | 0.285 | 0 | 0 | 0 | 0 | 0 | 0 | 0.383 | 0.54 |
| pchA | PSPTO_2134 | 11126734 | 11126288 | 223283.PSPTO_2595 | 223283.PSPTO_2134 | 0.084 | 0.006 | 0 | 0 | 0.497 | 0 | 0 | 0.083 | 0.54 |
| cfa9 | pchA | 11128763 | 11126734 | 223283.PSPTO_4690 | 223283.PSPTO_2595 | 0.084 | 0.003 | 0 | 0 | 0.497 | 0 | 0 | 0.083 | 0.54 |
| tpiA | clpB | 11128574 | 11125009 | 223283.PSPTO_4494 | 223283.PSPTO_0829 | 0.062 | 0 | 0.48 | 0 | 0.06 | 0 | 0 | 0.115 | 0.539 |
| cfa9 | cfa4 | 11128763 | 11128757 | 223283.PSPTO_4690 | 223283.PSPTO_4684 | 0.539 | 0 | 0 | 0 | 0 | 0 | 0 | 0 | 0.539 |
| irp1 | pvdE | 11126739 | 11126307 | 223283.PSPTO_2600 | 223283.PSPTO_2153 | 0.297 | 0 | 0.282 | 0 | 0 | 0 | 0 | 0.16 | 0.538 |
| pvdE | PSPTO_2152 | 11126307 | 11126306 | 223283.PSPTO_2153 | 223283.PSPTO_2152 | 0.434 | 0 | 0.181 | 0 | 0 | 0 | 0 | 0.073 | 0.534 |
| hrpG | hrpS | 11125546 | 11125538 | 223283.PSPTO_1388 | 223283.PSPTO_1380 | 0.285 | 0 | 0 | 0 | 0 | 0 | 0 | 0.376 | 0.534 |
| PSPTO_4569 | tpiA | 11128647 | 11128574 | 223283.PSPTO_4569 | 223283.PSPTO_4494 | 0 | 0 | 0.154 | 0 | 0.059 | 0 | 0 | 0.46 | 0.532 |
| hrpT | hrpS | 11125548 | 11125538 | 223283.PSPTO_1390 | 223283.PSPTO_1380 | 0.285 | 0 | 0 | 0 | 0 | 0 | 0 | 0.371 | 0.531 |
| aceF | tilS | 11129065 | 11125708 | 223283.PSPTO_5006 | 223283.PSPTO_1551 | 0 | 0 | 0.169 | 0 | 0 | 0 | 0 | 0.458 | 0.53 |
| tpiA | sdhC | 11128574 | 11126347 | 223283.PSPTO_4494 | 223283.PSPTO_2195 | 0.094 | 0 | 0 | 0 | 0.123 | 0 | 0 | 0.457 | 0.53 |
| hrcV | hrpW1 | 11125560 | 11125531 | 223283.PSPTO_1402 | 223283.PSPTO_1373 | 0.056 | 0 | 0.218 | 0 | 0 | 0 | 0 | 0.414 | 0.529 |
| acnB | sucB | 11127851 | 11126352 | 223283.PSPTO_3752 | 223283.PSPTO_2200 | 0.057 | 0 | 0 | 0 | 0.302 | 0 | 0 | 0.341 | 0.528 |
| PSPTO_2289 | sdhA | 11126439 | 11126349 | 223283.PSPTO_2289 | 223283.PSPTO_2197 | 0.056 | 0 | 0 | 0 | 0.415 | 0 | 0 | 0.215 | 0.528 |
| irp3 | PSPTO_2149 | 11126738 | 11126303 | 223283.PSPTO_2599 | 223283.PSPTO_2149 | 0.056 | 0 | 0.473 | 0 | 0.091 | 0 | 0 | 0.078 | 0.527 |
| sdhA | acs | 11126349 | 11125981 | 223283.PSPTO_2197 | 223283.PSPTO_1825 | 0.076 | 0 | 0.308 | 0 | 0.142 | 0 | 0 | 0.24 | 0.527 |
| irp3 | PSPTO_2148 | 11126738 | 11126302 | 223283.PSPTO_2599 | 223283.PSPTO_2148 | 0.056 | 0 | 0.471 | 0 | 0.091 | 0 | 0 | 0.078 | 0.525 |
| acnB | sdhB | 11127851 | 11126350 | 223283.PSPTO_3752 | 223283.PSPTO_2198 | 0.056 | 0 | 0 | 0 | 0.239 | 0.093 | 0 | 0.354 | 0.522 |
| sdhA | eno-1 | 11126349 | 11125711 | 223283.PSPTO_2197 | 223283.PSPTO_1554 | 0 | 0 | 0 | 0 | 0.101 | 0.093 | 0 | 0.461 | 0.522 |
| hopB1 | hrcV | 11125564 | 11125560 | 223283.PSPTO_1406 | 223283.PSPTO_1402 | 0.284 | 0 | 0 | 0 | 0 | 0 | 0 | 0.357 | 0.52 |
| PSPTO_2605 | PSPTO_2151 | 11126744 | 11126305 | 223283.PSPTO_2605 | 223283.PSPTO_2151 | 0 | 0 | 0.457 | 0 | 0.133 | 0 | 0 | 0.064 | 0.52 |
| eno-1 | tilS | 11125711 | 11125708 | 223283.PSPTO_1554 | 223283.PSPTO_1551 | 0.518 | 0 | 0 | 0 | 0 | 0 | 0 | 0 | 0.518 |
| oadA | tpiA | 11129558 | 11128574 | 223283.PSPTO_5510 | 223283.PSPTO_4494 | 0.046 | 0 | 0 | 0 | 0.138 | 0 | 0 | 0.46 | 0.517 |
| cfa7 | pvsA | 11128760 | 11126289 | 223283.PSPTO_4687 | 223283.PSPTO_2135 | 0.123 | 0.029 | 0.398 | 0 | 0.06 | 0 | 0 | 0.143 | 0.517 |
| PSPTO_5465 | gltA | 11129513 | 11126346 | 223283.PSPTO_5465 | 223283.PSPTO_2194 | 0.051 | 0 | 0 | 0 | 0.159 | 0.312 | 0 | 0.222 | 0.515 |
| hrpE | hrpA1 | 11125544 | 11125539 | 223283.PSPTO_1386 | 223283.PSPTO_1381 | 0.514 | 0 | 0 | 0 | 0 | 0 | 0 | 0 | 0.514 |
| hrpV | hrpR | 11125549 | 11125537 | 223283.PSPTO_1391 | 223283.PSPTO_1379 | 0.252 | 0 | 0 | 0 | 0 | 0 | 0 | 0.376 | 0.513 |
| hrpG | hrpR | 11125546 | 11125537 | 223283.PSPTO_1388 | 223283.PSPTO_1379 | 0.252 | 0 | 0 | 0 | 0 | 0 | 0 | 0.375 | 0.512 |
| cfa7 | PSPTO_2148 | 11128760 | 11126302 | 223283.PSPTO_4687 | 223283.PSPTO_2148 | 0.056 | 0.069 | 0.451 | 0 | 0.06 | 0 | 0 | 0.075 | 0.504 |
| oadA | sucD | 11129558 | 11126355 | 223283.PSPTO_5510 | 223283.PSPTO_2203 | 0.384 | 0 | 0 | 0 | 0.064 | 0 | 0 | 0.206 | 0.502 |
| cfa6 | PSPTO_2149 | 11128759 | 11126303 | 223283.PSPTO_4686 | 223283.PSPTO_2149 | 0.056 | 0.052 | 0.459 | 0 | 0.06 | 0 | 0 | 0.075 | 0.502 |
| fbp | pyk | 11129224 | 11128419 | 223283.PSPTO_5168 | 223283.PSPTO_4337 | 0.058 | 0 | 0 | 0 | 0.104 | 0 | 0 | 0.457 | 0.501 |
| sdhB | cyaY | 11126350 | 11124414 | 223283.PSPTO_2198 | 223283.PSPTO_0227 | 0.056 | 0 | 0 | 0 | 0.141 | 0.406 | 0 | 0.085 | 0.5 |
| irp3 | PSPTO_2150 | 11126738 | 11126304 | 223283.PSPTO_2599 | 223283.PSPTO_2150 | 0.056 | 0 | 0.44 | 0 | 0.091 | 0 | 0 | 0.078 | 0.497 |
| lpdA | gltA | 11126353 | 11126346 | 223283.PSPTO_2201 | 223283.PSPTO_2194 | 0.236 | 0 | 0 | 0 | 0.114 | 0.095 | 0 | 0.274 | 0.496 |
| gpmA | rpoC | 11129383 | 11124800 | 223283.PSPTO_5327 | 223283.PSPTO_0620 | 0 | 0 | 0 | 0 | 0.233 | 0.358 | 0 | 0.058 | 0.495 |
| tpiA | gltA | 11128574 | 11126346 | 223283.PSPTO_4494 | 223283.PSPTO_2194 | 0.06 | 0 | 0 | 0 | 0.084 | 0 | 0 | 0.461 | 0.495 |
| PSPTO_1207 | fecC | 11125370 | 11124942 | 223283.PSPTO_1207 | 223283.PSPTO_0762 | 0.124 | 0 | 0.19 | 0 | 0.263 | 0 | 0 | 0.148 | 0.494 |
| cfa1 | sdhC | 11128754 | 11126347 | 223283.PSPTO_4681 | 223283.PSPTO_2195 | 0 | 0 | 0 | 0 | 0.494 | 0 | 0 | 0 | 0.493 |
| PSPTO_3256 | PSPTO_2152 | 11127366 | 11126306 | 223283.PSPTO_3256 | 223283.PSPTO_2152 | 0.056 | 0 | 0.166 | 0 | 0.057 | 0 | 0 | 0.398 | 0.493 |
| oadA | sdhA | 11129558 | 11126349 | 223283.PSPTO_5510 | 223283.PSPTO_2197 | 0.078 | 0 | 0 | 0 | 0.062 | 0 | 0 | 0.46 | 0.492 |
| cmaA | irp1 | 11128778 | 11126739 | 223283.PSPTO_4709 | 223283.PSPTO_2600 | 0.068 | 0.174 | 0.336 | 0 | 0.061 | 0 | 0 | 0.104 | 0.492 |
| PSPTO_4338 | iscA | 11128420 | 11125583 | 223283.PSPTO_4338 | 223283.PSPTO_1425 | 0.083 | 0 | 0 | 0 | 0.141 | 0.381 | 0 | 0.078 | 0.49 |
| PSPTO_4338 | erpA | 11128420 | 11124785 | 223283.PSPTO_4338 | 223283.PSPTO_0605 | 0.083 | 0 | 0 | 0 | 0.141 | 0.381 | 0 | 0.078 | 0.49 |
| eno-1 | erpA | 11125711 | 11124785 | 223283.PSPTO_1554 | 223283.PSPTO_0605 | 0 | 0 | 0 | 0 | 0.091 | 0 | 0 | 0.461 | 0.489 |
| irp1 | PSPTO_2147 | 11126739 | 11126301 | 223283.PSPTO_2600 | 223283.PSPTO_2147 | 0.068 | 0.233 | 0.677 | 0.578 | 0.061 | 0 | 0 | 0.104 | 0.489 |
| pgi | argH | 11125130 | 11124321 | 223283.PSPTO_0959 | 223283.PSPTO_0125 | 0.086 | 0 | 0 | 0 | 0.052 | 0 | 0 | 0.457 | 0.488 |
| acnB | rpoD | 11127851 | 11124718 | 223283.PSPTO_3752 | 223283.PSPTO_0537 | 0 | 0 | 0 | 0 | 0 | 0 | 0 | 0.487 | 0.487 |
| clpB | rpoD | 11125009 | 11124718 | 223283.PSPTO_0829 | 223283.PSPTO_0537 | 0.044 | 0 | 0 | 0 | 0.066 | 0.365 | 0 | 0.2 | 0.486 |
| cfa7 | irp5 | 11128760 | 11126736 | 223283.PSPTO_4687 | 223283.PSPTO_2597 | 0.084 | 0.065 | 0.41 | 0 | 0 | 0 | 0 | 0.104 | 0.486 |
| sucB | erpA | 11126352 | 11124785 | 223283.PSPTO_2200 | 223283.PSPTO_0605 | 0 | 0 | 0.477 | 0 | 0 | 0 | 0 | 0.059 | 0.486 |
| PSPTO_4339 | lpdA | 11128421 | 11126353 | 223283.PSPTO_4339 | 223283.PSPTO_2201 | 0 | 0 | 0 | 0 | 0.091 | 0 | 0 | 0.458 | 0.486 |
| hrpL | hrcU | 11125562 | 11125550 | 223283.PSPTO_1404 | 223283.PSPTO_1392 | 0.361 | 0 | 0.219 | 0 | 0 | 0 | 0 | 0.052 | 0.485 |
| PSPTO_2152 | PSPTO_1207 | 11126306 | 11125370 | 223283.PSPTO_2152 | 223283.PSPTO_1207 | 0 | 0 | 0.378 | 0 | 0 | 0.093 | 0 | 0.16 | 0.484 |
| lpdA | eno-1 | 11126353 | 11125711 | 223283.PSPTO_2201 | 223283.PSPTO_1554 | 0.086 | 0 | 0.236 | 0 | 0.185 | 0.096 | 0 | 0.153 | 0.484 |
| tpiA | rpoB | 11128574 | 11124799 | 223283.PSPTO_4494 | 223283.PSPTO_0619 | 0.073 | 0 | 0 | 0 | 0.052 | 0 | 0 | 0.459 | 0.483 |
| irp3 | pvsA | 11126738 | 11126289 | 223283.PSPTO_2599 | 223283.PSPTO_2135 | 0.125 | 0 | 0.337 | 0 | 0.141 | 0 | 0 | 0.08 | 0.48 |
| cfa3 | iaaL | 11128756 | 11124556 | 223283.PSPTO_4683 | 223283.PSPTO_0371 | 0.056 | 0 | 0.471 | 0 | 0 | 0 | 0 | 0 | 0.479 |
| pgi | rpoC | 11125130 | 11124800 | 223283.PSPTO_0959 | 223283.PSPTO_0620 | 0 | 0 | 0 | 0 | 0.061 | 0 | 0 | 0.467 | 0.478 |
| gap-1 | rpoD | 11125447 | 11124718 | 223283.PSPTO_1287 | 223283.PSPTO_0537 | 0.052 | 0 | 0 | 0 | 0.052 | 0.097 | 0 | 0.433 | 0.478 |
| tpiA | algC | 11128574 | 11124280 | 223283.PSPTO_4494 | 223283.PSPTO_0083 | 0.115 | 0 | 0 | 0 | 0.06 | 0.323 | 0 | 0.181 | 0.477 |
| PSPTO_4339 | pyk | 11128421 | 11128419 | 223283.PSPTO_4339 | 223283.PSPTO_4337 | 0.071 | 0 | 0 | 0 | 0 | 0 | 0 | 0.461 | 0.477 |
| corR | hrpL | 11128773 | 11125562 | 223283.PSPTO_4704 | 223283.PSPTO_1404 | 0 | 0 | 0.184 | 0 | 0 | 0 | 0 | 0.384 | 0.476 |
| cfa7 | PSPTO_2150 | 11128760 | 11126304 | 223283.PSPTO_4687 | 223283.PSPTO_2150 | 0.056 | 0.061 | 0.425 | 0 | 0.06 | 0 | 0 | 0.075 | 0.476 |
| clpA | rpoH | 11127463 | 11124614 | 223283.PSPTO_3353 | 223283.PSPTO_0430 | 0.044 | 0 | 0 | 0 | 0.052 | 0.365 | 0 | 0.196 | 0.475 |
| corR | rpoS | 11128773 | 11125722 | 223283.PSPTO_4704 | 223283.PSPTO_1565 | 0 | 0 | 0 | 0 | 0 | 0.174 | 0 | 0.39 | 0.475 |
| sdhB | acs | 11126350 | 11125981 | 223283.PSPTO_2198 | 223283.PSPTO_1825 | 0.056 | 0 | 0.295 | 0 | 0.114 | 0 | 0 | 0.215 | 0.475 |
| pgi | rpoB | 11125130 | 11124799 | 223283.PSPTO_0959 | 223283.PSPTO_0619 | 0 | 0 | 0 | 0 | 0.061 | 0 | 0 | 0.463 | 0.474 |
| gltA | rpoA | 11126346 | 11124831 | 223283.PSPTO_2194 | 223283.PSPTO_0651 | 0.076 | 0 | 0 | 0 | 0.052 | 0 | 0 | 0.448 | 0.474 |
| cmaB | cfl | 11128779 | 11128753 | 223283.PSPTO_4710 | 223283.PSPTO_4680 | 0 | 0 | 0.452 | 0 | 0.062 | 0 | 0 | 0.059 | 0.474 |
| gltA | gap-1 | 11126346 | 11125447 | 223283.PSPTO_2194 | 223283.PSPTO_1287 | 0.049 | 0 | 0 | 0 | 0.063 | 0 | 0 | 0.456 | 0.473 |
| eno-2 | PSPTO_4339 | 11128692 | 11128421 | 223283.PSPTO_4616 | 223283.PSPTO_4339 | 0 | 0 | 0 | 0 | 0.061 | 0 | 0 | 0.461 | 0.472 |
| fbp | acs | 11129224 | 11125981 | 223283.PSPTO_5168 | 223283.PSPTO_1825 | 0 | 0 | 0 | 0 | 0.352 | 0 | 0 | 0.22 | 0.472 |
| PSPTO_4339 | eno-1 | 11128421 | 11125711 | 223283.PSPTO_4339 | 223283.PSPTO_1554 | 0 | 0 | 0 | 0 | 0.061 | 0 | 0 | 0.461 | 0.472 |
| rpoD | rpoH | 11124718 | 11124614 | 223283.PSPTO_0537 | 223283.PSPTO_0430 | 0 | 0 | 0.758 | 0.627 | 0.058 | 0 | 0 | 0.606 | 0.47 |
| acnB | lpdA | 11127851 | 11126353 | 223283.PSPTO_3752 | 223283.PSPTO_2201 | 0.083 | 0.117 | 0 | 0 | 0.1 | 0 | 0 | 0.358 | 0.469 |
| cmaA | pvdE | 11128778 | 11126307 | 223283.PSPTO_4709 | 223283.PSPTO_2153 | 0.056 | 0 | 0.441 | 0 | 0 | 0 | 0 | 0.076 | 0.469 |
| PSPTO_4339 | pgi | 11128421 | 11125130 | 223283.PSPTO_4339 | 223283.PSPTO_0959 | 0.054 | 0 | 0 | 0 | 0 | 0 | 0 | 0.461 | 0.468 |
| cmaA | cfa3 | 11128778 | 11128756 | 223283.PSPTO_4709 | 223283.PSPTO_4683 | 0 | 0.276 | 0.281 | 0 | 0 | 0 | 0 | 0.059 | 0.467 |
| PSPTO_4339 | rpoA | 11128421 | 11124831 | 223283.PSPTO_4339 | 223283.PSPTO_0651 | 0 | 0 | 0 | 0 | 0.057 | 0 | 0 | 0.458 | 0.467 |
| tpiA | lpdA | 11128574 | 11126353 | 223283.PSPTO_4494 | 223283.PSPTO_2201 | 0.125 | 0 | 0 | 0 | 0.114 | 0 | 0 | 0.367 | 0.466 |
| cfa6 | hrcC | 11128759 | 11125547 | 223283.PSPTO_4686 | 223283.PSPTO_1389 | 0 | 0 | 0.185 | 0 | 0 | 0 | 0 | 0.371 | 0.465 |
| cfa1 | nuoN | 11128754 | 11127487 | 223283.PSPTO_4681 | 223283.PSPTO_3377 | 0 | 0 | 0 | 0 | 0 | 0.455 | 0 | 0.06 | 0.465 |
| PSPTO_2152 | PSPTO_2151 | 11126306 | 11126305 | 223283.PSPTO_2152 | 223283.PSPTO_2151 | 0.434 | 0 | 0.785 | 0.953 | 0 | 0 | 0 | 0.373 | 0.463 |
| tpiA | acs | 11128574 | 11125981 | 223283.PSPTO_4494 | 223283.PSPTO_1825 | 0 | 0 | 0 | 0 | 0.052 | 0 | 0 | 0.457 | 0.463 |
| PSPTO_2289 | sucD | 11126439 | 11126355 | 223283.PSPTO_2289 | 223283.PSPTO_2203 | 0.086 | 0 | 0 | 0 | 0.268 | 0.126 | 0 | 0.188 | 0.461 |
| tpiA | PSPTO_4339 | 11128574 | 11128421 | 223283.PSPTO_4494 | 223283.PSPTO_4339 | 0 | 0 | 0 | 0 | 0 | 0 | 0 | 0.461 | 0.461 |
| PSPTO_5343 | cyaY | 11129399 | 11124414 | 223283.PSPTO_5343 | 223283.PSPTO_0227 | 0 | 0 | 0 | 0 | 0 | 0 | 0 | 0.459 | 0.459 |
| acnB | gap-1 | 11127851 | 11125447 | 223283.PSPTO_3752 | 223283.PSPTO_1287 | 0 | 0 | 0 | 0 | 0.052 | 0 | 0 | 0.453 | 0.459 |
| pyk | rpoD | 11128419 | 11124718 | 223283.PSPTO_4337 | 223283.PSPTO_0537 | 0 | 0 | 0 | 0 | 0 | 0 | 0 | 0.46 | 0.459 |
| tpiA | sdhB | 11128574 | 11126350 | 223283.PSPTO_4494 | 223283.PSPTO_2198 | 0.126 | 0 | 0 | 0 | 0.096 | 0 | 0 | 0.371 | 0.459 |
| hrpJ | hrpS | 11125561 | 11125538 | 223283.PSPTO_1403 | 223283.PSPTO_1380 | 0 | 0 | 0.169 | 0 | 0 | 0 | 0 | 0.375 | 0.458 |
| irp1 | cyoE | 11126739 | 11125487 | 223283.PSPTO_2600 | 223283.PSPTO_1329 | 0 | 0 | 0 | 0 | 0 | 0 | 0 | 0.458 | 0.457 |
| cyoE | rpoB | 11125487 | 11124799 | 223283.PSPTO_1329 | 223283.PSPTO_0619 | 0 | 0 | 0 | 0 | 0 | 0 | 0 | 0.457 | 0.457 |
| PSPTO_2152 | PSPTO_0268 | 11126306 | 11124455 | 223283.PSPTO_2152 | 223283.PSPTO_0268 | 0 | 0 | 0.377 | 0 | 0.133 | 0 | 0 | 0.076 | 0.457 |
| cfa1 | purB | 11128754 | 11127470 | 223283.PSPTO_4681 | 223283.PSPTO_3360 | 0 | 0 | 0 | 0 | 0.128 | 0.403 | 0 | 0 | 0.457 |
| PSPTO_4338 | hscB | 11128420 | 11125584 | 223283.PSPTO_4338 | 223283.PSPTO_1426 | 0.075 | 0 | 0.169 | 0 | 0.264 | 0.093 | 0 | 0.104 | 0.456 |
| cfa6 | pvsA | 11128759 | 11126289 | 223283.PSPTO_4686 | 223283.PSPTO_2135 | 0.123 | 0.013 | 0.321 | 0 | 0.06 | 0 | 0 | 0.143 | 0.456 |
| hopB1 | hrcQa | 11125564 | 11125555 | 223283.PSPTO_1406 | 223283.PSPTO_1397 | 0.262 | 0 | 0.294 | 0 | 0 | 0 | 0 | 0 | 0.456 |
| pgi | rpoD | 11125130 | 11124718 | 223283.PSPTO_0959 | 223283.PSPTO_0537 | 0.056 | 0 | 0 | 0 | 0 | 0 | 0 | 0.444 | 0.452 |
| PSPTO_2289 | sucB | 11126439 | 11126352 | 223283.PSPTO_2289 | 223283.PSPTO_2200 | 0.065 | 0 | 0.199 | 0 | 0.234 | 0 | 0 | 0.156 | 0.451 |
| hrpZ1 | hrpW1 | 11125540 | 11125531 | 223283.PSPTO_1382 | 223283.PSPTO_1373 | 0 | 0 | 0 | 0 | 0 | 0 | 0 | 0.451 | 0.451 |
| sdhA | cyaY | 11126349 | 11124414 | 223283.PSPTO_2197 | 223283.PSPTO_0227 | 0.057 | 0 | 0 | 0 | 0.061 | 0.386 | 0 | 0.106 | 0.448 |
| pyk | glk | 11128419 | 11125449 | 223283.PSPTO_4337 | 223283.PSPTO_1289 | 0.312 | 0 | 0 | 0 | 0 | 0 | 0 | 0.231 | 0.448 |
| hopB1 | hrpL | 11125564 | 11125562 | 223283.PSPTO_1406 | 223283.PSPTO_1404 | 0.448 | 0 | 0 | 0 | 0 | 0 | 0 | 0 | 0.448 |
| sucD | eno-1 | 11126355 | 11125711 | 223283.PSPTO_2203 | 223283.PSPTO_1554 | 0 | 0 | 0 | 0 | 0.366 | 0 | 0 | 0.164 | 0.447 |
| clpA | rpoD | 11127463 | 11124718 | 223283.PSPTO_3353 | 223283.PSPTO_0537 | 0.044 | 0 | 0 | 0 | 0.052 | 0.365 | 0 | 0.154 | 0.447 |
| eno-2 | sucD | 11128692 | 11126355 | 223283.PSPTO_4616 | 223283.PSPTO_2203 | 0 | 0 | 0 | 0 | 0.366 | 0 | 0 | 0.164 | 0.447 |
| PSPTO_4569 | gap-1 | 11128647 | 11125447 | 223283.PSPTO_4569 | 223283.PSPTO_1287 | 0 | 0 | 0 | 0 | 0.088 | 0.388 | 0 | 0.088 | 0.446 |
| PSPTO_1207 | PSPTO_0268 | 11125370 | 11124455 | 223283.PSPTO_1207 | 223283.PSPTO_0268 | 0 | 0 | 0.369 | 0 | 0.126 | 0 | 0 | 0.077 | 0.446 |
| corR | iaaL | 11128773 | 11124556 | 223283.PSPTO_4704 | 223283.PSPTO_0371 | 0 | 0 | 0.153 | 0 | 0 | 0 | 0 | 0.37 | 0.443 |
| aceF | sdhC | 11129065 | 11126347 | 223283.PSPTO_5006 | 223283.PSPTO_2195 | 0.116 | 0 | 0 | 0 | 0.287 | 0 | 0 | 0.188 | 0.443 |
| hrpL | hrcR | 11125562 | 11125553 | 223283.PSPTO_1404 | 223283.PSPTO_1395 | 0.361 | 0 | 0.152 | 0 | 0 | 0 | 0 | 0.052 | 0.441 |
| acnB | pgi | 11127851 | 11125130 | 223283.PSPTO_3752 | 223283.PSPTO_0959 | 0 | 0 | 0 | 0 | 0 | 0 | 0 | 0.441 | 0.441 |
| PSPTO_2151 | PSPTO_0268 | 11126305 | 11124455 | 223283.PSPTO_2151 | 223283.PSPTO_0268 | 0 | 0 | 0.357 | 0 | 0.133 | 0 | 0 | 0.076 | 0.439 |
| PSPTO_3256 | PSPTO_2605 | 11127366 | 11126744 | 223283.PSPTO_3256 | 223283.PSPTO_2605 | 0.056 | 0 | 0 | 0 | 0.088 | 0 | 0 | 0.398 | 0.437 |
| hrpL | rpoD | 11125562 | 11124718 | 223283.PSPTO_1404 | 223283.PSPTO_0537 | 0 | 0 | 0 | 0 | 0.051 | 0.093 | 0 | 0.397 | 0.436 |
| irp3 | PSPTO_2134 | 11126738 | 11126288 | 223283.PSPTO_2599 | 223283.PSPTO_2134 | 0.084 | 0 | 0.339 | 0 | 0.068 | 0 | 0 | 0.118 | 0.435 |
| irp3 | PSPTO_2147 | 11126738 | 11126301 | 223283.PSPTO_2599 | 223283.PSPTO_2147 | 0.056 | 0 | 0.371 | 0 | 0.091 | 0 | 0 | 0.078 | 0.435 |
| cmaT | pchA | 11128781 | 11126734 | 223283.PSPTO_4712 | 223283.PSPTO_2595 | 0.079 | 0 | 0 | 0 | 0.384 | 0 | 0 | 0.083 | 0.434 |
| rpoS | hrpL | 11125722 | 11125562 | 223283.PSPTO_1565 | 223283.PSPTO_1404 | 0 | 0 | 0 | 0 | 0.051 | 0.093 | 0 | 0.395 | 0.434 |
| lpdA | rpoS | 11126353 | 11125722 | 223283.PSPTO_2201 | 223283.PSPTO_1565 | 0 | 0 | 0 | 0 | 0.064 | 0.345 | 0 | 0.15 | 0.433 |
| hopI1 | tpiA | 11128837 | 11128574 | 223283.PSPTO_4776 | 223283.PSPTO_4494 | 0.053 | 0 | 0.37 | 0 | 0.059 | 0 | 0 | 0.104 | 0.429 |
| cfa6 | irp5 | 11128759 | 11126736 | 223283.PSPTO_4686 | 223283.PSPTO_2597 | 0.084 | 0.031 | 0.36 | 0 | 0 | 0 | 0 | 0.104 | 0.428 |
| cmaB | cfa9 | 11128779 | 11128763 | 223283.PSPTO_4710 | 223283.PSPTO_4690 | 0.071 | 0 | 0.409 | 0 | 0 | 0 | 0 | 0 | 0.427 |
| gap-1 | mqo | 11125447 | 11125301 | 223283.PSPTO_1287 | 223283.PSPTO_1136 | 0 | 0 | 0 | 0 | 0.057 | 0 | 0 | 0.416 | 0.426 |
| hrpL | rpoC | 11125562 | 11124800 | 223283.PSPTO_1404 | 223283.PSPTO_0620 | 0.042 | 0 | 0 | 0 | 0.053 | 0.39 | 0 | 0.083 | 0.424 |
| PSPTO_2196 | cyoE | 11126348 | 11125487 | 223283.PSPTO_2196 | 223283.PSPTO_1329 | 0 | 0 | 0.154 | 0 | 0.268 | 0 | 0 | 0.143 | 0.422 |
| pchB | pvsA | 11126735 | 11126289 | 223283.PSPTO_2596 | 223283.PSPTO_2135 | 0 | 0 | 0 | 0 | 0.141 | 0 | 0 | 0.355 | 0.422 |
| eno-2 | lpdA | 11128692 | 11126353 | 223283.PSPTO_4616 | 223283.PSPTO_2201 | 0.086 | 0 | 0.187 | 0 | 0.185 | 0.096 | 0 | 0.104 | 0.42 |
| acnB | sdhC | 11127851 | 11126347 | 223283.PSPTO_3752 | 223283.PSPTO_2195 | 0.081 | 0 | 0 | 0 | 0.236 | 0 | 0 | 0.239 | 0.419 |
| PSPTO_5343 | aceE-2 | 11129399 | 11129064 | 223283.PSPTO_5343 | 223283.PSPTO_5005 | 0 | 0 | 0.418 | 0 | 0 | 0 | 0 | 0 | 0.418 |
| PSPTO_5560 | PSPTO_2145 | 11129608 | 11126299 | 223283.PSPTO_5560 | 223283.PSPTO_2145 | 0.064 | 0 | 0.389 | 0 | 0.065 | 0 | 0 | 0 | 0.418 |
| cmaC | cfa3 | 11128780 | 11128756 | 223283.PSPTO_4711 | 223283.PSPTO_4683 | 0 | 0 | 0.413 | 0 | 0 | 0 | 0 | 0 | 0.413 |
| sdhB | cyoE | 11126350 | 11125487 | 223283.PSPTO_2198 | 223283.PSPTO_1329 | 0 | 0 | 0 | 0 | 0.272 | 0 | 0 | 0.228 | 0.413 |
| sucB | iscA | 11126352 | 11125583 | 223283.PSPTO_2200 | 223283.PSPTO_1425 | 0 | 0 | 0.401 | 0 | 0 | 0 | 0 | 0.059 | 0.412 |
| sucB | glk | 11126352 | 11125449 | 223283.PSPTO_2200 | 223283.PSPTO_1289 | 0 | 0.383 | 0 | 0 | 0.05 | 0 | 0 | 0.075 | 0.41 |
| hrpE | hrpR | 11125544 | 11125537 | 223283.PSPTO_1386 | 223283.PSPTO_1379 | 0.332 | 0 | 0.153 | 0 | 0 | 0 | 0 | 0 | 0.41 |
| tolB | clpA | 11128064 | 11127463 | 223283.PSPTO_3972 | 223283.PSPTO_3353 | 0.053 | 0 | 0 | 0 | 0 | 0 | 0 | 0.401 | 0.409 |
| hopI1 | hopR1 | 11128837 | 11125055 | 223283.PSPTO_4776 | 223283.PSPTO_0883 | 0 | 0 | 0 | 0 | 0 | 0.094 | 0 | 0.375 | 0.409 |
| rpoN | hrpL | 11128533 | 11125562 | 223283.PSPTO_4453 | 223283.PSPTO_1404 | 0 | 0 | 0 | 0 | 0 | 0 | 0 | 0.409 | 0.409 |
| cfa6 | PSPTO_2148 | 11128759 | 11126302 | 223283.PSPTO_4686 | 223283.PSPTO_2148 | 0.056 | 0.023 | 0.364 | 0 | 0.06 | 0 | 0 | 0.075 | 0.408 |
| cfa2 | irp1 | 11128755 | 11126739 | 223283.PSPTO_4682 | 223283.PSPTO_2600 | 0.232 | 0 | 0 | 0 | 0.06 | 0 | 0 | 0.247 | 0.408 |
| PSPTO_4735 | cyoE | 11128800 | 11125487 | 223283.PSPTO_4735 | 223283.PSPTO_1329 | 0.054 | 0 | 0 | 0 | 0.056 | 0.39 | 0 | 0 | 0.407 |
| mtnC | algC | 11126201 | 11124280 | 223283.PSPTO_2047 | 223283.PSPTO_0083 | 0 | 0 | 0 | 0 | 0 | 0.406 | 0 | 0 | 0.406 |
| sucD | erpA | 11126355 | 11124785 | 223283.PSPTO_2203 | 223283.PSPTO_0605 | 0 | 0 | 0.223 | 0 | 0.077 | 0 | 0 | 0.237 | 0.405 |
| PSPTO_2853 | PSPTO_2145 | 11126983 | 11126299 | 223283.PSPTO_2853 | 223283.PSPTO_2145 | 0.064 | 0 | 0.374 | 0 | 0.065 | 0 | 0 | 0 | 0.404 |
| PSPTO_2146 | PSPTO_2145 | 11126300 | 11126299 | 223283.PSPTO_2146 | 223283.PSPTO_2145 | 0.404 | 0 | 0 | 0 | 0 | 0 | 0 | 0 | 0.404 |
| PSPTO_5343 | aceE-1 | 11129399 | 11127959 | 223283.PSPTO_5343 | 223283.PSPTO_3860 | 0 | 0 | 0.403 | 0 | 0 | 0 | 0 | 0 | 0.403 |
| PSPTO_4339 | recR | 11128421 | 11127739 | 223283.PSPTO_4339 | 223283.PSPTO_3638 | 0.403 | 0 | 0 | 0 | 0 | 0 | 0 | 0 | 0.403 |
| PSPTO_5560 | PSPTO_1207 | 11129608 | 11125370 | 223283.PSPTO_5560 | 223283.PSPTO_1207 | 0.056 | 0 | 0.314 | 0 | 0.051 | 0 | 0 | 0.143 | 0.402 |
| hrpK1 | hrpW1 | 11125563 | 11125531 | 223283.PSPTO_1405 | 223283.PSPTO_1373 | 0 | 0 | 0 | 0 | 0 | 0 | 0 | 0.402 | 0.402 |
| PSPTO_2602 | pvdE | 11126741 | 11126307 | 223283.PSPTO_2602 | 223283.PSPTO_2153 | 0.056 | 0 | 0.368 | 0 | 0 | 0 | 0 | 0.076 | 0.4 |
| hrpJ | hrpR | 11125561 | 11125537 | 223283.PSPTO_1403 | 223283.PSPTO_1379 | 0 | 0 | 0.152 | 0 | 0 | 0 | 0 | 0.322 | 0.4 |
